# Cell to whole organ global sensitivity analysis on a four-chamber heart electromechanics model using Gaussian processes emulators

**DOI:** 10.1371/journal.pcbi.1011257

**Published:** 2023-06-26

**Authors:** Marina Strocchi, Stefano Longobardi, Christoph M. Augustin, Matthias A. F. Gsell, Argyrios Petras, Christopher A. Rinaldi, Edward J. Vigmond, Gernot Plank, Chris J. Oates, Richard D. Wilkinson, Steven A. Niederer

**Affiliations:** 1 School of Biomedical Engineering and Imaging Sciences, King’s College London, London, United Kingdom; 2 National Heart and Lung Institute, Imperial College London, London, United Kingdom; 3 Medical University of Graz, Graz, Austria; 4 BioTechMed-Graz, Graz, Austria; 5 Johann Radon Institute for Computational and Applied Mathematics (RICAM), Linz, Austria; 6 Guy’s and St Thomas’ NHS Foundation Trust, London, United Kingdom; 7 University of Bordeaux, CNRS, Bordeaux, Talence, France; 8 IHU Liryc, Bordeaux, Talence, France; 9 Newcastle University, Newcastle upon Tyne, United Kingdom; 10 University of Nottingham, Nottingham, United Kingdom; 11 Alan Turing Institute, London, United Kingdom; University of Exeter, UNITED KINGDOM

## Abstract

Cardiac pump function arises from a series of highly orchestrated events across multiple scales. Computational electromechanics can encode these events in physics-constrained models. However, the large number of parameters in these models has made the systematic study of the link between cellular, tissue, and organ scale parameters to whole heart physiology challenging. A patient-specific anatomical heart model, or digital twin, was created. Cellular ionic dynamics and contraction were simulated with the Courtemanche-Land and the ToR-ORd-Land models for the atria and the ventricles, respectively. Whole heart contraction was coupled with the circulatory system, simulated with CircAdapt, while accounting for the effect of the pericardium on cardiac motion. The four-chamber electromechanics framework resulted in 117 parameters of interest. The model was broken into five hierarchical sub-models: tissue electrophysiology, ToR-ORd-Land model, Courtemanche-Land model, passive mechanics and CircAdapt. For each sub-model, we trained Gaussian processes emulators (GPEs) that were then used to perform a global sensitivity analysis (GSA) to retain parameters explaining 90% of the total sensitivity for subsequent analysis. We identified 45 out of 117 parameters that were important for whole heart function. We performed a GSA over these 45 parameters and identified the systemic and pulmonary peripheral resistance as being critical parameters for a wide range of volumetric and hemodynamic cardiac indexes across all four chambers. We have shown that GPEs provide a robust method for mapping between cellular properties and clinical measurements. This could be applied to identify parameters that can be calibrated in patient-specific models or digital twins, and to link cellular function to clinical indexes.

## Author summary

Cardiac function relies on complex links between the single cell and the whole organ. Digital twins or patient-specific models, e.g. computer models that replicate a patient’s heart, can help understanding these links in healthy or diseased states, and improving cardiac patient care. To build a patient-specific model, first we need to quantify which model parameters affect model outputs, to discard those that have little effect and to understand input-output interactions. This normally requires a lot of expensive model evaluations, making this type of analysis very challenging. We used Gaussian processes emulators (GPEs) to reduce the computational costs of our model. The heart simulator we approximated had 117 initial parameters, and was able to simulate whole heart electrical excitation and contraction, cellular dynamics, as well as the circulatory system and the interaction of the heart with the pericardium. Thanks to the GPEs, we were able to identify the most important 45 parameters at a feasible cost, and to study their effect on a wide range of clinically-measured biomarkers for cardiac function. Our analysis provides a comprehensive assay of how cellular function can impact the whole heart, and can be used to investigate a wide range of cardiac pathologies and treatment.

## Introduction

Heart function is a multi-scale process, going from sub-cellular mechanisms initiating single cell excitation and contraction, up to the whole organ and body. The heartbeat starts at the right atrium (RA) at the sino-atrial node, where the cells are able to excite spontaneously. The activation wave then travels to the left atrium (LA) and ventricles through the atrioventricular pathways and the His–Purkinje system, causing every myocyte to undergo the action potential, a rapid sequence of changes in the transmembrane potential. During the action potential, calcium ions are released into the cytosol, where they bind to the Troponin C located on the thin filament. This induces a conformational change in the thin filament which exposes the actin binding sites to the myosyn heads on the thick filament. The myosin heads then bind to the actin and pull the thin filament towards the centre of the sarcomere, leading to sarcomere shortening and ultimately myocyte contraction. Thanks to the structured myocyte orientation within the cardiac muscle, this results into a coordinated contraction of the whole heart, which then pumps oxygenated blood across the aortic valve and into the whole circulation.

Cardiac electromechanics models are increasingly used to study heart function in healthy and diseased states [1]. Initially, heart models focused on the left ventricle (LV) due to otherwise prohibitive computational costs. Recent developments in code and high performance computers have made it possible to simulate all four chambers, offering a more accurate representation of the interaction between the atria and the ventricles and a more physiological systolic motion [2–9]. Nevertheless, running large numbers of simulations remains a challenge due to numerical instabilities of highly non-linear mechanics, and due to high computational costs. This, combined with the large numbers of parameters of the model, makes performing global sensitivity analysis (GSA) and parameter inference very challenging. In this context, tools from statistics and machine learning can be used to approximate complex and expensive three-dimensional models [10, 11]. Fast model evaluations offered by these tools allow, firstly, to run a GSA to increase model credibility [12] and to identify important model parameters and, secondly, to infer the values of these parameters to fit clinical data at a fraction of the computational cost. This can finally enable the construction of patient-specific four-chamber models, or digital twins, that replicate how a specific subject progresses and responds to therapy. The ability of multi-scale simulations to link cellular processes to whole organ function has the potential to facilitate therapy decision-making, improve patient stratification, and provide novel mechanistic insight into treatment of a wide range of cardiac pathologies.

In order to perform parameter inference and build a digital twin of a human heart, we need to quantify the effect of the input parameters on the model outputs of interest. This not only allows us to exclude the parameters that have little to no effect on model outputs and can therefore be excluded from the analysis, but it also provides information about the extent of the model input-output interactions. In this paper, we used Gaussian processes emulators (GPE) to perform a GSA on a four-chamber electromechanics model. Our simulator accounts for the atrial and ventricular action potential, calcium transient, cross-bridge kinetics, whole heart electrical excitation and contraction, the effect of the pericardium and the coupling with the circulatory system. By applying GPEs and GSA on different components of the electromechanics simulation framework, we were able to reduce the number of parameters from 117 to 45. We used GPEs and history matching (HM) on the tissue electrophysiology and electromechanics cell simulators to identify the areas of the parameter space where the total ventricular and atrial activation times, and cellular calcium and active tension transients for atria and ventricles were physiological. Finally, we performed a GSA on the fully coupled framework with the remaining 45 parameters to quantify the effect of the model parameters on atrial and the ventricular function.

## Methods

Below, we summarize the clinical data and the four-chamber electromechanics framework used in this study. Tables 1, 2 and 3 list all simulator parameters we initially considered. We measured 17 of these parameters directly from clinical data (for example the heart rate and the valve areas). We then performed a GSA on different simulator sub-components to identify unimportant parameters and exclude these from further analysis.

**Table 1:** Simulator parameters. The first, second and third columns show the parameter name, range or value, and its meaning. The last column provides the original paper the symbol refers to. The blue rows indicate parameters that were estimated from clinical data, while the gray rows represent parameters the model outputs were insensitive to. HM in the second column indicates the parameters that were constrained by a history matching procedure on a sub-model (see S2, S3 and S4 Files). Abbreviations/symbols: CV = conduction velocity, FEC = fast endocardial conduction, Na^+^ = sodium, K^+^ = potassium, Ca^2+^ = calcium, Cl^-^ = chloride, SR = sarcoplasmic reticulum, DS = diadic space, HM = history matching.

**Electrophysiology—Tissue excitation simulator**			
CV_f,v_	HM	CV in the fibre direction of the ventricles	-
k_ft,v_	0.4	Anisotropy ratio of the ventricles	[18]
k_FEC_	HM	Scaling factor for the CV of the FEC layer	-
CV_f,a_	HM	CV in the fibre direction of the atria	-
k_ft,a_	0.4	Anisotropy ratio of the atria	[18]
k_BB_	HM	Scaling factor for the CV of the Bachmann bundle	-
AV_delay_	[100.0,200.0] ms	Atrioventricular delay	[37]
BCL	854 ms	Heartbeat duration	-
**ToR-ORd Model—Ventricular action potential simulator**			
**Ionic Conductances**			
*G*_Na_	11.7802	Conductance of the fast Na^+^ current	[20]
*G*_NaL_	0.0279	Conductance of the slow Na^+^ current	[20]
*G*_to_	0.16	Conductance of the transient outward K^+^ current	[20]
*P*_Ca_	HM	Conductance of the L-type Ca^2+^ current	[20]
*G*_Kr_	0.0321	Conductance of the rapid delayed K^+^ rectifier current	[20]
*G*_Ks_	0.0011	Conductance of the slow delayed K^+^ rectifier current	[20]
*G*_K1_	0.6992	Conductance of the inward K^+^ rectifier current	[20]
*G*_NCX_	HM	Conductance of the Na^+^-Ca^2+^ exchanger	[20]
*G*_NaK_	15.4509	Conductance of the Na^+^-K^+^ pump	[20]
*G*_Ca_	5e-04	Conductance of the sarcolemmal Ca^2+^ pump	[20]
*G*_Kb_	0.0189	Conductance of the background K^+^ current	[20]
*P*_Nab_	1.9239e-09	Conductance of the background Na^+^ current	[20]
*P*_Cab_	5.9194e-08	Conductance of the background Ca^2+^ current	[21]
*G*_ClCa_	0.2843	Conductance of the Ca^2+^-sensitive Cl^−^ current	[20]
*G*_Clb_	1.98e-03	Conductance of the background Cl^−^ current	[21]
**Calcium Handling**			
${\bar{J}}_{\text{rel}}$	1.5378	Multiplier of the release Ca^2+^ current from the SR	[20]
${\bar{J}}_{\text{up}}$	1.0	Multiplier of the uptake of Ca^2+^ into the SR	[20]
${\bar{I}}_{\text{NaCa,SS}}$	0.35	Fraction of the Na^+^-Ca^2+^ exchangers located in the subspace	[20]
${\bar{I}}_{\text{CaL,SS}}$	0.8	Fraction of the L-type channels located in the subspace	[20]
*α*_CaMK_	0.05	Phoshorilation rate of Ca^2+^/CaMK	[39]
*β*_CaMK_	0.00068	Dephoshorilation rate of Ca^2+^/CaMK	[39]
*CaMK*_o_	0.05	Fraction of active Ca^2+^/CaMK binding sites at equilibrium	[39]
$\bar{\left[\text{CMDN}\right]}$	0.05	Max calmodulin concentration	[39]
$\bar{\left[\text{TRPN}\right]}$	HM	Max troponin C concentration	[39]
$\bar{\left[\text{BSR}\right]}$	0.047	Max concentration of the SR binding sites in the DS	[39]
$\bar{\left[\text{BSL}\right]}$	1.124	Max concentration of the sarcolemmal binding sites in the DS	[39]
$\bar{\left[\text{CSQN}\right]}$	10.0	Max concentration of calsequestrin	[39]
*τ*_diff,Ca_	0.2	Diffusion rate of Ca^2+^ from the cytoplasm to the DS	[39]
*τ*_tr_	60.0	Diffusion rate of Ca^2+^ from the junctional to the network SR	[39]
**Courtemanche Model—Atrial action potential simulator**			
**Ionic Conductances**			
*g*_Na_	7.8	Conductance of the fast Na^+^ current	[22]
*g*_to_	0.1652	Conductance of the transient outward K^+^ current	[22]
*g*_Ca,L_	HM	Conductance of the L-type Ca^2+^ current	[22]
*g*_Kr_	0.0294	Conductance of the rapid delayed K^+^ rectifier current	[22]
*g*_Ks_	0.129	Conductance of the slow delayed K^+^ rectifier current	[22]
*g*_K1_	0.09	Conductance of the inward K^+^ rectifier current	[22]
*g*_b,Na_	0.000674	Conductance of the background Na^+^ current	[22]
*g*_b,Ca_	0.00113	Conductance of the background Ca^2+^ current	[22]
${\bar{g}}_{\text{Kur}}$	HM	Ultra-rapid rectifier K^+^ current scaling factor	[22]
*I*_NaCa(max)_	16	Na^+^-Ca^2+^ exchanger scaling factor	[22]
*I*_NaK(max)_	0.60	Max Na^+^-K^+^ pump current	[22]
*I*_p,Ca(max)_	0.275	Max sarcoplasmic Ca^2+^ pump current	[22]
**Calcium Handling**			
*I*_up(max)_	HM	Max Ca^2+^ uptake rate into the network SR	[22]
${\bar{k}}_{\text{rel}}$	30.0	Max Ca^2+^ release rate from junctional SR	[22]
[Cmdn]_max_	0.05	Total calmodulin concentration in cytoplasm	[22]
[Trpn]_max_	HM	Total troponin C concentration in cytoplasm	[22]
[Csqn]_max_	10.0	Total calsequestrin concentration in junctional SR	[22]
*τ*_tr_	180.0	Ca^2+^ transfer time constant	[22]

**Table 2:** Simulator parameters (continued). Abbreviations/symbols: Ca^2+^ = calcium, CaTRPN = calcium/troponin complex, LV = left ventricle, RV = right ventricle, fraction of unbound (U), and weakly (W) or strongly (S) bound binding sites.

**Land Model—Ventricular active tension simulator**			
*T*_ref_	HM	Reference isometric tension	[23]
*n*_Tm_	HM	Hill coefficient for Ca^2+^-troponin and U	[23]
*n*_TRPN_	HM	Ca^2+^-troponin cooperativity	[23]
*k*_TRPN_	0.1	Unbinding rate of Ca^2+^ from troponin	[23]
*A*_eff_	HM	Scale for distortion due to velocity of contraction	[23]
*k*_u_	HM	Transition rate from blocked to unblocked binding site	[23]
*β*_0_	2.3	Length-dependence parameter for tension development	[23]
*β*_1_	-2.4	Length-dependence parameter for Ca^2+^ sensitivity	[23]
*γ*_s_	0.0085	Distortion rate of strongly bound cross-bridges	[23]
*γ*_w_	0.615	Distortion rate of weakly bound cross-bridges	[23]
*ϕ*	2.23	Distortion decay	[23]
*ca*_50_	HM	Reference Ca^2+^ sensitivity	[23]
*ν*	7.0	Scaling factor for U to W cross-bridges transition rate	[23]
*μ*	HM	Scaling factor for W to S transition rate	[23]
TRPN_50_	HM	CaTRPN when 50% of cross-bridges are blocked	[23]
*r*_s_	HM	Steady-state duty ratio	[23]
*r*_w_	HM	Steady-state ratio between W and S	[23]
**Land Model—Atrial active tension simulator**			
*T*_ref_	HM	Reference isometric tension	[23]
*n*_Tm_	HM	Hill coefficient for Ca^2+^-troponin and U	[23]
*n*_TRPN_	HM	Ca^2+^-troponin cooperativity	[23]
*k*_TRPN_	0.1	Unbinding rate of Ca^2+^ from troponin	[23]
*A*_eff_	HM	Scale for distortion due to velocity of contraction	[23]
*k*_u_	1.0	Transition rate from blocked to unblocked binding site	[23]
*β*_0_	2.3	Length-dependence parameter for tension development	[23]
*β*_1_	-2.4	Length-dependence parameter for Ca^2+^ sensitivity	[23]
*γ*_s_	0.0085	Distortion rate of strongly bound cross-bridges	[23]
*γ*_w_	0.615	Distortion rate of weakly bound cross-bridges	[23]
*ϕ*	HM	Distortion decay	[23]
*ca*_50_	HM	Reference Ca^2+^ sensitivity	[23]
*ν*	7.0	Scaling factor for U to W transition rate	[23]
*μ*	HM	Scaling factor for W to S transition rate	[23]
TRPN_50_	HM	CaTRPN when 50% of cross-bridges are blocked	[23]
*r*_s_	HM	Steady-state duty ratio	[23]
*r*_w_	HM	Steady-state ratio between W and S	[23]
**Guccione model—Ventricular passive mechanics**			
*a*	[0.5,1.5] kPa	Bulk myocardium stiffness	Eq (2), [9]
*b*_f_	8.0	Stiffness in the fibre direction	Eq (2)
*b*_ft_	4.0	Stiffness in the fibre-transverse shear planes	Eq (2)
*b*_t_	[1.5,4.5]	Stiffness in the transverse plane	Eq (2), [9, 40]
**Passive Mechanics—Atrial passive mechanics**			
*a*	[1.5,2.5] kPa	Bulk myocardium stiffness	Eq (2), [9, 41]
*b*_f_	[4.0,12.0]	Stiffness in the fibre direction	Eq (2), [9, 40]
*b*_ft_	4.0	Stiffness in the fibre-transverse shear planes	Eq (2)
*b*_t_	[1.5,4.5]	Stiffness in the transverse plane	Eq (2), [9, 40]
**LV-RV scaling factors**			
*T*_ref,LvRv_	[0.5,1.0]	Scaling factor for *T*_ref_ in the RV vs the LV	[41]
*a*_LvRv_	[1.0,2.0]	Scaling factor for *a* in the RV vs LV	[41]

**Table 3:** Simulator parameters (continued).

**CircAdapt—Circulatory system simulator**			
**Cavities**			
*V*_*LV*,*wall*_	93.6 mL	Wall volume of the left ventricle	[29, 32]
*V*_*RV*,*wall*_	37.6 mL	Wall volume of the right ventricle	[29, 32]
*V*_*SV*,*wall*_	32.4 mL	Wall volume of the septum	[29, 32]
*V*_*LA*,*wall*_	25.7 mL	Wall volume of the left atrium	[29, 32]
*V*_*RA*,*wall*_	22.2 mL	Wall volume of the right atrium	[29, 32]
**Valves**			
$A_{MV}^{\text{open}}$	508.3 mm^2^	Mitral valve open orifice area	[29, 32]
$A_{TV}^{\text{open}}$	508.3 mm^2^	Tricuspid valve open orifice area	[29, 32]
$A_{AV}^{\text{open}}$	336.7 mm^2^	Aortic valve open orifice area	[29, 32]
$A_{PV}^{\text{open}}$	336.7 mm^2^	Pulmonary valve open orifice area	[29, 32]
$A_{SO}^{\text{open}}$	518.7 mm^2^	Systemic outlet open orifice area	[29, 32]
$A_{PO}^{\text{open}}$	296.1 mm^2^	Pulmonary outlet open orifice area	[29, 32]
**Tubes**			
$p_{\text{Ao}}^{0}$	1.0	Scaling factor for reference aortic pressure	[29, 32]
$p_{\text{Pa}}^{0}$	1.0	Scaling factor for reference pulmonary arterial pressure	[29, 32]
$p_{\text{Ve}}^{0}$	1.0	Scaling factor for reference systemic veins pressure	[29, 32]
$p_{\text{Pve}}^{0}$	1.0	Scaling factor for reference pulmonary veins pressure	[29, 32]
*l*_Ao_	[300.0,500.0] mm	Length of the aorta	[29, 32]
*l*_Pa_	200.06 mm	Length of the pulmonary artery	[29, 32]
*l*_Ve_	400.11 mm	Length of the systemic veins	[29, 32]
*l*_Pve_	200.06 mm	Length of the pulmonary veins	[29, 32]
*k*_Ao_	[6.0,10.0]	Stiffness of the aorta	[29, 32]
*k*_Pa_	8.0	Stiffness of the pulmonary artery	[29, 32]
*k*_Ve_	10.0	Stiffness of the veins	[29, 32]
*k*_Pve_	10.0	Stiffness of the pulmonary veins	[29, 32]
$A_{\text{Ao}}^{\text{wall}}$	142.7 mm^2^	Wall area of the aorta	[29, 32]
$A_{\text{Pa}}^{\text{wall}}$	142.7 mm^2^	Wall area of the pulmonary artery	[29, 32]
$A_{\text{Ve}}^{\text{wall}}$	83.7 mm^2^	Wall area of the veins	[29, 32]
$A_{\text{Pve}}^{\text{wall}}$	64.7 mm^2^	Wall area of the pulmonary veins	[29, 32]
**Systemic Circulation**			
*q* ^ref^	82 mL/s	Reference systemic flow	[29, 32]
$\Delta p_{\text{sys}}^{\text{ref}}$	90.01 mmHg	Reference pressure drop across the systemic circulation	[29, 32]
$\Delta p_{\text{pulm}}^{\text{ref}}$	11.25 mmHg	Reference pressure drop across the pulmonary circulation	[29, 32]
*R*_sys_	[1.0,4.0]	Systemic resistance scaling factor	[29, 32]
*R*_pulm_	[1.0,4.0]	Pulmonary resistance scaling factor	[29, 32]
**Boundary Conditions**			
k_peri_	[0.5,2.0] kPa/mm	Pericardium spring stiffness	[4]
EDP_shift,LV_	[3.0,9.0] mmHg	Shift applied to the left ventricular end-diastolic pressure	[42]
EDP_unload,RV_	[3.0,8.0] mmHg	Right ventricular end-diastolic pressure	[42]

### Clinical data

The long-term goal of this work is to construct a digital twin of a specific subject. Therefore, we focused this study on one patient. The clinical data were gathered from a 78 yo female heart failure (HF) patient with atrial fibrillation (AF) selected for cardiac resynchronisation therapy device upgrade from RV pacing to biventricular pacing. Right ventricular (RV) pacing was therefore the baseline rhythm, with a QRS duration of 200 ms measured from a 12-lead electrocardiogram (ECG). The patient underwent ECG-gated CT prior to the upgrade procedure, providing ten CT frames over a cardiac cycle. During the upgrade procedure, the LV pressure was invasively recorded through a pressure wire. After the ectopic baseline beats were removed, 30 beats were left to characterise the patient’s LV haemodynamics. The LV end-diastolic pressure (EDP) and peak in pressure were 2.8±3.2 mmHg and 124.2±7.4 mmHg, while the basic cycle length and the LV pressure systolic duration were 854±9 ms and 467±15 ms, respectively.

### Four-chamber electromechanics framework

#### Four-chamber heart geometry

**Fig 1:**
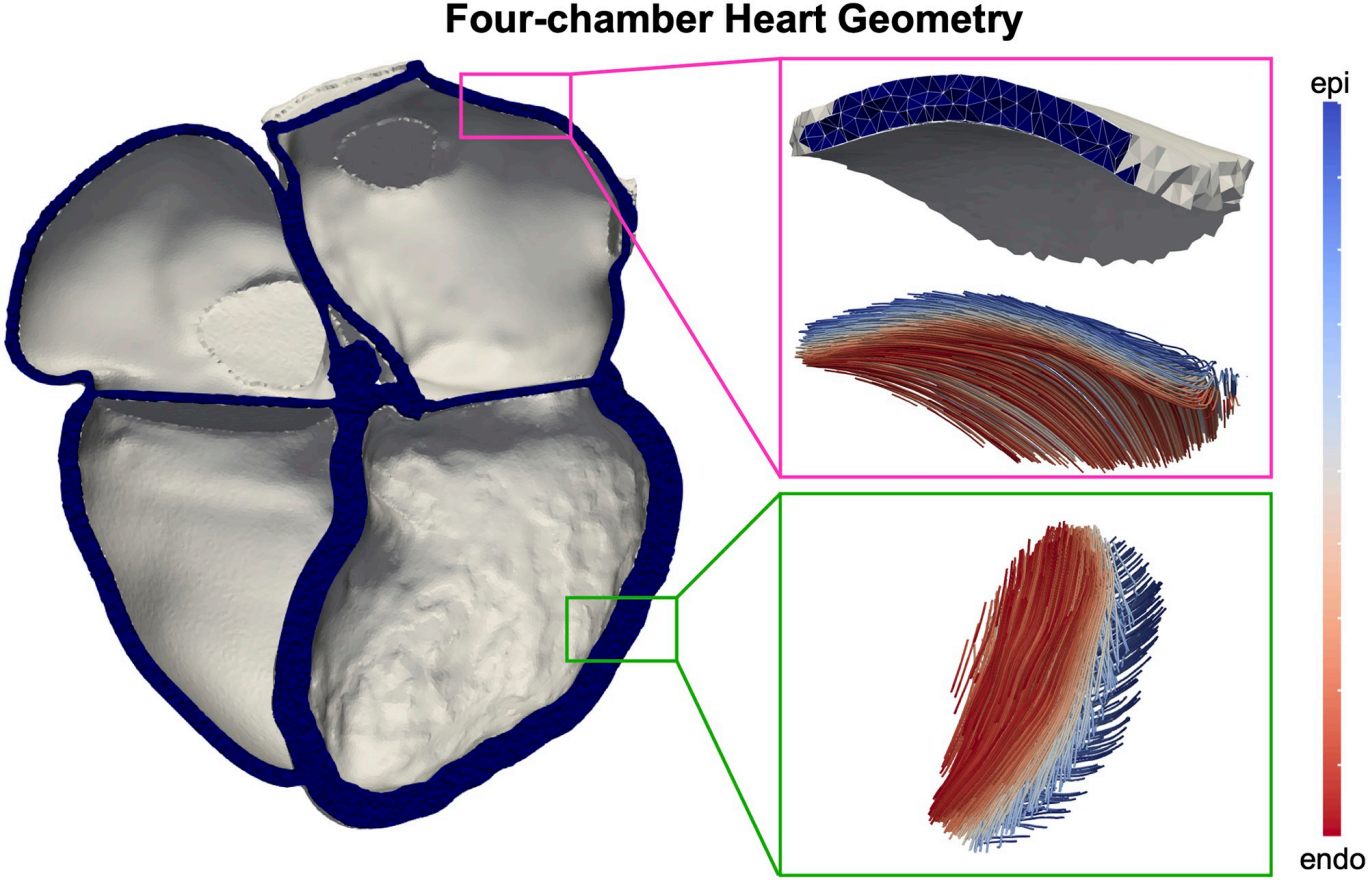
Four-chamber heart geometry. Left: patient-specific four-chamber heart mesh. Right: refines atria, atrial and ventricular transmural myofiber orientation from endocardium (red) to epicardium (blue).

The four-chamber heart geometry was generated from the end-diastolic computed tomography (CT). The pipeline to segment and generate 1mm linear tetrahedral four-chamber meshes was described previously [2, 3]. The atria were refined with the resample algorithm from meshtool [13] to have at least 3 elements across the wall thickness to reduce locking effects. The ventricles were assigned with a myofibre orientation using Bayer’s rule-base method [14] (Fig 1, bottom right), with the fibre and sheet angles at the endocardium and epicardium set to be +60° and -60° [4], and -65° and +25° [14], respectively. Atrial myofibre orientation was assigned by computing universal atrial coordinates on the atria and mapping an ex-vivo diffusion tensor MRI dataset onto the endocardial and the epicardial surfaces (Fig 1, top right) [15, 16]. The transmural fibre orientation was then set to be the endocardial and the epicardial orientation for elements below and above 50% of the wall thickness, respectively.

#### Electromechanics simulation framework

The electrical activation of the heart was simulated with a reaction-Eikonal model without diffusion [17]. The Eikonal model in Eq (1) solves for local activation times *t*_*a*_(**x**) at node location **x** given **V**(**x**) containing the squared local conduction velocities (CV) in the fibres, sheet and normal to sheet directions, and sites of initial activation Γ, which activate at a prescribed time *t*_0_:

$$\begin{array}{cc} \sqrt{\nabla t_{a}{\left(\text{x}\right)}^{T}\text{V}\left(\text{x}\right)\nabla t_{a}\left(\text{x}\right)} & =1\;\text{x}\in \Omega \;\\ t_{a}\left(\text{x}\right) & =t_{0}\;\text{x}\in \Gamma \;. \end{array}$$ (1)

**Fig 2:**
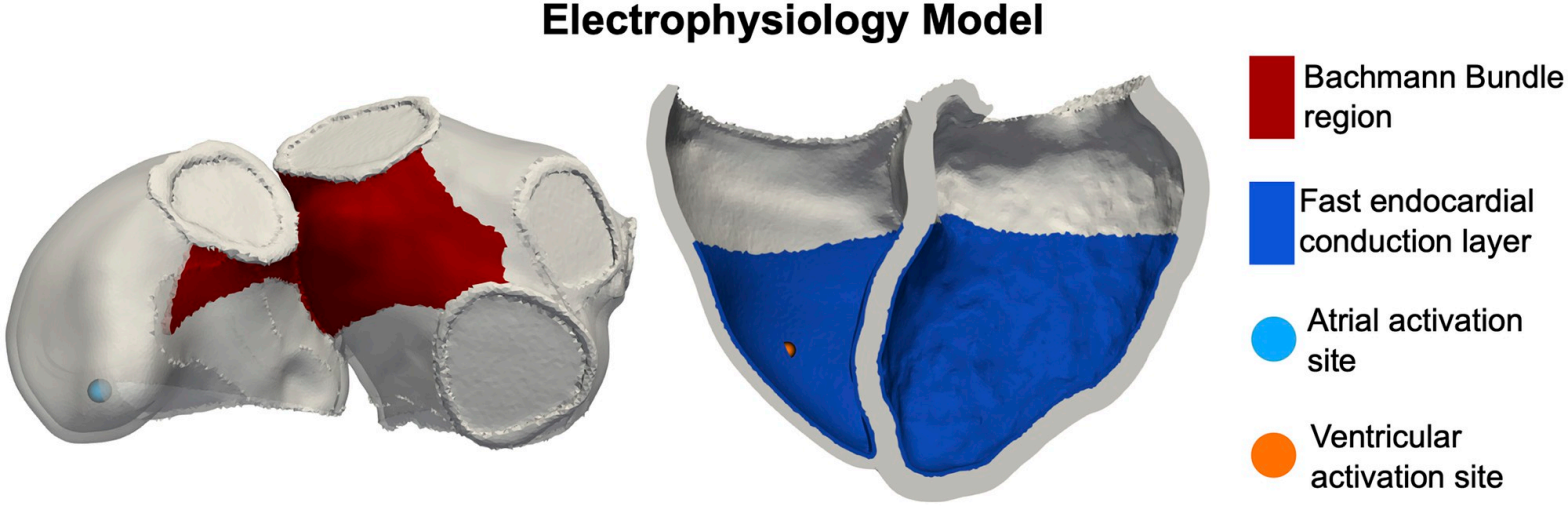
Electrophysiology simulations. Left: atria with the region representing the Bachman bundle (red) and the atrial activation site (light-blue circle). Right: ventricles with the fast endocardial conduction layer (blue) and the ventricular activation site (orange circle).

Atrial and ventricular myocardium were represented as transversely isotropic conductive regions and were assigned CV in the fibre direction (CV_f,V_ and CV_f,A_) and an anisotropy ratio (k_ft,V_ and k_ft,A_), respectively. The remaining regions were passive. To represent fast endocardial activation due to the His–Purkinje system, we defined a one-element thick endocardial layer extending up to 70% in the apico-basal direction in the ventricles [18, 19] with faster conduction velocity (CV) compared to the rest of ventricular myocardium of a factor *k*_FEC_ (Fig 2, right). Equivalently, to account for the Bachmann bundle, we defined a region between the LA and the RA with fast CV compared to the rest of the atrial myocardium of a factor *k*_BB_ (Fig 2, left) [16]. To fully control the atrioventricular (AV) delay, we defined a passive region along the AV plane to insulate the atria from the ventricles. Atrial activation was then initiated at the location of the RA lead, while ventricular activation was initiated at the RV lead location with a delay defined by the AV delay, included as a free parameter in the simulator (AV_delay_). The RA and RV lead locations were selected by segmenting the pacemaker leads from the CT image by thresholding the image intensity.

The action potential of ventricular and atrial myocytes was simulated with the ToR-ORd model with dynamic intracellular cloride [20, 21] and the Courtemanche model [22], respectively. At each point in the domain **x**, a foot current inducing the initial increase in the transmembrane potential *V*_m_ is imposed as stimulus to locally activate the cell membrane, at a local activation time *t*_*a*_(**x**) computed with the Eikonal model [17]. The intracellular calcium transient computed by the ionic model was then provided as input to the Land contraction model [23] to compute the active tension transient in the atria and ventricles. For simplicity, we assumed that active contraction occurred only in the fibre direction.

In the cell electrophysiology models, we selected model parameters that have a biophysical interpretation but can be, at the same time, considered uncertain [24]. Ion channel conductances, pumps and exchangers leading to transmembrane currents depend on protein expression. Therefore, these can be assumed to be different between individual cells and, since they relate to channel biophysics, they can be considered to be epistemic uncertainty. On the other hand, the natural variability in other parameters such as gating kinetics of ion channels, which we did not consider, can be attributed to aleatoric uncertainty, which is irreducible [25]. Based on this, we initially included all ion channel conductances and all calcium kinetics parameters (Table 1). All 17 parameters for the Land model were included in the initial analysis for both atria and ventricles. Tables 1 and 2 summarise the parameters we considered and their meaning.

Prior to the whole organ simulations, the ToR-ORd-Land and the Courtemanche-Land cell models were ran for 500 beats at a basic cycle length of 854.0 ms, consistent with the patient’s heart rate, to bring the models to a steady state. The cell models were then initialised at a steady state in the whole heart simulations.

Passive material properties of atria and ventricular myocardium were represented with a transversely isotropic Guccione model [26]:

$$\begin{array}{cc} \Psi (\text{E}) & =\frac{a}{2}[e^{Q}-1]+\frac{\kappa }{2}(\text{log}\;J{)}^{2}\\ Q & =b_{\text{f}}E_{\text{ff}}^{2}+2b_{\text{ft}}\left(E_{\text{fs}}^{2}+E_{\text{fn}}^{2}\right)+b_{\text{t}}\left(E_{\text{ss}}^{2}+E_{\text{nn}}^{2}+2E_{\text{sn}}^{2}\right)\;, \end{array}$$ (2)

where *J* is the determinant of the deformation gradient, **E** represents the Cauchy-Green strain tensor and f, s and n are the fibre, sheet and normal to sheet directions. Parameters *a*, *b*_f_, *b*_ft_ and *b*_t_ are the stiffness parameters, and *κ* = 1000 kPa is the bulk modulus, penalising volume change and therefore enforcing near incompressibility [27, 28]. All material stiffness parameters for the atria and the ventricles were initially included as free simulator parameters. Passive material properties of all other tissues were represented with a Neo-Hookean model, with the parameter stiffness set according to previous studies [2, 3].

#### Mechanics boundary conditions

**Fig 3:**
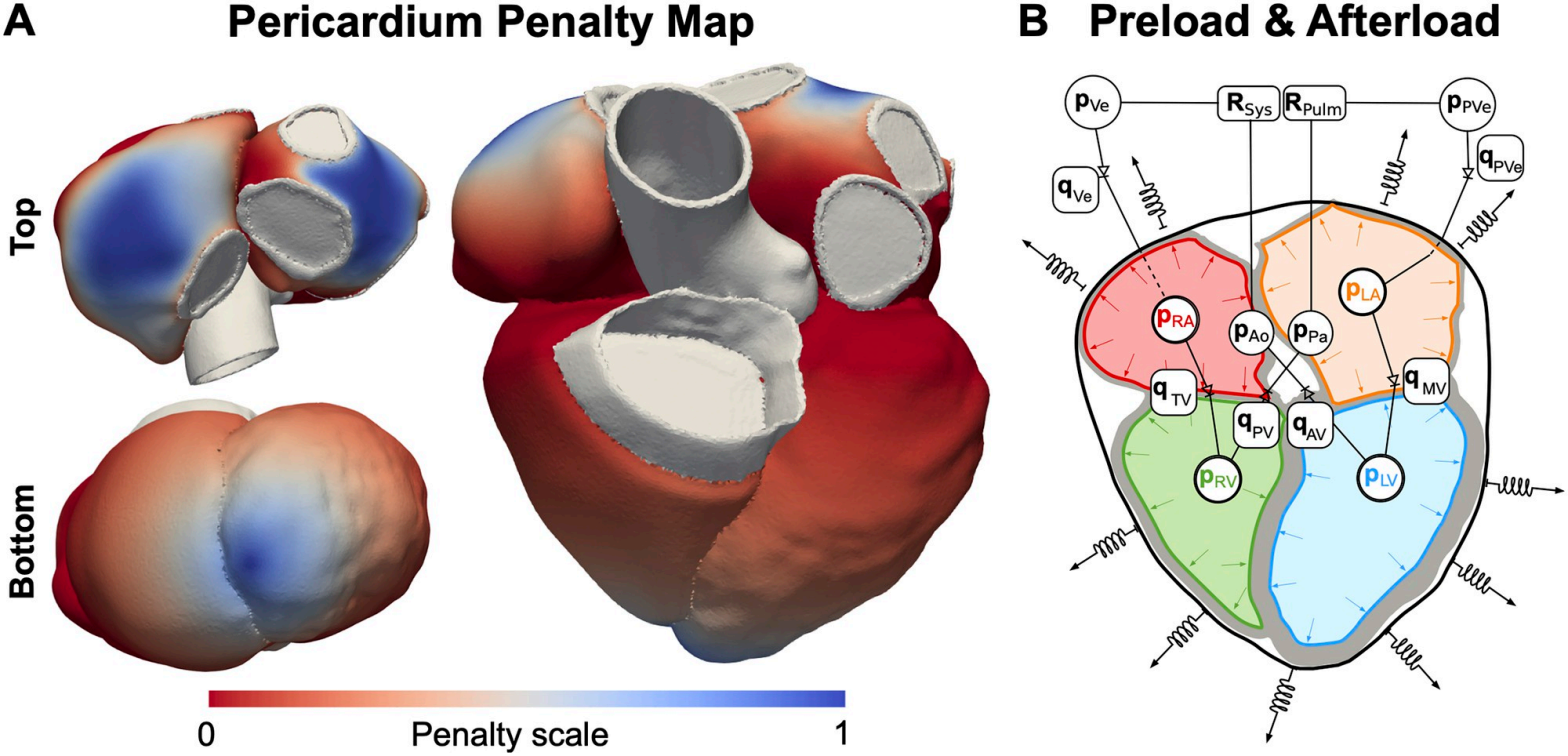
Mechanics boundary conditions. **A** Penalty map scaling the normal spring stiffness for the effect of the pericardium. **B** Afterload and preload boundary conditions represented with CircAdapt. Symbols and abbreviations: **p** = pressure, **R** = resistance, **q** = flow across a valve, LV = left ventricle, RV = right ventricle =, LA = left atrium, RA = right atrium, Ao = aorta, Pa = pulmonary artery, Ve = veins, PVe = pulmonary veins, sys = systemic, pulm = pulmonary, MV = mitral valve, TV = tricuspid valve, AV = aortic valve, PV = pulmonary valve.

We represented the effect of the pericardium on the heart with normal springs, as described in [2, 29]. The spring stiffness *k*_peri_ was included as a free simulator parameter. This value was scaled on the ventricles according to a map derived from motion data [3], to constrain the motion of the apex but not the base, allowing for physiological AV plane downward displacement during ventricular systole. A similar analysis on the atria, described in [30], showed that the roof of the atria moved the least, while the regions around the AV plane moved the most as they were stretched down by the contracting ventricles. We therefore defined a scaling map on the atria to include this constraint in the model, by assigning maximum penalty to the roof of the atria and zero penalty towards the AV plane (Fig 3A). In addition, we applied omni-directional springs to the right inferior and superior pulmonary veins and at the superior vena cava rings. The stiffness of these springs was fixed to 1.0 kPa/*μ*m and was not considered as a free parameter as this was only imposed to constrain the motion during the unloading of the mesh, performed in the absence of the pericardium.

The three-dimensional (3D) four-chamber electromechanics model was coupled with CircAdapt [31, 32] (Fig 3B), a closed-loop zero-dimensional (0D) model representing the following components of the circulatory system: aorta, pulmonary artery, veins, systemic and pulmonary peripheral resistances, the four cardiac valves (aortic, pulmonary, mitral and tricuspid) and flows across the pulmonary veins into the LA and across the systemic veins into the RA. The monolithic 3D solid–0D fluid coupling method is described in detail by Augustin et al. [29]. Briefly, the pressures of the LA, LV, RA and RV were included as additional unknowns to the monolithic scheme, and the following equations are added to the mechanics equilibrium equations:

$$\begin{array}{cc} V_{\text{LV}}^{\text{3D}}\left(\text{u},t\right)-V^{\text{0D}}\left(p_{\text{LV}},t\right) & =0\;\\ V_{\text{RV}}^{\text{3D}}\left(\text{u},t\right)-V^{\text{0D}}\left(p_{\text{RV}},t\right) & =0\;\\ V_{\text{LA}}^{\text{3D}}\left(\text{u},t\right)-V^{\text{0D}}\left(p_{\text{LA}},t\right) & =0\;\\ V_{\text{RA}}^{\text{3D}}\left(\text{u},t\right)-V^{\text{0D}}\left(p_{\text{RA}},t\right) & =0\;, \end{array}$$

where *V*^3D^ and *V*^0D^ are the volumes of the cavity computed from the deforming 3D mesh and predicted by the 0D model, respectively, *t* is the time and **u** is the displacement field. Table 3 lists all CircAdapt parameters that were included in the initial analysis. In this study, CircAdapt adaptation rules were not applied.

The ventricles of the end-diastolic mesh were unloaded from an end-diastolic LV and RV pressure, while the atria were not unloaded, under the assumption that the active tension in the atrial myocardium balances the pressure [23]. The measured mean LV end-diastolic pressure was only 2.8 mmHg, which was not enough to achieve physiological end-diastolic strains. This may reflect an offset error or drift of the pressure catheter measurements. To account for this potential artifact, we introduced an additional free offset parameter called EDP_shift,LV_ to shift the LV EDP during unloading. This not only allowed the simulations to achieve higher end-diastolic strains, but it also accounted for uncertainty on the available clinical measurements. The RV end-diastolic pressure was not available, so we added it as a free simulator parameter (EDP_unload,RV_). The unloading was performed with a backward displacement method [33]. During the unloading, we did not apply boundary conditions for the effect of the pericardium. Then, prior to the start of the 3D-0D coupled simulation, we reloaded the ventricles to retrieve the end-diastolic mesh while the atrial pressure was initialised at 0 mmHg. The simulation was then started at end-diastole and the pericardium boundary conditions were turned on. To minimise the effect of these initial conditions, we ran all simulations for 5 beats, to reach a near-to-steady-state behaviour.

#### Patient-specific parameters

We used the available clinical data to estimate 17 simulator parameters, therefore reducing the computational cost of the final GSA (light-blue rows, Tables 1 and 3). First, a CT motion tracking algorithm [34] was applied to track the motion of the patient’s heart, and the displacement field was used to deform the patient-specific end-diastolic mesh and derive an LV volume transient. This provided the patient’s LV end-diastolic and end-systolic volumes, and therefore an LV stroke volume of 70 mL.

The 30 baseline LV pressure beats were used to compute an average basic cycle length (BCL) of 854 ms, corresponding to a heart rate (HR) of 70 bpm. The ratio between the SV and the BCL provided the systemic flow *q*^ref^ = 82 mL/s in the CircAdapt model. The wall volumes of the LV and RV free walls, septum, LA and RA were computed from the tetrahedral mesh. The mitral valve open orifice area was computed by selecting points along the mitral valve leaflets from the end-diastolic CT, a sphere was fitted to the selected points and the radius was measured assuming a circular orifice shape (consistently with CircAdapt). The aortic valve open orifice area was measured with the same approach, by selecting points on the aortic valve leaflets on the end-systolic CT frame. The leaflets of the tricuspid and pulmonary valves were not visible on the CT, so their open orifice area was assumed to be the same as the mitral and the aortic valve, respectively, again consistent with the CircAdapt model. The systemic and pulmonary orifices areas were computed as the average of the cross-sectional area of the superior and inferior vena cava, and of all pulmonary veins, respectively, all computed from the end-diastolic CT. The reference area for the aorta, the pulmonary artery, the systemic veins and the pulmonary veins tubes were set to be the same as the corresponding adjacent valve. The aortic and the pulmonary artery wall area were then computed assuming a circular cross-section (consistent with CircAdapt) and a wall thickness of 2 mm [35]. The systemic and the pulmonary veins were assigned a wall thickness of 1 mm, because the veins are thinner due to the presence of less smooth muscles compared to arteries [36] as they operate at lower pressures. The blue rows in Table 3 show the values of the parameters that were computed from the patient-specific data and mesh.

#### Numerical methods

As shown in Tables 1–3, the final analysis included 45 parameters. In order to achieve accurate GPE accuracy, we selected *N* = 500 samples for GPE training. Due to simulator complexity, this requires a lot of computational resources. We therefore selected the numerical tolerances in the mechanics simulations to reduce as much as possible the computational cost of the electromechanical simulations.

We assumed that 5 beats were enough to achieve a near steady state solution. The first three beats were ran by setting the number of Newton iterations to 1. As shown by Augustin et al. [29], this approach brings the simulation closer to a steady state before solving non-linear mechanics more accurately with more Newton iterations. The last two beats were ran by setting the number of Newton iterations to 2 to have a better approximation of the stretch rate for the cell model. We also increased the tolerance for the solution of the linearised system to 10^-4^ for all beats. In S1 File, we show that these settings have a limited effect on the pressure-volume dynamics simulated by the model, with differences in the pressure and volume features always below 3%, while allowing up to 3 times speedup in the simulation time. Finally, the time step for the ionic models and the mechanics were set to 0.02 ms and 1.0 ms, respectively.

All simulations were run with the cardiac arrhythmia research package (CARP) [37] on a supercomputer on 512 cores. Details about the software implementation is provided in [17, 29].

### Emulators, global sensitivity analysis and history matching

GPEs, GSA and HM methods used in this study were based on Longobardi et al. [11], using code available on GitHub (https://github.com/stelong/Historia.git). Briefly, a GPE provides a statistical model for how a scalar simulator output *f*(**x**) varies as a function of *D* input parameters **x** = (*x*_1_, …, *x*_*D*_). Under our GPE, the simulator outputs are modelled as being jointly Gaussian with prior mean and covariance

$$E[f(\text{x})]=\beta_{0}+\sum_{i=1}^{D}\beta_{i}x_{i}\;$$ (3)

$$C[f\left(\text{x}\right),f\left({\text{x}}^{\prime }\right)]=k(\text{x},{\text{x}}^{\prime })\;,$$ (4)

where *k*(**x**, **x**′) is a positive-definite kernel, and *β*_0_, …, *β*_*D*_ are the degrees of freedom of the GPE, which need to be estimated. In this case, we chose the exponentiated quadratic kernel, also known as squared exponential or the Gaussian kernel, because it offers a parsimonious model even in situations where the data is limited and noisy [43]. In S8 File, we show that GPE accuracy was not affected when using Matérn rather than an exponentiated quadratic kernel. We trained one GPE for each scalar simulator output, using a training set D consisting of simulator outputs computed for a variety of different input parameter configurations as described in [11].

GPE accuracy was evaluated by performing a 5-fold cross-validation. For each split, we computed the coefficient of determination *R*^2^ and the independent standard error (ISE) between a vector of outputs **Y** and the posterior predictions of the GPE with expected value and standard deviation E[f(x)|D] and σ[f(x)|D] as:

$$R^{2}=1-\frac{\text{RSS}}{\text{TSS}}$$ (5)

ISE=#{|E[f(x)|D]-Y|<2σ[f(x)|D]}, (6)

where RSS and TSS are the sum of squared residuals and the total sum of squares, respectively. *R*^2^ provides an error on the point-wise estimate of the GPE, with values close to 1 indicating a low error between predictions and observations. The ISE evaluates the uncertainty quantification of the GPEs, and represents the number of data points that fall within two standard deviations away from the average prediction. As it is presented as a percentage over all evaluated points, an ISE close to 100% indicates that, for most points, the distance between the GPE prediction and the corresponding observation is within the GPE uncertainty. Once the GPEs were validated, we trained an additional GPE for each output using all available simulations that was then used for GSA and HM.

Using the GPEs to predict the simulator outputs, we performed a Sobol variance based GSA to quantify the importance of each parameter [44]. We computed the total effects *S*_*T*_ using the Saltelli method with the SALib Python library. A Saltelli sampling [44, 45] was used to generate a convenient structure of the samples to efficiently approximate the sensitivity indices as described in S7 File. When computing the sensitivity indices of each output, we sampled the posterior distribution of each GPE with *N* = 1000 samples, computed the total effect for each sample and taken the mean of the total effects. To an extent, this allowed us to account for GPE uncertainty in the GSA.

We used the total effects to rank the parameters from most to least important by computing the maximum total effect across all outputs. The maximum total effects for each parameter were normalised to that they would sum up to 1 (representing 100% of output variance), and the parameters that collectively explained 90% of variation in simulator output were included in the analysis, while the others were fixed to values provided in Tables 1–3, since the simulator outputs were not sensitive to how these parameters are specified.

**Fig 4:**
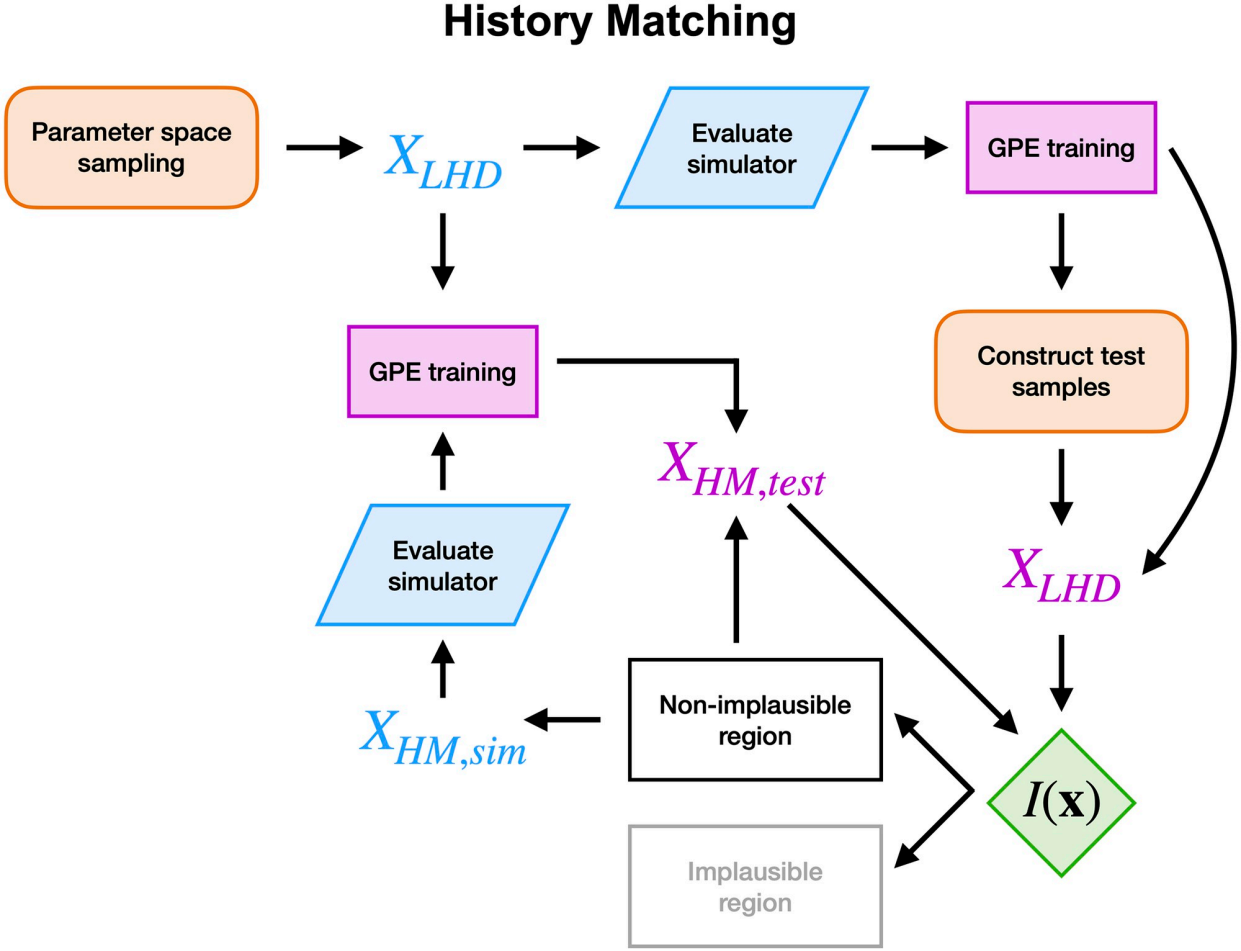
History matching. Orange, blue and purple boxes indicate Latin hypercube sampling, simulator evaluation and GPE training, respectively. Blue and purple **X** indicate points where the simulator or the GPEs are evaluated.

In order to make sure that the ToR-ORd-Land, Courtemanche-Land and the Eikonal models operated within physiological ranges, and to avoid wasting computational effort in the electromechanics simulator, we used HM. HM with GPEs is thoroughly explained in Longobardi et al. [11], and Fig 4 shows a schematic of our approach. First, the parameter space is explored with a Latin hypercube design. This provides a set of samples in the parameter space where the simulations are performed. These samples and outputs are then used to train a GPE. A test parameter set of 100,000 samples is then constructed with Latin hypercube sampling, and the GPE is evaluated at each sample. For each test sample, we evaluate the implausibility measure *I*(**x**) as:

$$I(\text{x})=\underset{i=1,\ldots ,N_{\text{output}}}{\text{max}}\;I_{i}\left(\text{x}\right)$$ (7)

$$I_{i}^{2}\left(\text{x}\right)=\frac{{\left(\text{E}\left[f_{i}\left(\text{x}\right)|D\right]-\mu_{i}\right)}^{2}}{\sigma^{2}\left[f\left(\text{x}\right)|D\right]+\sigma_{i}^{2}}\;,$$ (8)

where *f*_*i*_ is the GPE trained for the *i*th output. Scalars *μ*_*i*_ and *σ*_*i*_ are the measured target value and standard deviation for the *i*th output, available from the literature or the clinical data. By defining a threshold *I*_th_ on the implausibility measure *I*(**x**), the test samples are split in plausible (*I*(**x**) below the threshold) and implausible (*I*(**x**) above the threshold). From the implausible region, we then select a number N_simul_ samples where the simulator is evaluated, and we construct a new test parameter set N_test_ = 100000 for the next HM iteration (or wave). The GPEs are re-trained by adding the new simulated samples to the training set, and evaluated on the test set constructed at the previous wave. For each wave, we report the percentage of non-implausible points, the mean and maximum *I* and the mean and maximum ratio of GPE variance prediction over the measured variance *σ*^2^ (*V*_ratio_). The latter quantity gives a measure of how uncertain the GPEs are, relative to the uncertainty on the data we are trying to match. We set the final implausibility threshold to 3, based on Pukelsheim’s 3-sigma rule [46].

### Sensitivity analysis and history matching on simulator components

**Fig 5:**
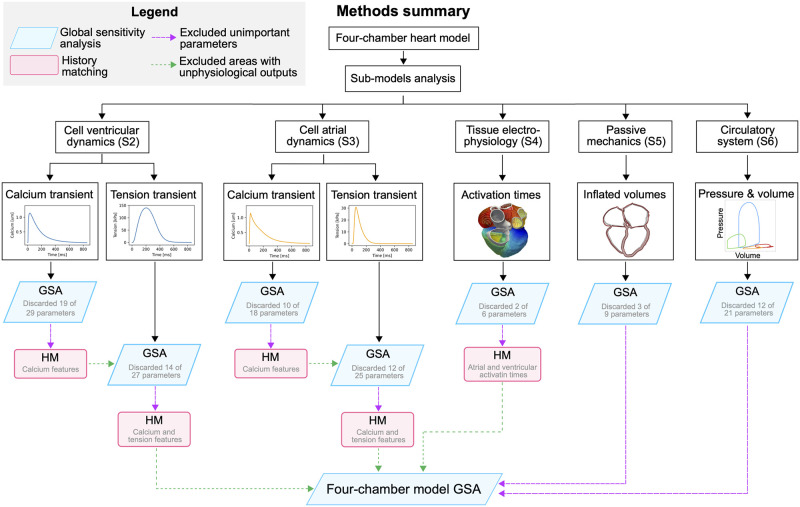
Summary of sub-models analysis. The diagram shows a summary of the analysis performed on the sub-models before performing the GSA on the four-chamber electromechanics model.

Tables 1–3 list all parameters we initially considered. Excluding the values we could derive from the available clinical data, the total number of parameters was 117. This means that we would need to run over 1000 simulations for GPE training, which constitutes a considerable computational cost. To overcome this issue, we used GPEs, GSA and HM methods described above on the different simulator components to exclude unimportant parameters prior to performing the fully coupled simulator runs. Fig 5 summarises the analysis performed on the different sub-models, and below we describe it in detail.

In S2 File, we used GSA and parameter ranking to investigate which parameters affected the calcium transient as, in our simulator, the intracellular calcium is the only coupling variable between the ionic model and the Land model at the cellular level. This process reduced the number of parameters of the ToR-ORd model from 29 to 10. These 10 parameters were then combined with the 17 Land model parameters to identify which ionic and cellular mechanics parameters affected the active tension transient the most, as the cellular active tension drives contraction at the whole organ scale. The GSA on the coupled ToR-ORd-Land model led to a final set of 13 parameters to be considered in the fully coupled simulator. We applied a similar approach to the Courtemanche-Land model (S3 File) to identify 3 out of the 18 Courtemanche parameters and 10 out of the 17 Land model parameters as those affecting cellular atrial active tension the most. For the electrophysiology model at the tissue level, we performed a GSA on activation times only to exclude tissue parameters that did not affect the total atrial and ventricular activation time (S4 File). Due to the potential role fibres play in cardiac activation, we repeated the analysis presented in S4 File with ventricular transmural fibre orientation set to 50°/ − 50° and 70°/ − 70° ventricular fibre direction. There is high uncertainty on the size of the fast conducting regions in the atria and the ventricles (Fig 2). To show that our analysis on the electrophysiology model was independent of this modelling choice, we repeated the analysis with different sizes of the Bachmann bundle area in the atria and of the fast endocardial conduction layer in the ventricles (S10 File). Both analysis showed that the parameters we excluded following the GSA on the tissue electrophysiology model were independent of ventricular fibre direction (S9 File) and the size of the fast conducting regions (S10 File).

We performed a GSA on the passive mechanics model during inflation alone to exclude unimportant stiffness parameters (S5 File). We finally used the CircAdapt ODE model to study the effect of the circulatory system parameters on pressure-volume relationships of all four-chambers (S6 File), and to discard the ones that affected the simulated dynamics the least. The GSA on different simulator components allowed us to systematically reduce the number of free parameters from 117 to 45 prior to running the fully coupled simulator, and to reduce the number of simulations required for GPE training to a more feasible number. The final parameters are represented by the white rows in Table (Tables 1–3).

In addition to running a GSA on each simulator component, we used HM to restrict the important parameters to areas in the parameter space where either the outputs were physiological, or where the mechanics simulations were unlikely to be unstable and diverge, therefore leading to significant waste of computational resources. We constrained the ToR-ORd-Land model so that it provided physiological calcium transients based on the literature [47, 48] (S2 File), and where peak, rest and duration of the isometric tension transient were within ranges of measured values in mammals [49, 50] and allowed the simulations to achieve LV pressure peak and duration that were consistent with clinical data. Similarly, the calcium transient simulated with the Courtemanche model was constrained to be consistent with the literature [47, 48, 51] (S3 File), while the isometric active tension was bound to be physiological [9, 50, 52–59]. We finally applied HM on the electrophysiology Eikonal model alone to achieve physiological total activation times of the atria and the ventricles, as the simulated activation times are independent of the mechanics simulation (S4 File).

In S11 File, we provide a detailed explanation of how we constructed the training samples for the GPEs Briefly, the last HM wave on the ToR-ORd-Land model, the Courtemanche-Land model and the electrophysiology tissue model provided N = 90906, N = 148527 and N = 99723 physiological samples, respectively. The bound for the parameters included in the HM was therefore implicitly defined by the plausible regions of the last HM wave (Tables 1 and 2, second column marked as HM). The bound for all the other parameters is provided in the second column of Tables 1–3. The AV_delay_ was set between 100 and 200 ms, consistently with ranges reported for CRT patients [38]. The range for ventricular stiffness parameters was set to ±50% from values reported in [9], to account for high variability in myocardium stiffness reported in the literature. The bounds for the bulk atrial stiffness *a* were increased based on higher collagen density in the atria compared to the ventricles [41]. Similarly, the range for the scaling parameters for the RV bulk stiffness and the reference tension was chosen to account for higher RV collagen content and lower myocyte density compared to the LV [41]. The length and the stiffness of the aorta were set to ±25% from their default value in CircAdapt [32], while the pulmonary and the systemic resistance factors were set to be between 1.0 and 4.0 to allow for physiological peak in pressure for the ventricles. The pericardium stiffness was bound between 0.5 and 2.0 kPa/mm, consistent with previous studies [4]. The shift on the LV end-diastolic pressure and the RV end-diastolic pressure were set according to measurements in AF patients in sinus rhythm [42]. For these parameters, we performed a Latin hypercube sampling with N = 90906 samples. These were then combined with the plausible regions from the HM on the ToR-ORd-Land model, the Courtemanche-Land model and the electrophysiology tissue model. Having identified the plausible region, we constructed a space filling design in this area on which to run the fully coupled electromechanics simulator and then to train the final GPEs. Because the plausible region had a complicated geometry, we used the function psa_select from the python library diversipy to extract N = 500 uniformly distributed samples.

We trained GPEs to predict the following outputs:

1. LV/RV end-diastolic volume (EDV)
2. LV/RV end-diastolic pressure (EDP)
3. LV/RV end-systolic volume (ESV)
4. LV/RV peak in pressure (p_max_)
5. LV/RV maximum pressure derivative (dp/dt_max_)
6. LV/RV minimum pressure derivative (dp/dt_min_)
7. LA/RA end-diastolic volume prior to atrial contraction (EDV)
8. LA/RA end-systolic volume at the end of atrial contraction (ESV)
9. LA/RA maximum volume during the v-wave (EDV^vwave^)
10. LA/RA peak in pressure during atrial contraction (p_max_).

The trained GPEs were then used to predict the model outputs and to run a GSA on the fully-coupled electromechanics framework, to investigate how the 45 parameters we considered affected whole heart function. During the GSA, we ensured that the GPEs were not evaluated outside their training space by generating a base sequence with N_base_ = 2000 for each simulator sub-component. The GPEs trained to predict total ventricular and atrial activation times (S4 File), atrial and ventricular calcium and active tension transient features (S2 and S3 Files) were evaluated to exclude samples that provided unphysiological activation times, calcium and active tension. The base sequence was resampled for each simulator sub-component with an increasing initial number of samples until >2000 plausible samples were found. A Saltelli sampling was then generated, the samples were screened again with the GPEs for each simulator sub-component to ensure all samples provided physiological outputs. We finally quantified the total effects by using only the samples that were not excluded during the screening process. This allowed us to quantify the effect of simulator parameters on whole heart function while remaining within the GPE emulator training space.

The code to perform the GSA and the HM is provided at https://github.com/MarinaStrocchi/Strocchi_etal_2023_GSA. At this link, we also provide an example on how to train the GPEs, perform a GSA and rank the parameters for the ToRORd ionic model (S2 File).

## Results

**Fig 6:**
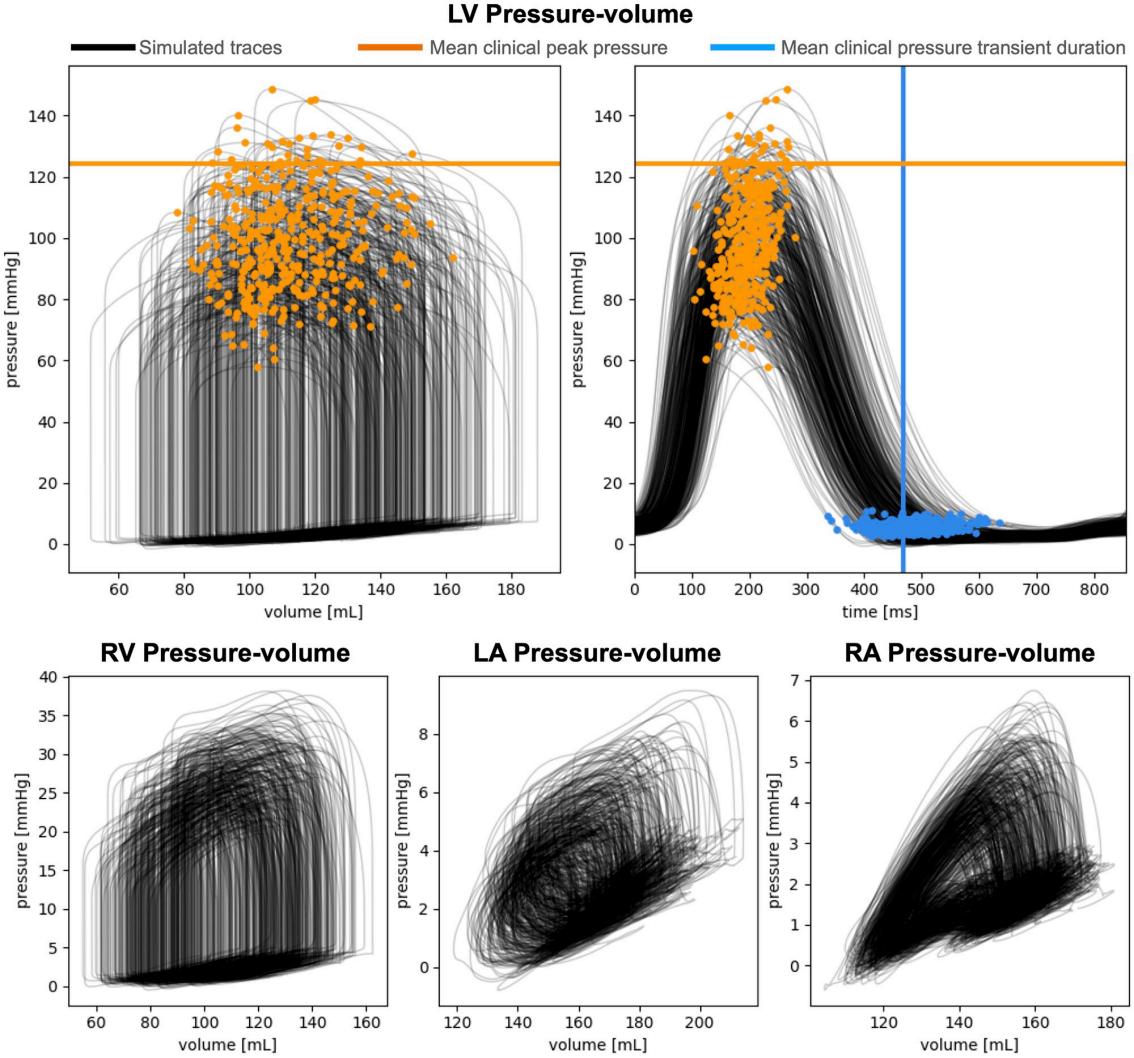
Simulated pressure-volume transients. Top: LV pressure-volume transients and pressure traces simulated with the fully-coupled simulator (black), with the orange and the blue circles representing the resulting LV peak pressure and transient duration. The horizontal and the vertical lines show the clinically measured average LV peak pressure and transient duration, respectively, to show that the simulator achieves physiological values. Bottom: RV, LA and RA simulated pressure-volume transients.

Fig 6 shows the pressure-volume loops for the LV (top), RV, LA and RA (bottom) that were simulated with the four-chamber electromechanics framework. The orange and the blue dots represent the simulated LV peak in pressure and LV pressure transient duration for each sample, respectively. The horizontal and the vertical lines indicate the average measured peak in pressure and pressure transient duration available from clinical data. The simulations achieve the patient’s peak in pressure and result in physiological pressure transient duration, thanks to the HM run on the ToR-ORd-Land model (S2 File). Although some simulations predict a low and unphysiological LV peak in pressure, we included these samples in the GPE training set because these simulations still provided information about the effect of the simulator input parameters and output, while parameter inference was outside the scope of this study. Out of 500 samples, 42 and 51 simulations failed during the unloading and the coupled simulation, respectively. This provided 407 simulations we extracted the pressure and volume features from to train the GPEs. Table 4 shows the mean *R*^2^ and the ISE scores for the GPEs traines for each output. The last column reports the mean scores across all five splits. For all outputs, the mean *R*^2^ and ISE were above 0.76 and 82.0, respectively, indicating that the GPEs provide an accurate estimate for all outputs.

**Table 4:** GPEs performance. Average *R*^2^ score and ISE over the five cross-validation splits, reported for each output.

	Left heart			Right heart		
Meaning	Output	*R* ^2^	ISE	Output	*R* ^2^	ISE
Ventricular end-diastolic volume	EDV_LV_	0.9547	88.93	EDV_RV_	0.9755	91.39
Ventricular end-diastolic pressure	EDP_LV_	0.8982	87.95	EDP_RV_	0.8815	85.73
Ventricular end-systolic volume	ESV_LV_	0.9755	87.95	ESV_RV_	0.9852	88.46
Ventricular peak pressure	${\text{p}}_{\text{LV}}^{\text{max}}$	0.9648	85.50	${\text{p}}_{\text{RV}}^{\text{max}}$	0.9671	88.70
Ventricular max pressure derivative	$\text{dp}/{\text{dt}}_{\text{LV}}^{\text{max}}$	0.9460	90.42	$\text{dp}/{\text{dt}}_{\text{RV}}^{\text{max}}$	0.8250	88.94
Ventricular min pressure derivative	$\text{dp}/{\text{dt}}_{\text{LV}}^{\text{min}}$	0.8901	91.40	$\text{dp}/{\text{dt}}_{\text{RV}}^{\text{min}}$	0.8250	87.71
Atrial end-diastolic volume	EDV_LA_	0.8766	89.42	EDV_RA_	0.8138	83.06
Atrial end-systolic volume	ESV_LA_	0.8938	85.96	ESV_RA_	0.7627	83.52
Atrial peak pressure	${\text{p}}_{\text{LA}}^{\text{max}}$	0.9258	90.16	${\text{p}}_{\text{RA}}^{\text{max}}$	0.8558	84.53
Atrial max volume during v-wave	${\text{EDV}}_{\text{LA}}^{\text{vwave}}$	0.9717	89.67	${\text{EDV}}_{\text{RA}}^{\text{vwave}}$	0.9772	89.43

The 407 model evaluations used for GPE training took an average of 6 h and 20 minutes per job on 512 cores, corresponding to a mean of 3247 core-hours per evaluation and 1321420 core-hours in total for all simulations. For the GSA, we performed 94000 GPE evaluations. Given the computational cost of the whole-heart simulations, it would not be feasible to perform these many model evaluations. Once trained, the GPEs require only ∼1s to estimate the outputs, allowing us to perform the GSA at a treatable computational cost, reduced from an estimate of >305 million core-hours to ∼1.3 million core-hours, due to running the simulations for GPE training. Therefore, thanks to the GPEs, we were able to achieve more than a 300 fold decrease in computational cost.

**Fig 7:**
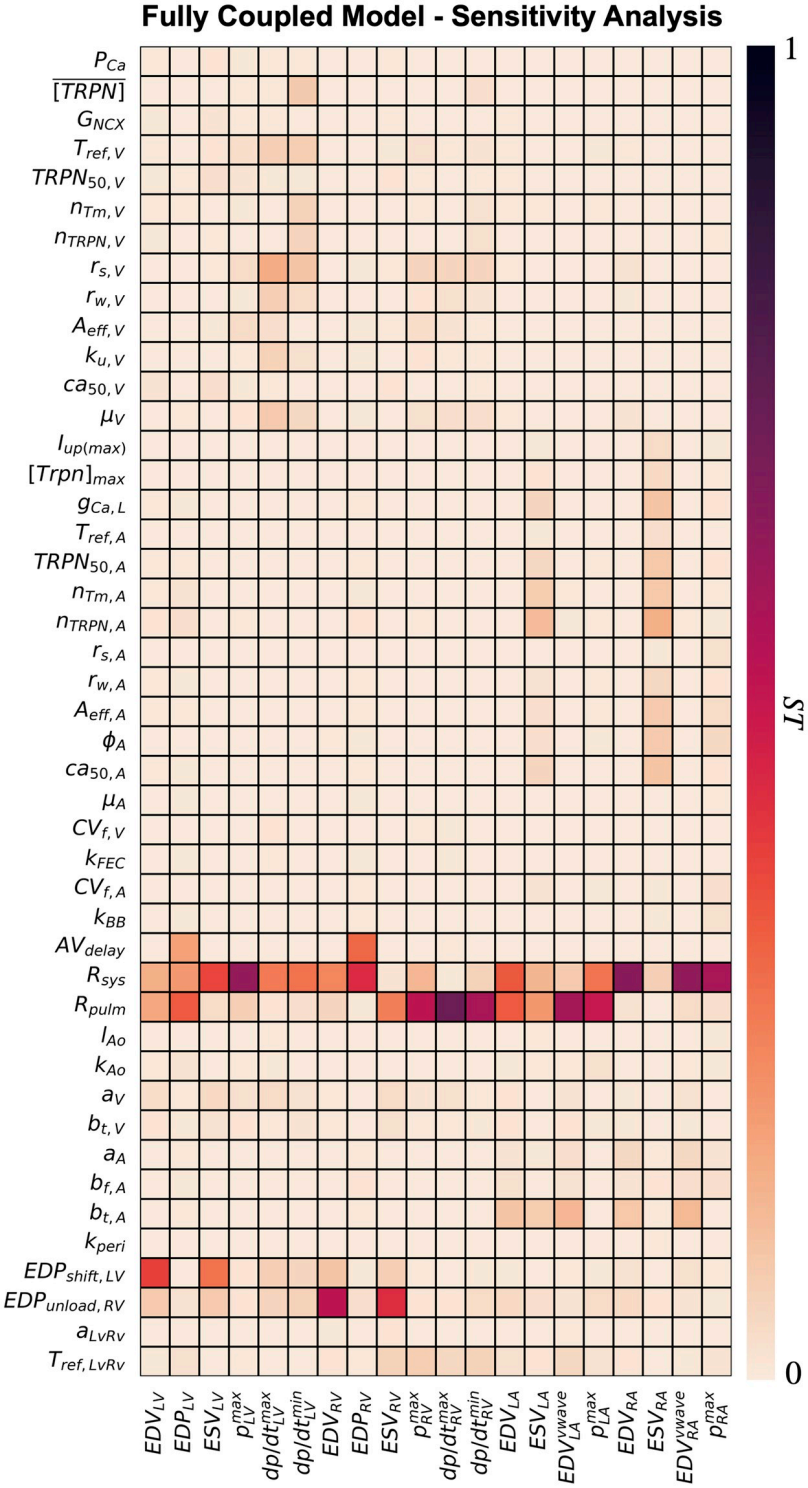
Global sensitivity analysis. Total effects of input simulator parameters (*y*-axis) on model outputs (*x*-axis) normalised between 0 and 1 for each output.

Fig 7 shows the heatmap for the total effect of all input parameters (*y*-axis) over all outputs (*x*-axis), with dark colors showing high interaction between inputs and outputs. Below, we provide a detailed analysis of the sensitivity analysis on the ventricular and atrial diastolic and systolic properties. We also show barplots of the total effects of important parameters for each output, multiplied by the sign of the linear regression coefficients *β*_*i*_ from Eq (4) to give information about positive and negative interactions. In S12 File, we show that the standard deviation of the total effects computed from 1000 different samples of the posterior distribution of the GPEs is small, indicating that GPE uncertainty has negligible effects on the sensitivity indices we reported.

### Ventricular diastolic and atrial systolic properties

**Fig 8:**
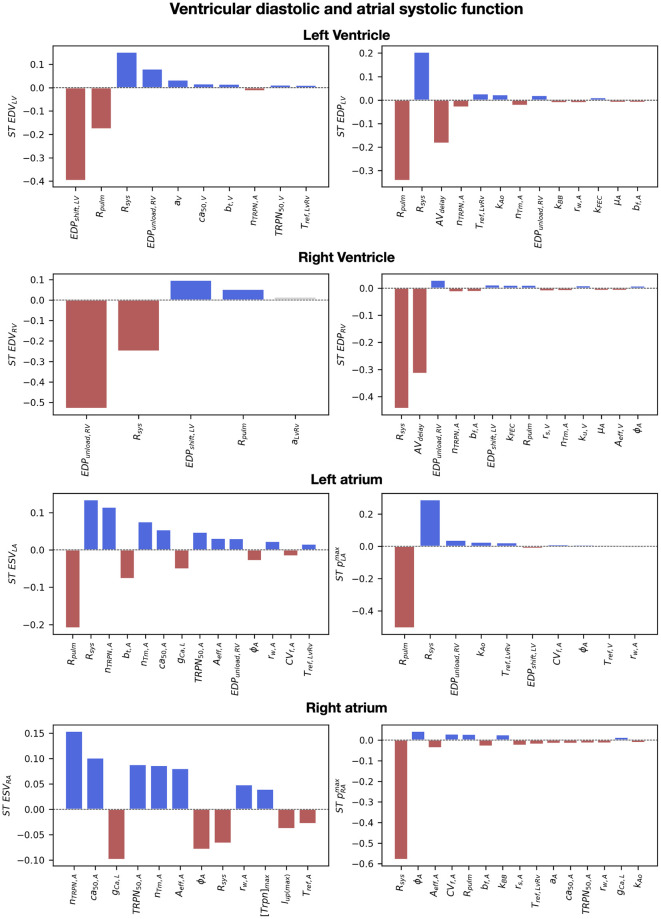
The effect of simulator parameters on ventricular diastole and atrial systole. Barplots of the total order effects of the input parameters, ranked from most to least important, for ventricular EDV, EDP and atrial ESV and peak pressure. The red and blue bars indicate parameters that explain >90% of the output variance, the gray bars indicate parameters that explain up to 95% of variance, while the rest of parameters are not displayed. Red and blue indicate positive and negative interactions, respectively. When this resulted in more than 10 parameters, the number of parameters was limited to 10 or to the number of parameters explaining 90% of output variance.

Fig 8 shows the total order effects on the simulated end-diastolic volume and pressure of the ventricles (first two columns). Blue and red bars represent simulator parameters that are needed to explain 90% of output variation, with positive and negative effects on the output, respectively. Gray bars show parameters needed to explain up to 95% of output variation, while all other parameters are not displayed. The EDV of the ventricles was negatively affected by the unloading pressure of the LV (EDP_LV,shift_) and the RV (EDP_unload,RV_), and by the systemic (R_sys_) and the pulmonary (R_pulm_) resistances. A higher unloading pressure for the ventricles caused higher strains and therefore higher rest tension, that in turn reduced the EDV simulated at the limit cycle. The systemic resistance increased the LV EDV and the LV EDP. This was caused by increased LA ESV (and therefore decreased LA ejection) in response to increased systemic resistance. Similarly, the RV EDV was positively affected by the pulmonary resistance. The parameters that had a negative effect on the LV EDV had a positive effect on the RV EDV and vice-versa. This was caused by the interaction between the ventricles, modulated by the presence of the pericardium and by the intra-ventricular septum. When the LV EDV is bigger, the septum bulges towards the RV, leading to smaller RV EDV and therefore opposite effects of simulator parameters on the LV EDV and the RV EDV. Finally, passive material properties of the ventricles (*a*_*V*_ and *b*_*t*,*V*_) positively affected the EDV of the LV. Although this might be counter-intuitive, decreased stiffness causes smaller unloaded volumes and higher initial strains at the beginning of the simulations, leading to higher ventricular rest tension and therefore decreased LV EDV.

**Fig 9:**
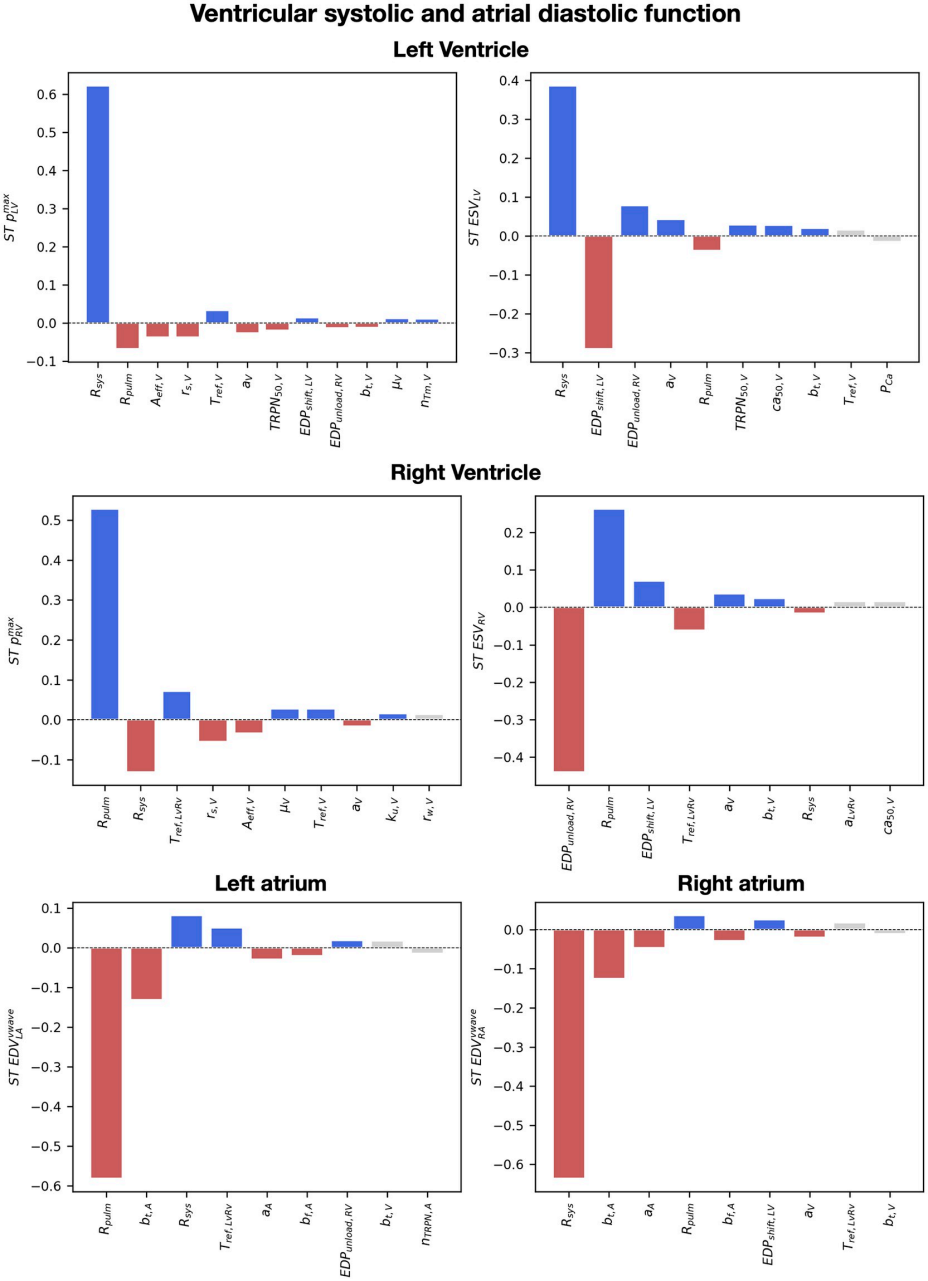
The effect of simulator parameters on ventricular systole and atrial diastole. Barplots of the total order effects of the input parameters, ranked from most to least important, for ventricular peak pressure, ESV and atrial maximum volume during the v-wave. The red and blue bars indicate parameters that explain >90% of the output variance, the gray bars indicate parameters that explain up to 95% of variance, while the rest of parameters are not displayed. Red and blue indicate positive and negative interactions, respectively. When this resulted in more than 10 parameters, the number of parameters was limited to 10 or to the number of parameters explaining 90% of output variance.

The systemic and pulmonary peripheral resistances also affected atrial systolic peak in pressure (Fig 8, third and fourth columns). The LA peak in pressure was decreased by higher pulmonary resistance, caused by smaller LA preload (negative interaction between LA EDV in Fig 9). Similarly, increased systemic resistance led to smaller RA peak in pressure. The systemic resistance had also a significant positive impact on the LA peak in pressure. The RA pressure was also driven up by the pulmonary resistance, but with smaller interaction compared to the LA pressure and the systemic resistance. The unloading pressure (EDP_unload,RV_) and the reference tension scaling factor (T_ref,LvRv_) of the RV had minor positive effects on the LA peak in pressure. In addition to the systemic resistance, the RA peak in pressure was affected by cellular ionic and active tension parameters for velocity dependence (*ϕ*_*A*_, *A*_*eff*,*A*_), cross-bridge kinetics (*r*_*s*,*A*_, *r*_*w*,*A*_) and calcium handling (*ca*_50,*A*_, *TRPN*_50,*A*_ and *g*_*CaL*_). Atrial stiffness (*a*_*A*_ and *b*_*f*,*A*_) and atrial conduction velocities (*CV*_*f*,*A*_ and *k*_*BB*_) also played a minor but significant role in RA peak pressure, with stiffer and slower myocardium causing lower RA pressure values.

The unloading pressures of the LV and RV affected the EDV of the ventricles the most, as they determined the end-diastolic strains and therefore the ventricular rest active tension. The systemic and pulmonary resistances had significant effects on the EDV as well, due to their effect on atrial ejection, while the AV delay mostly affected the EDP. Parameters for atrial active tension timing, CV in the ventricular endocardium and in the Bachmann bundle showed minor effects on the EDP of the ventricles, due to their effect on timing of RV initial contraction and atrial contraction rise and decay, respectively. Finally, the effect of parameters on the LV EDV was opposite to the effect on the RV EDV due to ventricular interaction modulated by the pericardium.

### Ventricular systolic properties

The systemic and the pulmonary resistances affected the peak in pressure of the LV and the RV the most (Fig 9, top). Increased systemic and pulmonary resistances increase the afterload of the LV and the RV, therefore leading to higher LV and RV peak in pressure. The negative effect of the pulmonary resistance on the LV EDV caused smaller ventricular load, leading to a negative effect on the LV peak in pressure. Similarly, the RV peak in pressure was negatively affected by the systemic resistance. Higher bulk passive stiffness of the ventricles (*a*_*V*_) decreased the peak in pressure and increased ventricular ESV of both ventricles due to less significant deformation during active contraction. Parameters for ventricular cellular active tension also had a significant effect on the peak in pressure of the ventricles. Degree of velocity dependence (*A*_*eff*,*V*_), cross-bridges kinetics parameter (*r*_*s*,*V*_, *TRPN*_50_ and *μ*_*V*_) and the reference active tension (*T*_*ref*,*V*_) had significant effects on the peak in pressure, as they affected the timing and extent of cellular peak in cellular active tension, as shown in S2 File. Furthermore, the RV peak in pressure was positively affected by the LV-RV scaling of the reference tension *T*_*ref*,*LvRv*_.

LV ESV was positively affected by the systemic resistance and the unloading pressure of the RV. On the other hand, the unloading pressure of the LV negatively impacted LV ESV. The RV ESV was similarly affected by the unloading pressure of the RV and the LV, and by the pulmonary resistance. Increased peripheral resistance downstream to the LV and the RV led to higher ventricular afteload, therefore causing higher ESV. The passive stiffness of the ventricles *a*_*V*_ and *b*_*t*,*V*_ had minor positive effects on the ESV of both ventricles, therefore leading to smaller LV and RV ejection.

**Fig 10:**
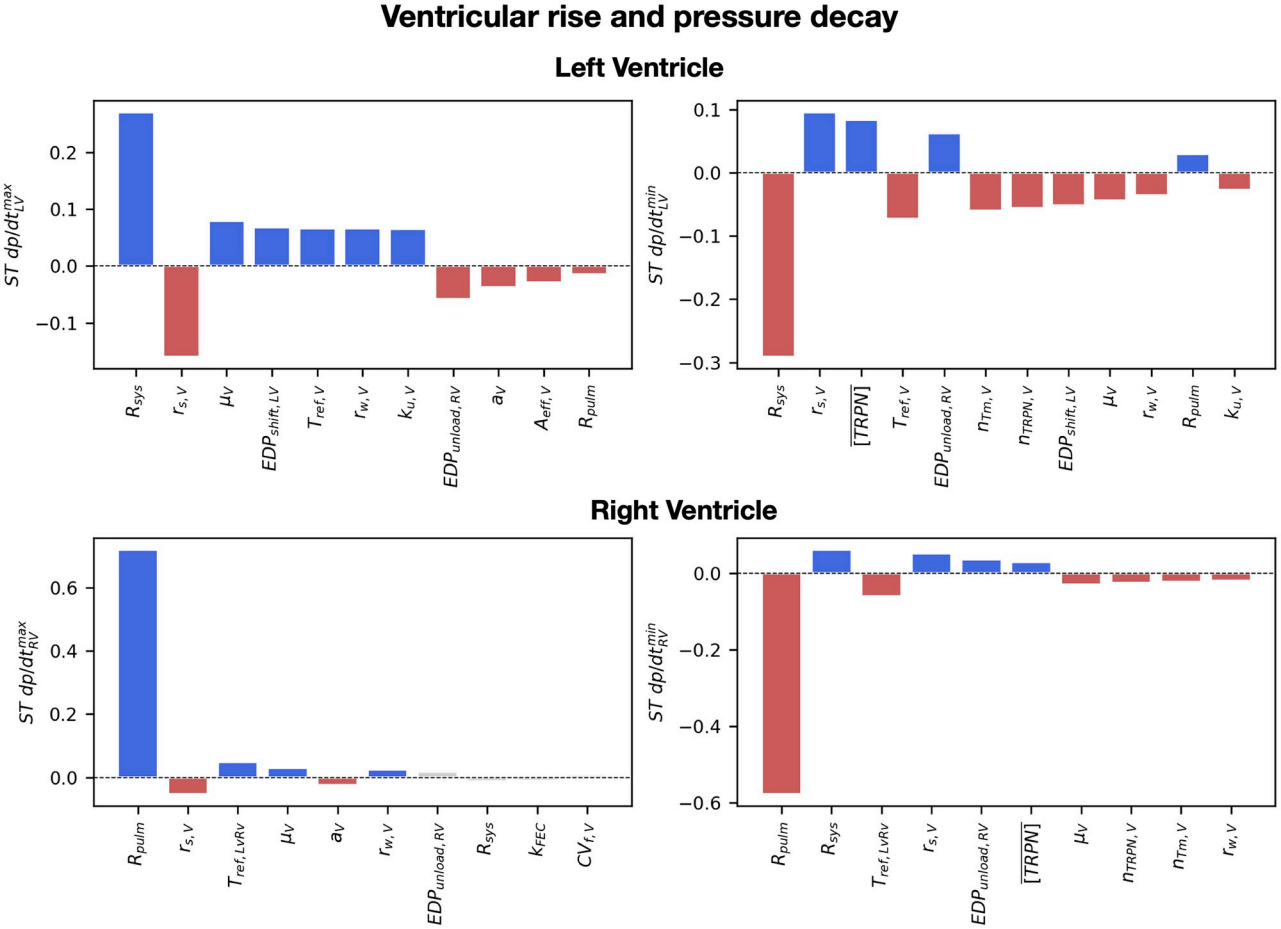
The effect of simulator parameters on ventricular pressure rise and decay rate. Barplots of the total order effects of the input parameters, ranked from most to least important, for ventricular maximum and minimum derivatives. The red and blue bars indicate parameters that explain >90% of the output variance, the gray bars indicate parameters that explain up to 95% of variance, while the rest of parameters are not displayed. Red and blue indicate positive and negative interactions, respectively. When this resulted in more than 10 parameters, the number of parameters was limited to 10 or to the number of parameters explaining 90% of output variance.

Ventricular pressure rise and decay rate were strongly affected by the systemic and the pulmonary resistances, but also by ventricular active tension parameters (Fig 10). Increased systemic resistance led to higher LV peak in pressure and therefore faster rise and decay in LV pressure. Similarly, RV rise and decay in pressure was strongly affected by the pulmonary resistance due to its effect on the RV peak in pressure. Parameters for ventricular cross-bridge kinetics (*r*_*s*,*V*_, *r*_*w*,*V*_, *k*_*u*,*V*_) affected both LV and RV maximum pressure derivative due to their effect on cellular active tension rising time and maximum tension derivative, as we showed in S2 File. Increased ventricular passive stiffness (*a*_*V*_) caused slower LV and RV pressure rise in both ventricles. In addition, LV pressure rise was impacted by the unloading pressures of the LV and the RV due to their effect on end-diastolic strains, by the rate of transition from blocked to unblocked cross-bridge binding sites (*k*_*u*,*V*_) and by ventricular velocity dependence (*A*_*eff*,*V*_). The reference tension of the ventricles (*T*_*ref*_) and the scaling for the RV reference tension (*T*_*ref*,*LvRv*_) had an effect on the maximum pressure derivative of the LV and the RV, respectively, due to their effect on cellular active tension peak and timing. Apart from velocity dependence and ventricular stiffness, all important parameters for the LV and RV rise in pressure also impacted the LV and RV minimum pressure derivative. In addition, ventricular minimum pressure derivative was affected by the maximum troponin concentration ($\bar{\left[TRPN\right]}$) of the ToR-ORd model, due to its effect on calcium relaxation (S2 File), and by *n*_*TRPN*,*V*_ and *n*_*Tm*,*V*_, as they affected ventricular active tension decay at the cellular level.

The systolic dynamics of the ventricles are strictly related to atrial filling dynamics due to venous return. RV output was decreased by high pulmonary resistance. This in turn caused smaller LA maximum volume during the v-wave, e.g. smaller LA venous return. Similarly, due to decreased LV output, systemic venous return to the RA got smaller when the systemic resistance was high. Atrial stiffness parameters *a*_*A*_, *b*_*f*,*A*_ and *b*_*t*,*A*_ negatively affected venous return to the atria due to decreased atrial compliance. Finally, the unloading pressure and the passive stiffness of the ventricles *a*_*V*_ had minor effects on venous return to the atria, due to their effect on ventricular output.

The systemic and the pulmonary resistances had the highest impact on ventricular systolic dynamics because they determine ventricular afterload. However, ventricular calcium and active tension parameters had significant impact on systolic function, in particular on pressure rise and decay. Atrial filling during the v-wave was affected by the peripheral resistances, but also by atrial stiffness parameters.

## Discussion

In this study, we presented the first GSA on a four-chamber electromechanics simulator coupled with the circulatory system and accounting for atrial and ventricular contraction triggered by the calcium transient, as well as the effect of the pericardium. GPEs and GSA were used to identify important parameters for tissue electrophysiology, passive mechanics, the circulatory system and cellular dynamics. This analysis allowed us to reduce the number of parameters from 117 to 45. HM was then used to restrict the parameters for the cellular electromechanics simulator and tissue electrophysiology to areas in the parameter space where calcium, tension and total ventricular and atrial activation times were physiological. This provided 407 successful four-chamber electromechanics simulations to train the GPEs to predict features for whole organ function, and run a GSA on the fully coupled simulator. We showed that our analysis allows us to link cellular dynamics for calcium and tension to whole organ function. This framework can be used to improve our understanding of cardiac physiology and pathophysiology, and ultimately to provide novel insights into patient-specific treatment planning.

### The effect of increased vascular resistance on heart function

Our results show that systemic and pulmonary peripheral resistance have significant effects on ventricular systolic function. In agreement with our analysis, Linde et al. [60] measured elevated systemic vascular resistance in hypertensive patients, contributing to elevated systolic pressure. Furthermore, drug studies reported on the efficacy of beta-blockers with vasodilatory activity for hypertension treatment, because the drug-induced decrease in the systemic resistance drives the LV systolic pressure down [61]. Increased systemic resistance was not only related to increased LV pressures, but also to decreased LV ejection in patients without known history of cardiovascular diseases [62], consistent with our analysis. Similarly, pulmonary hypertension patients have been reported to have increased pulmonary vascular resistance [63], therefore leading to increased RV pressure and reduced RV SV compared to normal subjects.

Not only systolic but also diastolic ventricular properties are affected by peripheral resistances. Harshaw et al. [64] explored the effect of a drug-induced decrease in systemic resistance in patients with mitral valve dysfunctions, showing a positive relationship between LV EDP and peripheral resistance, similar to our analysis. Further, patients with cirrhosis were reported to have increased left heart volumes and decreased right heart volumes due to reduced systemic resistance compared to controls, in agreement with our study [65]. RV EDV is elevated in pulmonary hypertensive patients [63] due to increased pulmonary vascular resistance, and was shown to decrease as a consequence of lower-body suction, aimed at reducing RV afterload through reduced peripheral resistance [66]. Several studies report on inverse effects of left and right heart volumes in response to increased resistance, with LV EDV decreasing with higher pulmonary resistance and RV EDV decreasing with higher systemic resistance [65–67]. This has been attributed to diastolic ventricular interaction exerted by the pericardium [66], which causes an inverse relation between the LV EDV and the RV EDV, consistent with our results.

The systemic and the pulmonary resistances connect the left and the right sides of the hearts. Therefore, changes in the pulmonary and the systemic resistances induce alterations in the atria as well as in the ventricles. We have found a positive and negative linear correlation between the LA volumes and the systemic and the pulmonary resistances, respectively. Consistently with these findings, Marston et al. [68] reported a negative relation between pulmonary vascular resistance and LA volumes in patients with pulmonary hypertension. Furthermore, changes in pulmonary resistance following blood clots removal led to increased LA volumes and LV EDV. In addition, also consistent with our findings, in hypertensive patients, where the systemic resistance is higher than in normal subjects, the LA was dilated [69].

Increased or decreased systemic and pulmonary vascular resistances can occur in a wide range of pathologies, such as hypertension or cirrhosis. Vascular resistance has important effects on both the left and the right sides of the heart due to diastolic ventricular interaction mediated by the pericardium, and due to the interaction between atria and ventricles, mediated by the circulatory system. By coupling the three-dimensional mechanics framework with a closed-loop model for blood circulation and by including the effect of the pericardium on the heart, our simulator is able to account for these complex interactions and simulate how all four chambers respond to altered peripheral resistances.

### Links between cell and whole organ function

Reduced contractility and consequent decreased atrial ejection during atrial contraction is a well recognized consequence of AF, even after cardioversion [70]. However, over the years, several studies have proposed different explanations for this mechanism. Active tension measurements in myocytes isolated from the right atrial appendage in AF patients have shown reduction of cellular contractile force compared to control, mainly caused by reduced the L-type calcium current [71, 72]. In agreement with these findings, our results showed increased atrial systolic volumes in response to lower L-type calcium ion channel conductance. Conduction slowing caused by loss of cell-to-cell coupling and/or by the presence of fibrosis might also contribute to depressed atrial contractility in AF patients [70, 73], consistent with the correlation between atrial conduction velocity and atrial end-systolic volume we found in our analysis. Finally, increased atrial stiffness, reported in AF patients [70] but also in other cardiac diseases, including hypertrophic cardiomyopathy [74], leads to impaired atrial compliance and smaller atrial end-diastolic volume, in agreement with our findings.

Efficiency of contraction can be measured using pressure biomarkers, such as peak in pressure or maximum pressure derivative, while pressure decay provides an indication of velocity of relaxation. Pressure relaxation was reported to be slower in diastolic HF patients [75], while systolic HF causes slower pressure rise due to sub-optimal contraction. Using our analysis, we were able to link these whole organ biomarkers to cellular function. We have shown that ventricular peak in pressure and pressure derivatives were significantly affected by tension velocity dependence, reference tension and cross-bridge kinetics parameters. Cellular measurements on isolated failing myocytes in animals and human tissue preparations showed that tension velocity dependence [76], cross-bridge cycling rate [77] and force development [78] were altered in failing subjects. Furthermore, diseased myocytes were reported to have slower calcium transient relaxation [48, 78], which, according to our analysis, might lead to slower pressure relaxation at the whole organ level.

Cardiac pathophysiology is complex, and can span across different length scales, from subcellular processes involving ionic channel remodelling and cross-bridge kinetics up to changes at the whole organ level. Although the parameter ranges we chose do not necessarily capture pathological variability caused by the cardiac diseases mentioned above, we have shown that our analysis can be used to link cellular function to biomarkers measured at the whole organ level. This demonstrates that multi-scale electromechanics models have the potential to improve our understanding of the pathophysology of a wide range of atrial and/or ventricular disorders.

### Limitations

Although our study constitutes the first GSA on a four-chamber electromechanics model, accounting for multi-scale processes spanning from the sub-cellular to the whole organ level, it has some limitations.

Due to model complexity and to reduce computational costs, we did not run a fully converged Newton solution by constraining the maximum number of Newton iterations to 2. This introduced small errors in the simulated pressure and volume features below 3%, but it also allowed to speed up the simulations by up to 3 times. This reduced the computational cost of our GSA, making it possible to run more than 400 simulations and train GPEs that could accurately provide output predictions as function of 45 model parameters.

Our four-chamber electromechanics framework does not account for mechano-electrical feedback, assuming that mechanical deformation does not affect cellular and tissue electrophysiology. However, there are known mechanisms in which stretch induces changes in the electrical activity of the heart through, for instance, stretch-activated ion channels [79]. These mechanisms might play a role in a some pathologies, such as arrhythmogenesis initiation [80]. Therefore, depending on the application, additional feedback mechanisms might need to be included in the model, to provide a more accurate representation of the patient’s heart.

In our analysis, we only performed four-chamber simulations on one patient-specific anatomy due to their extensive computational cost. While the anatomy might impact the sensitivity analysis at the whole organ level, the analysis ran on the circulatory model, and the ventricular and atrial ionic and contraction models is independent of the geometry and therefore the conclusions for these models remain valid for other patients. In future, the anatomy could be accounted for in the GSA by performing a principal component analysis on a cohort of geometries and adding the principal vectors as additional parameters in the GSA, as in Rodero et al. [81]. In our analysis, we also fixed the fibre direction of atrial and ventricular myocardium, despite the high uncertainty in fibre direction reported in the literature. In S9 File, we showed that ventricular myofibre orientation has limited effects on the tissue electrophysiology GSA, passive mechanics and electromechanics model outputs, consistent with previous computational studies [82, 83]. However, we did not repeat the whole study with different fibre orientation, and we did not investigate the effect atrial myofibres as their arrangement is more complex and less well-established than that of ventricular fibre orientation. When more measurements of atrial myofibre orientation are available, it will be important to establish their effect on cardiac function.

To retrieve the stress-free configuration of the heart, we unloaded the ventricles but not the atria. This prevented excessive deformation in the atria during the unloading procedure, which makes the mechanics simulations more likely to diverge. Furthermore, we discarded diastolic residual active tension, even though this might play a role in relaxation dynamics. In future, residual active tension and atrial pressures could be accounted for in the unloading procedure [84], in order to have a more accurate estimation of the stress-free geometry.

In S2 and S3 Files, we performed GSA and HM on the ToR-ORd and Courtemanche models alone and coupled with the Land model to identify unimportant parameters for atrial and ventricular calcium and the active tension transients. The cell simulations used to train the GPEs in these instances were run with a basic cycle length of 1000 ms, corresponding to 1 Hz pacing, as opposed to using the basic cycle length derived from the clinical data. This was done to match the literature data we used as target values for the calcium transient features. Additionally, this makes the findings at the cellular level more general and applicable to other patients.

The GSA results we presented remain true within the parameter bounds and model outputs we included in the analysis. We only considered a subset of the 117 parameters that, within an expected physiological variability, had the largest impact on specific model outputs. Extreme changes in parameters, for example pathological or pharmacological inhibition of ion channels outside our assumed physiological variability, may result in other parameters outside of the 45 identified as being important in our analysis. Nevertheless, additional parameters could be re-introduced after the fitting process to investigate a particular cardiac pathology/treatment of interest.

In this study, we performed a GSA on five sub-models (S1–S5 Files) to exclude parameters that did not affect sub-model outputs, under the assumption that these parameters would also be unimportant for whole-organ biomarkers. This could be done thanks to our knowledge of how the different sub-models are coupled within the four-chamber electromechanics framework. While this approach might fail to capture interactions between the parameters we excluded from the sub-models, this also allowed us to reduce the computational cost required to generate the simulations for emulators training and therefore the GSA. Nevertheless, since the parameters we excluded had small, if any, effects on sub-model outputs, we expect the combined effects between unimportant parameters of different sub-models to be small as well. Furthermore, in our GSA and HM, we did not consider any potential correlation between different outputs. Training a GPE for each output separately provided us the flexibility to fit the hyperparameters for each GPE independently. This could have been accounted for by training a multi-output GPE instead [85], where the GPE degrees of freedom are estimated for all outputs at the same time rather than independently. Furthermore, HM with a multi-variate GPE would have potentially helped in ruling out more implausible areas of the parameter space.

The GSA we presented does not account for the uncertainty of the multi-scale model, since quantifying model error is a more challenging task than learning the parameters of the model itself. In [86], Kennedy and O’Hagan showed how to account for model discrepancy, also referred to as model inadequacy, by adding it as an additional source of uncertainty. While the Kennedy and O’Hagan approach is simple and easy to apply, challenges arise during model parameter inference, since the observed data may be attributed either to the model itself or the model error, or both, meaning that the model parameters can no longer be consistently identified [87]. An elegant resolution involving orthogonal Gaussian processes was proposed in [88]. This approach is applicable on a linear model but, for non-linear models like the multi-scale four-chamber model presented in this study, retaining consistency of parameter estimates while accounting for model error remains an open research problem. Furthermore, although we sampled the GPEs posterior distribution and computed the average total effects from these samples, the sensitivity indices in our GSA were computed without directly accounting for GPE uncertainty. In future studies, the uncertainty of the GPE prediction could be accounted for by using alternative approximations of the sensitivity indices that directly include GPE standard deviation as well as the expected value.

Despite its limitation, our GSA allows us to link cellular processes with whole organ function, and has the potential to provide novel insights into patient pathophysiology and response to treatment that could never be tested in-vivo.

## Conclusion

Our four-chamber electromechanics simulator is able to account for LV-RV and AV mechanical interaction, modulated by the presence of the pericardium and the coupling with a closed-loop model for the circulatory system. The GSA we presented allowed us to make considerations about how cellular dynamics translate into altered whole organ function. Thanks to the wide range of dynamics we can simulate, this analysis could potentially be applied to investigate a wide range of pathologies affecting the atria and the ventricles, and the consequences these have on all other chambers.

## Supporting information

S1 File Numerical tests. We compared the model solution obtained with our numerical settings and a fully converged solution.
(PDF)

S2 File ToR-ORd-Land model sensitivity analysis and history matching. We trained GPEs to predict calcium and active tension transient features simulated by the ToR-ORd model coupled with the Land contraction model, and used them to run a GSA to identify important parameters for these dynamics. Then, we used HM to isolate areas in the parameter space where the ventricular calcium and tension were physiological.
(PDF)

S3 File Courtemanche-Land model sensitivity analysis and history matching. We trained GPEs to predict calcium and active tension transient features simulated by the Courtemanche model coupled with the Land contraction model, and used them to run a GSA to identify important parameters for these dynamics. Then, we used HM to isolate areas in the parameter space where the atrial calcium and tension were physiological.
(PDF)

S4 File Tissue electrophysiology sensitivity analysis and history matching. We trained GPEs to predict the total atrial and ventricular activation times, and used them to run a GSA to identify important conduction parameters for simulated activation times. Then, we used HM to isolate areas in the parameter space where the total ventricular and atrial activation times were physiological.
(PDF)

S5 File Passive mechanics sensitivity analysis. We trained GPEs to predict the maximum volumes for the four chambers and ventricular and atrial fibres strains during a passive inflation, and used them to run a GSA to identify important stiffness parameters.
(PDF)

S6 File CircAdapt sensitivity analysis. We trained GPEs to predict a wide range of pressure and volume output features for all four chambers simulated with CircAdapt, and used them to run a GSA to identify important circulatory parameters for pressure and volume dynamics.
(PDF)

S7 File Saltelli sampling construction. Detailed explanation of how we constructed the Saltelli samples for the GSA.
(PDF)

S8 File Gaussian processes emulators kernel comparison. We investigated how the choice of kernel affected GPE accuracy and GSA. The GPE training and GSA performed for the ToR-ORd model (S2 File) were repeated with a Matérn rather than an exponentiated quadratic kernel and the results between the two cases were compared.
(PDF)

S9 File The effect of ventricular fibre orientation. We performed the GSA on the electrophysiology tissue model with two different ventricular fibre orientation to investigate the effect of myofibre arrengement on electrophysiology simulations and GSA. We also repeated a passive inflation and a four-chamber electromechanics simulation to quantify the effect of ventricular fibres on mechanics model outputs.
(PDF)

S10 File The effect of fast conducting regions size on the electrophysiology model. We performed the GSA on the electrophysiology tissue model altering the size of the fast endocardial conducting layer of the ventricles and the Bachmann bundle.
(PDF)

S11 File Non-implausible region extraction. Schematic of how the non-implausible areas from the HM of the sub-models were used to construct the training samples for the GPEs at the whole organ scale.
(PDF)

S12 File The effect of emulators uncertainty on sensitivity indices. We show the mean and standard deviation of the total effects computed for the whole organ sensitivity analysis obtained when sampling the posterior distribution of the emulators.
(PDF)
