# Supplementary material for: Cell to whole organ global sensitivity analysis on a four-chamber heart electromechanics model using Gaussian processes emulators
Source: PLoS Comput Biol. 2023 Jun 26;19(6):e1011257. doi: 10.1371/journal.pcbi.1011257 (PMC10328347; doi:10.1371/journal.pcbi.1011257)
Supplement: S1 File — We compared the model solution obtained with our numerical settings and a fully converged solution. (PDF) [file pcbi.1011257.s001.pdf]

Four-chamber electromechanics framework: numerical tests

To show that our numerical settings have limited impact on the simulated dynamics, we compared the results for one of the simulations with the following settings:

1. **Baseline:** tolerance for the solution of the linearised system set to 10^{-4} and last two beats with the maximum number of Newton iterations set to 2
2. **Decreased linearised system tolerance:** tolerance for the solution of the linearised system set to 10^{-8} and last two beats with the maximum number of Newton iterations set to 2
3. **Fully converged Newton:** tolerance for the solution of the linearised system set to 10^{-4} and last two beats with the maximum number of Newton iterations set to 10 and convergence tolerance set to 10^{-3}.

Table 1 and Fig 1 show the results of the numerical tests described above. Compared to the baseline numerical settings, both numerical tests led to limited impact on the features extracted from the simulated pressure and volume traces for all four chambers, with errors always below 3%. Having a fully converged Newton solution for the last two beats has a detectable effect on the simulated pressure-volume loops (Fig 1, right). These small differences are within the GPEs uncertainty, and allowed us to speed up the simulation of up to three times (Table 1, highlighted row). We therefore used these numerical settings for all other simulations.

Table 1. Numerical test results.

	**Baseline**	**Linearised system tol**	**Fully converged Newton**
**Simulation time**	3h:29min	6h:47min	9h:27min
**Model output**		**Output value (% error vs baseline)**	**Output value (% error vs baseline)**
**Left ventricle**			
EDV [mL]	150.883	150.893 (0.0%)	150.684 (0.1%)
EDP [mmHg]	5.63	5.656 (0.5%)	5.639 (0.2%)
ESV [mL]	108.275	108.275 (0.0%)	109.277 (0.9%)
p_{max} [mmHg]	113.794	113.79 (0.0%)	111.341 (2.2%)
dp/dt_{max} [mmHg/s]	1502.0	1492.0 (0.7%)	1461 (2.7%)
dp/dt_{min} [mmHg/s]	-671.0	-676.0 (0.7%)	-673.0 (0.3%)
**Right ventricle**			
EDV [mL]	118.307	118.307 (0.0%)	117.759 (0.5%)
EDP [mmHg]	3.07	3.069 (0.0%)	3.038 (1%)
ESV [mL]	79.445	79.445 (0.0%)	79.929 (0.6%)
p_{max} [mmHg]	25.476	25.475 (0.0%)	25.116 (1.4%)
dp/dt_{max} [mmHg/s]	347	352.0 (1.4%)	340.0 (2%)
dp/dt_{min} [mmHg/s]	-139.0	-135.0 (2.9%)	-136.0 (2.2%)
**Left atrium**			
EDV [mL]	178.729	178.729 (0.0%)	179.941 (0.7%)
ESV [mL]	144.842	144.842 (0.0%)	146.007 (0.8%)
V_{max} v-wave [mL]	182.703	182.703 (0.0%)	182.837 (0.1%)
p_{max} [mmHg]	5.311	5.312 (0.0%)	5.203 (2.0%)
**Right atrium**			
EDV [mL]	151.703	151.702 (0.0%)	151.551 (0.1%)
ESV [mL]	123.093	123.093 (0.0%)	123.857 (0.6%)
V_{max} v-wave [mL]	157.861	157.861 (0.0%)	157.553 (0.2%)
p_{max} [mmHg]	3.316	3.316 (0.0%)	3.225 (2.7%)

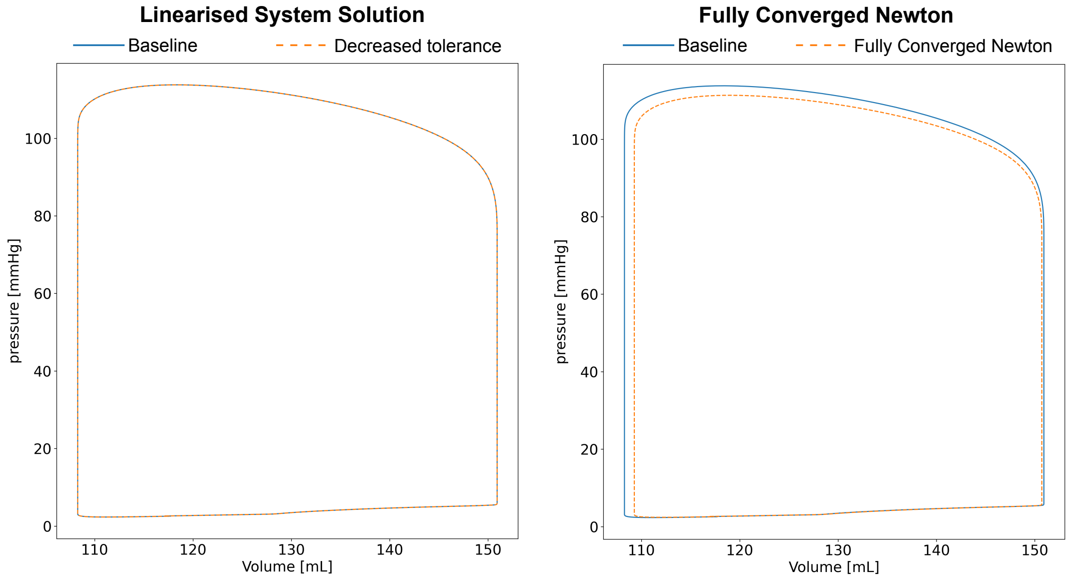

Fig 1. Numerical tests results. Left ventricular pressure-volume loop simulated during the last beat with baseline numerical settings (blue solid line), with decreased tolerance for the linearised system solution (dashed orange line, left) and with fully converged Newton (dashed orange line, right).
