# Supplementary material for: Cell to whole organ global sensitivity analysis on a four-chamber heart electromechanics model using Gaussian processes emulators
Source: PLoS Comput Biol. 2023 Jun 26;19(6):e1011257. doi: 10.1371/journal.pcbi.1011257 (PMC10328347; doi:10.1371/journal.pcbi.1011257)
Supplement: S2 File — We trained GPEs to predict calcium and active tension transient features simulated by the ToR-ORd model coupled with the Land contraction model, and used them to run a GSA to identify important parameters for these dynamics. Then, we used HM to isolate areas in the parameter space where the ventricular calcium and tension were physiological. (PDF) [file pcbi.1011257.s002.pdf]

ToR-ORd and Land model sensitivity analysis and history matching

We used Gaussian process emulators (GPEs) and global sensitivity analysis (GSA) to identify the important parameters of the ToR-ORd model coupled with the Land model. First, we ran the analysis on the ToR-ORd model alone to identify important parameters for the calcium transient features. We then used Bayesian history matching (HM) to isolate the parameter areas where the calcium transient was physiological. The ToR-ORd model was then coupled with the Land model to run a GSA to investigate which ionic model and active tension parameters affected the active tension transient features. Finally, HM was used to find the areas of the parameter space where the active tension was physiological and allowed to achieve an LV peak in pressure that was comparable to physiological values available from the clinical data.

ToR-ORd model

Global sensitivity analysis

Table 1 shows the parameters we considered in the analysis on the ToR-ORd model. We included the conductances for all ionic currents: fast and slow sodium currents G_{Na} and G_{NaL}, transient outward potassium G_{to}, rapid and slow delayed potassium rectifier currents G_{Kr} and G_{Ks}, inward potassium rectifier current G_{K1}, L-type calcium current P_{Ca}, sodium-calcium exchanger G_{NCX}, sodium-potassium pump current G_{NaK}, calcium pump current G_{Ca}, calcium-sensitive chloride current G_{ClCa}, all background currents G_{Nab}, P_{Cab}, G_{Kb}, G_{Clb}; and the parameters representing calcium handling: calcium release ($\overline{J}_{rel}$) and uptake ($\overline{J}_{up}$) in the sarcoplasmic reticulum, fraction of sodium-calcium exchangers ($\overline{I}_{NaCa,SS}$) and L-type calcium channels ($\overline{I}_{CaL,SS}$) located in the subspace, calmodulin-kinase kinetics parameters (α_{CaMK}, β_{CaMK}, $CaMK_{\text{o}}$), calcium buffer concentrations ($\overline{[\text{CMDN}]}$, $\overline{[\text{TRPN}]}$, $\overline{[\text{BSL}]}$, $\overline{[\text{BSR}]}$ and $\overline{[\text{CSQN}]}$) and calcium diffusion rates ($\tau_{\text{diff,Ca}}$ and τ_{tr}). For details about the ToR-ORd model, all symbols and default values, the reader is referred to the original publications [1–3]. For GPE training and GSA, the range for all parameters was set to ±25% from the default value. The parameter space was sampled with a latin hypercube design with N=2175 points. For each sample, we ran the ToR-ORd model for 100 beats and a cycle length of 1000 ms. The last beat was used to compute the following calcium transient features:

1. diastolic calcium level (Ca_{diast})
2. calcium transient amplitude (Ca_{ampl})
3. time to peak (TTP)
4. time to reach 90% of calcium transient decay (RT90).

We did not consider transmembrane voltage dynamics in our sensitivity analysis because the activation at the organ level was computed with a reaction-Eikonal model without diffusion. Therefore, the conduction velocities in the Eikonal model are the only determinants of the speed of electrical propagation.

The performance of the trained GPEs is provided in Table 2. For all four calcium features, the average R^2 and ISE scores were >0.98 and >98.0, showing that the GPEs provide accurate predictions for all outputs. A Saltelli sampling with a Sobol base sequence of N_{base}=1000 samples was generated and the GPEs evaluated to compute the total order effects and to identify important parameters for the calcium features. Fig 1A shows a heatmap of the total effect of the parameters (x-axis) over the output (y-axis). The calcium transient amplitude was mostly affected by the conductance of the L-type calcium channels (P_{Ca}), which also affects all other calcium features to a lesser extent. The conductance of the sodium-calcium exchanger (G_{NCX}) had

a high effect on the diastolic calcium, which was also affected by the ratio of the L-type channels located in the subspace ($\overline{I}_{CaL,SS}$). The time to peak and the 90% relaxation time were mostly affected by the uptake of calcium in the sarcoplasmic reticulum ($\overline{J}_{up}$). The calmodulin-kinase parameters (α_{CaMK}, β_{CaMK}, $CaMK_{\mathrm{o}}$) affected all calcium transient features, but with smaller effects. The most important calcium buffer was the troponin ($\overline{[\mathrm{TRPN}]}$), which impacted the calcium decay. The sarcolemmal binding sites in the subspace $\overline{[\mathrm{BSL}]}$ and the calcium diffusion rate from the subspace to the sarcolemma had a small but significant effect on the time to peak of the calcium, while all other parameters were not significanlty important. Fig 1B shows the ranking of the parameters according to their maximum total effect across all outputs, normalised to sum up to 1. The orange bars represent the most important parameters needed to collectively explain >90% of the output features. All other parameters were excluded from the following analysis, as they did not significantly impact the calcium transient.

Table 1. ToR-ORd model parameters. The first three columns show the parameter name, its default value, the GSA and HM ranges and its meaning. The last column provides the original paper the symbol and the default value refer to. The gray rows indicate unimportant parameters as found with the GSA on the ToR-ORd model. Abbreviations and symbols: Na^+=sodium, K^+=potassium, Ca^{2+}=calcium, SR=sarcoplasmic reticulum, CaMK=calcium/calmodulin-dependent protein kinase II, DS=dyadic space.

Symbol	Default	GSA/HM range	Meaning	Reference
		**Ionic Conductances**		
G_{Na}	11.7802	±25%/-	Conductance of the fast Na^+ current	[1]
G_{NaL}	0.0279	±25%/-	Conductance of the slow Na^+ current	[1]
G_{to}	0.16	±25%/-	Conductance of the transient outward K^+ current	[1]
P_{Ca}	8.3757e-05	±25%/±75%	Conductance of the L-type Ca^{2+} current	[1]
G_{Kr}	0.0321	±25%/-	Conductance of the rapid delayed K^+ rectifier current	[1]
G_{Ks}	0.0011	±25%/-	Conductance of the slow delayed K^+ rectifier current	[1]
G_{K1}	0.6992	±25%/-	Conductance of the inward K^+ rectifier current	[1]
G_{NCX}	0.0034	±25%/±75%	Conductance of the Na^+-Ca^{2+} exchanger	[1]
G_{NaK}	15.4509	±25%/-	Conductance of the Na^+-K^+ pump	[1]
G_{Ca}	5e-04	±25%/-	Conductance of the sarcolemmal Ca^{2+} pump	[1]
G_{Kb}	0.0189	±25%/-	Conductance of the background K^+ current	[1]
P_{Nab}	1.9239e-09	±25%/-	Conductance of the background Na^+ current	[1]
P_{Cab}	5.9194e-08	±25%/-	Conductance of the background Ca^{2+} current	[2]
G_{ClCa}	0.2843	±25%/-	Conductance of the Ca^{2+}-sensitive Cl^- current	[1]
G_{Clb}	1.98e-03	±25%/-	Conductance of the background Cl^- current	[2]
		**Calcium Handling**		
$\overline{J}_{\mathrm{rel}}$	1.5378	±25%/-	Multiplier of the release Ca^{2+} current from the SR	[1]
$\overline{J}_{\mathrm{up}}$	1.0	±25%/±50%	Multiplier of the uptake of Ca^{2+} into the SR	[1]
$\overline{I}_{\mathrm{NaCa,SS}}$	0.35	±25%/-	Fraction of the Na^+-Ca^{2+} exchangers located in the subspace	[1]
$\overline{I}_{\mathrm{CaL,SS}}$	0.8	±25%/±75%	Fraction of the L-type channels located in the subspace	[1]
α_{CaMK}	0.05	±25%/±50%	Phoshorilation rate of Ca^{2+}/CaMK	[3]
β_{CaMK}	0.00068	±25%/±50%	Dephoshorilation rate of Ca^{2+}/CaMK	[3]
$\overline{CaMK_{\mathrm{o}}}$	0.05	±25%/±50%	Fraction of active Ca^{2+}/CaMK binding sites at equilibrium	[3]
$\overline{[\mathrm{CMDN}]}$	0.05	±25%/-	Max calmodulin concentration	[3]
$\overline{[\mathrm{TRPN}]}$	0.07	±25%/±50%	Max troponin C concentration	[3]
$\overline{[\mathrm{BSR}]}$	0.047	±25%/-	Max concentration of the SR binding sites in the DS	[3]
$\overline{[\mathrm{BSL}]}$	1.124	±25%/±50%	Max concentration of the sarcolemmal binding sites in the DS	[3]
$\overline{[\mathrm{CSQN}]}$	10.0	±25%/-	Max concentration of calsequestrin	[3]
$\tau_{\mathrm{diff,Ca}}$	0.2	±25%/±50%	Diffusion rate of Ca^{2+} from the cytoplasm to the DS	[3]
τ_{tr}	60.0	±25%/-	Diffusion rate of Ca^{2+} from the junctional to the network SR	[3]

Table 2. GPEs performance ToR-ORd. R^2 score and ISE for every split of a 5-fold cross-validation, reported for each output.

Model output	Meaning	Metric	fold-1	fold-2	fold-3	fold-4	fold-5	Mean
Ca_{diast}	Diastolic calcium concentration	R^2	0.9944	0.9925	0.9870	0.9926	0.9877	0.9908
		ISE	99.77	98.62	98.62	98.85	98.16	98.80
Ca_{ampl}	Transient amplitude	R^2	0.9907	0.9720	0.9829	0.9882	0.9906	0.9849
		ISE	99.54	98.16	98.62	98.85	99.77	98.99
TTP	Time to peak	R^2	0.9902	0.9873	0.9896	0.9880	0.9906	0.9891
		ISE	98.85	98.39	98.85	98.39	98.85	98.67
RT90	Time to reach 90% decay	R^2	0.9972	0.9964	0.9956	0.9974	0.9974	0.9968
		ISE	99.54	98.62	98.85	99.54	99.31	99.17

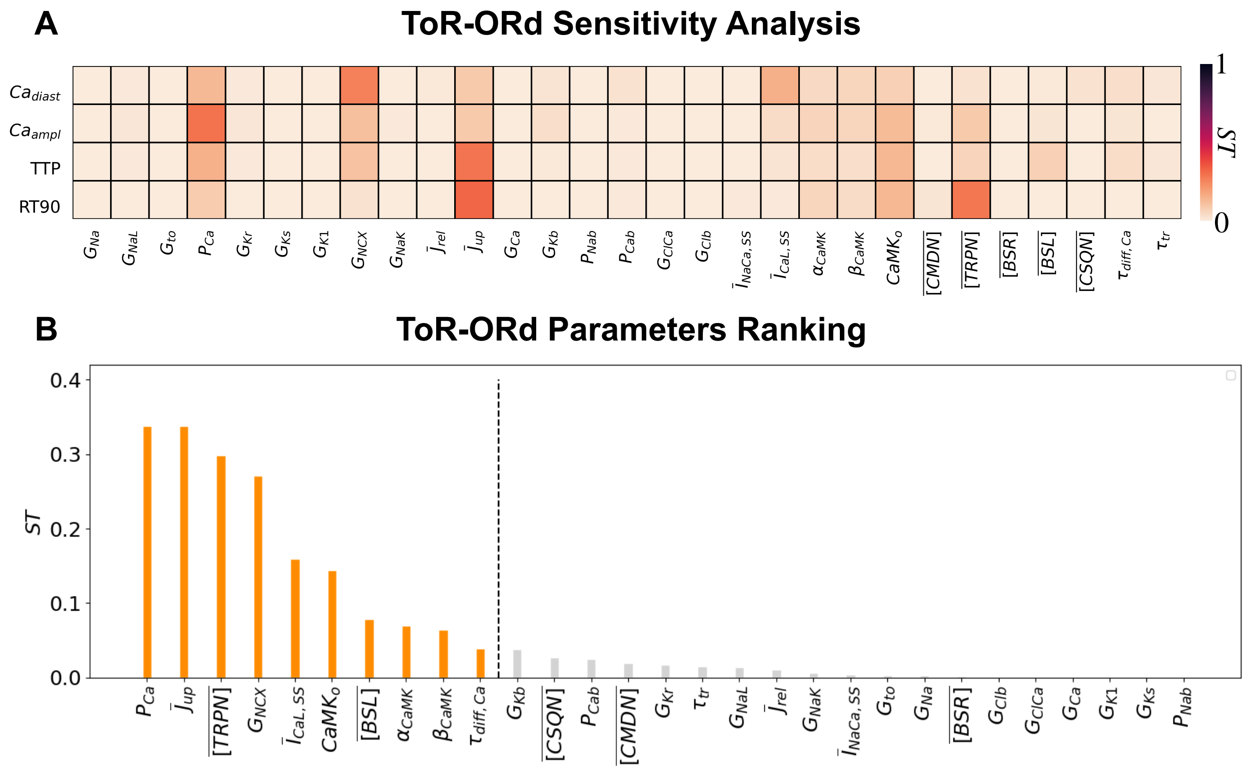

Fig 1. Sensitivity analysis results. A Heatmap of the total effect of the parameters (x-axis) on the outputs (y-axis). **B** Barplot of the maximum total effect of each parameter over all outputs. The parameters are ranked from most to least important. The dashed line separates important (orange) and unimportant (gray) parameters.

Table 3. History matching. The top section shows the mean μ and standard deviation σ for all model outputs, used as targets for the HM. The bottom section shows the settings and results for the three HM waves. From the left: threshold on the implausibility measure I_{th}, % of non-implausible points, mean and max implausibility measure, mean and max variance ratio between the GPEs and the data. *Waves where the first dataset was excluded from the GPE training.

**Model output**	μ	σ	**References**
Ca_{diast}	0.1462 μM	0.01661 μM	[4, 5]
Ca_{ampl}	0.5671 μM	0.2676 μM	[4, 5]
TTP	116.0 ms	75.3 ms	[4, 5]
RT90	375.0 ms	40.0 ms	[4]

Results

**Wave**	I_{th}	**% NIMP**	**mean** I	**max** I	**mean** V_{ratio}	**max** V_{ratio}
**wave1**	3.5	19.2%	4.4	12.1	0.61	2.4
**wave2**	3.5	93.1%	2.8	5.3	0.28	1.67
**wave3**	3.0	57.8%	2.8	4.7	0.23	1.44
**wave4***	3.0	97.1%	2.4	3.6	0.17	1.29
**wave5***	3.0	94.5%	2.4	4.2	0.06	0.9
**wave6***	3.0	98.8%	2.3	3.7	0.05	0.86
**wave7***	3.0	98.9%	2.3	3.4	0.05	0.85

History matching

In the previous section, we identified the ToR-ORd parameters that mostly affected the simulated calcium transient. In this section, we aim to use HM to find areas in the parameter space where these parameters provide physiological calcium features. The target mean μ and standard deviation σ for the calcium features were based on literature values from datasets from Coppini et al [4] and Piacentino et al [5]. Coppini et al

measured calcium transients from N=12 from healthy human ventricular myocytes extracted from the septum and the trabeculae, and provided the following values for diastolic calcium, calcium amplitude, TTP and RT90 at 1 Hz pacing: 0.14±0.01 μM, 0.35±0.02 μM, 50±10 ms and 375±40 ms. Piacentino et al recorded calcium transients in N=11 healthy human mid-myocardium myocytes and provided values for diastolic calcium, calcium amplitude and time to peak at 1 Hz pacing: 0.153±0.02 μM. 0.804±0.197 μM and 188±38 ms. These two datasets were chosen despite providing different ranges for the calcium features because we wanted to account for the high uncertainty in calcium transient measurements in the literature due to different tissue preparations. The resulting target mean and standard deviations for the calcium features are provided in Table 3, and were computed using the following formulas for mean and standard deviations for combined datasets:

$$\mu = \frac{N_1\mu_1 + N_2\mu_2}{N_1 + N_2}$$

$$\sigma = \sqrt{\frac{(N_1-1)\sigma_1^2 + (N_2-1)\sigma_2^2 + N_1(\mu_1-\mu)^2 + N_2(\mu_2-\mu)^2}{N_1+N_2-1}}\,,$$

where μ_1, σ_1, N_1 and μ_2, σ_2, N_2 are the mean, standard deviation and number of samples in the two datasets.

In the ToR-ORd model, the diastolic calcium was not calibrated to match any experimental data. Therefore, with default parameters, the simulated diastolic calcium was smaller than the physiological target values (0.074 μM vs 0.1462±0.01661 μM). To achieve physiological diastolic calcium during the HM, we increased the range for P_{Ca}, G_{NCX} and $\bar{I}_{\mathrm{CaL,SS}}$ to ±75% from their default values, as these parameters were the most important for the diastolic calcium (Fig 1A). The range for the other important parameters was set to ±50%. Table 3 summarises the results for the HM on the ToR-ORd model. The initial GPEs were trained with N=250 Latin hypercube samples, and the first HM wave was run with a 3.5 threshold on the implausibility measure. The subsequent waves were run by enriching the training set for the GPEs with N_{simul}=128 each time. From the fourth wave onwards, the initial dataset was excluded from the training set to improve GPEs performance. From the first to the last wave, the % of plausible points increased from 19.2% to 98.9% (Fig 2B, blue to red areas) and the mean implausibility measure decreased from 4.4 to 2.3, indicating that the predicted values for the output features are on average closer to the target ranges. In addition, GPE uncertainty decreased, as the mean variance ratio between uncertainty prediction and uncertainty on the data got smaller. Fig 2A shows the calcium transients simulated by the model, using samples extracted from the non-implausible regions of each HM wave. The red transients, extracted from the non-implausible area of the last wave are physiological, with features within the target ranges.

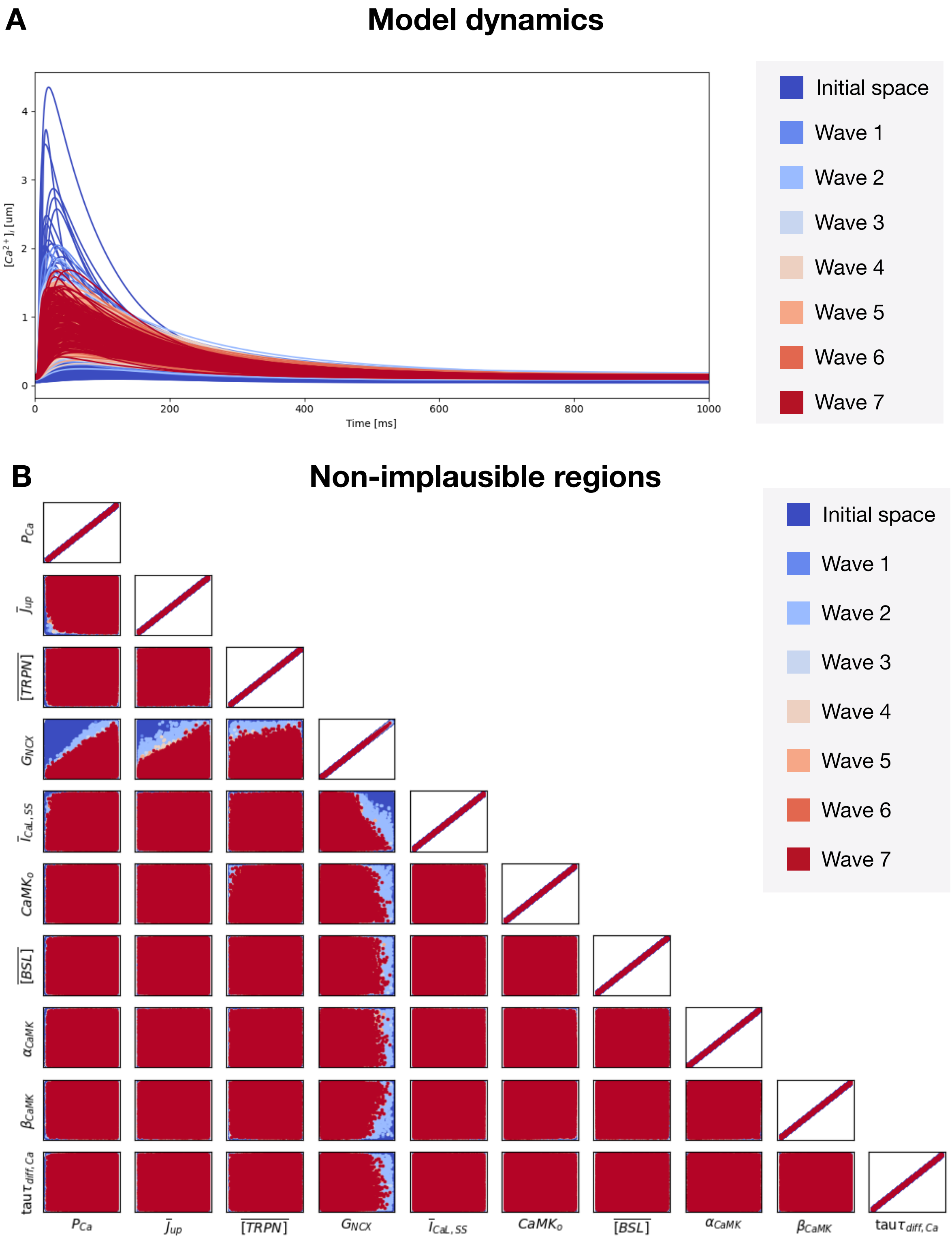

Fig 2. History matching. A Simulated calcium transient with the initial samples (blue) to the samples extracted from the non-implausible region of each wave to refine the GPEs. **B** The non-implausible area is shown, starting from the initial test samples (blue) down to the non-implausible region for the last wave (red).

ToR-ORd and Land model

In the following sections, we aim to identify important parameters for the active tension transient in three different types of simulations: 1) isometric twitch with constant stretch $\lambda = 1.0$ (e.g. no strain); 2) isometric twitch with constant stretch $\lambda = 1.1$; 3) isotonic twitch based on the Land model equation with cellular passive stress as in Jung et al [6]. We then use HM to find areas in the parameter space where both calcium and isometric tension are physiological and provide physiological dynamics at the whole organ level. All simulations were run for 100 beats with a basic cycle length of 1000 ms.

Global sensitivity analysis

For the GSA on the Land model, we considered the important parameters for the ToR-ORd model (Table 1, white rows) and all 17 parameters in the Land model. Table 4 summarises the parameters, their meaning, their default values and their range. The default for k_u and n_{Tm} were taken from [7], where the Land model was coupled to the ToR-ORd model and recalibrated. The default value for all other parameters was based on the original Land model publication [8]. The Land model parameters were constrained between ±50% from their default value, while the ToR-ORd parameters were sampled from the non-implausible region of the last HM wave (Fig 2B, red regions). The last HM wave on the ToR-ORd model provided N=98886 samples where the calcium was physiological. We then generated a N=98886 Latin hypercube samples for the Land model parameters, and used `psa_select`, a function from the python library `diversipy`, to extract N=2700 uniformly distributed samples for GPE training. The ToR-ORd and the Land model were then coupled together by giving the calcium transient in input to the Land model, and these samples were used to run simulations for isometric twitches with $\lambda = 1.0$ and $\lambda = 1.1$, and an isotonic twitch. The last beat was used to extract values for the following active tension transient features:

1. peak in tension (T_{peak})
2. time to peak in tension (TTP-T)
3. maximum active tension time derivative (dT/dt_{max})
4. minimum active tension time derivative (dT/dt_{min})
5. active tension transient duration (T_{dur})
6. rest active tension (T_{rest}).

In addition, for isotonic twich simulations, we computed the % of λ shortening ($\Delta\lambda$) to investigate the effect of calcium and tension parameters on the extent of cell contraction.

There is a high variability in isometric active tension values reported in the literature due to different tissue preparations, temperatures and species [9]. Reported values for the peak in active tension range from 10 kPa in mice trabeculae [10] up to 121±35 kPa [11] in the rat trabeculae and 108±13.8 kPa in the cat right ventricular trabeculae. Based on these literature values and to prevent the GPEs to be trained on unphysiological tension transients, we excluded samples that resulted in T_{peak}<10 kPa. In previous models, T_{rest} was constrained to be <1 kPa [12]. We relaxed this constraint to account for the effect of length dependence and excluded samples that resulted in T_{rest}>2 kPa. This provided N=1584, N=1017 and N=1100 samples for isometric twitches with $\lambda = 1.0$ and $\lambda = 1.1$, and an isotonic twitch, respectively. GPE performance is summarized in Table 5. For all active tension features, the GPEs performed well, with average R^2 and ISE scores above 0.84 and 97.0, indicating that the GPEs provide accurate prediction of model outputs. For the rest tension during an isometric twitch with $\lambda = 1.1$, the R^2 was 0.7843, which is lower than those achieved for the other outputs. This was due to highly non-linear behaviour of the Land model for non-zero strains.

The trained GPEs were used to run a GSA on the active tension transient features. To make sure that the GPEs were not evaluated outside their training space, the base sequence for the Saltelli sampling was screened with the GPEs trained during the HM on the ToR-ORd model to exclude samples providing non-physiological calcium. The base sequence sampling was repeated until the base sequence had N_{sobol}>1000 samples. Fig 3 shows the results of the GSA on the ToR-ORd-Land model. For all three simulated scenarios (isometric

$\lambda = 1.0$ and λ =1.1, and isotonic) the conductance of the L-type calcium channels (P_{Ca}) and the conductance of the sodium-calcium exchanger (G_{NCX}) affected the peak in tension, and the minimum and the maximum tension derivatives. The minimum tension derivative was also affected, but to a lesser extent, by the maximum troponin concentration ($\overline{[\text{TRPN}]}$). The reference tension T_{ref} also had an impact on the peak in tension and its derivatives, while the reference calcium sensitivity ca_{50} has a significant effect on all outputs. The rest tension was highly affected by n_{TRPN} and n_{Tm}, as they define the cooperativity between the calcium and troponin, and the calcium troponin complex with unbound cross-bridges. Cross-bridges kinetics parameters k_u, μ, $TRPN_{50}$, r_s and r_w have a significant effect on the active tension time to peak and transient duration, although their relative contribution changes during an isometric vs isotonic twitches. Finally, the velocity dependence parameter A_{eff} affects the active tension rising time and maximum tension derivative only during an isotonic twich, which is expected as during isometric twitches the velocity of contraction is 0. Fig 4A-C shows the parameter ranking according to their maximum total effect across all outputs for the three different simulated scenarios, while Fig 4D ranks the parameters according to their maximum total effect across all three simulation types. The maximum total effects were then normalised to sum up to 1, e.g. 100%. We considered important those parameters that were needed to explain >90% of output variance in all three simulations (e.g. parameters on the left of the dashed line in Fig 4D). All other parameters were excluded from the following analysis.

Table 4. ToR-ORd-Land model parameters. The first three columns show the parameter name, its default value, the GSA and HM range and its meaning. The last column provides the original paper the symbol and the default value refer to.

Symbol	Default	GSA/HM range	Meaning	Reference
			**Ionic Conductances**	
P_{Ca}	1.0299e-04	HM/±75%	Conductance of the L-type Ca^{2+} current	[1]
G_{NCX}	0.0018	HM/±75%	Conductance of the Na^{+}-Ca^{2+} exchanger	[1]
			**Calcium Handling**	
$\overline{J}_{\text{up}}$	-	HM/-	Multiplier of the uptake of Ca^{2+} into the SR	[1]
$\overline{I}_{\text{CaL,SS}}$	-	HM/-	Fraction of the L-type channels located in the subspace	[1]
α_{CaMK}	-	HM/-	Phoshorilation rate of Ca^{2+}/CaMK	[3]
β_{CaMK}	-	HM/-	Dephoshorilation rate of Ca^{2+}/CaMK	[3]
$CaMK_{\text{o}}$	-	HM/-	Fraction of active Ca^{2+}/CaMK binding sites at equilibrium	[3]
$\overline{[\text{TRPN}]}$	0.0822	HM/±50%	Max troponin C concentration	[3]
$\overline{[\text{BSL}]}$	-	HM/-	Max concentration of the sarcolemmal binding sites in the DS	[3]
$\tau_{\text{diff,Ca}}$	-	HM/-	Diffusion rate of Ca^{2+} from the cytoplasm to the DS	[3]
			**Land Model**	
T_{ref}	120/150	±50%/±33%	Reference isometric tension	[8]
n_{Tm}	2.036	±50%/±50%	Hill coefficient for Ca^{2+}-troponin and U	[7, 8]
n_{TRPN}	2.0	±50%/±50%	Ca^{2+}-troponin cooperativity	[8]
k_{TRPN}	0.1	±50%/-	Unbinding rate of Ca^{2+} from troponin	[8]
A_{eff}	25.0	±50%/±50%	Scale for distortion due to velocity of contraction	[8]
k_{u}	0.021	±50%/±50%	Transition rate from blocked to unblocked binding site	[7, 8]
β_0	2.3	±50%/-	Length-dependence parameter for tension development	[8]
β_1	-2.4	±50%/-	Length-dependence parameter for Ca^{2+} sensitivity	[8]
γ_{s}	0.0085	±50%/-	Distortion rate of strongly bound cross-bridges	[8]
γ_{w}	0.615	±50%/-	Distortion rate of weakly bound cross-bridges	[8]
ϕ	2.23	±50%/-	Distortion decay	[8]
ca_{50}	0.805	±50%/±50%	Reference Ca^{2+} sensitivity	[8]
ν	7.0	±50%/-	Scaling factor for unbound to weak cross-bridges transition rate	[8]
μ	3.0	±50%/±50%	Scaling factor for weak to strong cross-bridges transition rate	[8]
TRPN_{50}	0.35	±50%/±50%	CaTRPN when 50% of cross-bridges are blocked	[8]
r_{s}	0.25	±50%/±50%	Steady-state duty ratio	[8]
r_{w}	0.5	±50%/±50%	Steady-state ratio between pre-powerstroke and non-strongly bound	[8]

Table 5. GPEs performance ToR-ORd-Land. R^2 score and ISE for every split of a 5-fold cross-validation, reported for each output for an isometric twitch with $\lambda = 1.0$, $\lambda = 1.1$ and an isotonic twitch.

Model output	Meaning	Metric	fold-1	fold-2	fold-3	fold-4	fold-5	Mean
**Isometric $\lambda = 1.0$**								
T_{peak}	Peak in tension	R^2	0.9790	0.9765	0.9779	0.9801	0.9744	0.9776
		ISE	100.00	98.11	99.05	99.37	99.05	99.12
TTP-T	Time to peak	R^2	0.9863	0.9866	0.9896	0.9883	0.9879	0.9877
		ISE	99.37	100.00	99.37	99.68	99.68	99.62
dT/dt_{max}	Maximum derivative	R^2	0.9701	0.9668	0.9786	0.9764	0.9702	0.9724
		ISE	99.05	98.74	99.37	98.74	98.73	98.93
dT/dt_{min}	Minimum derivative	R^2	0.9574	0.9480	0.9588	0.9549	0.9436	0.9526
		ISE	99.68	97.48	98.74	98.11	97.78	98.36
T_{dur}	Transient duration	R^2	0.9809	0.9750	0.9792	0.9806	0.9778	0.9787
		ISE	99.37	99.05	99.37	99.05	99.37	99.24
T_{rest}	Rest tension	R^2	0.8542	0.8528	0.8367	0.8692	0.8300	0.8486
		ISE	97.48	98.42	97.48	97.48	97.47	97.66
**Isometric $\lambda = 1.1$**								
T_{peak}	Peak in tension	R^2	0.9747	0.9701	0.9737	0.9642	0.9519	0.9669
		ISE	99.02	97.55	97.54	97.54	95.07	97.34
TTP-T	Time to peak	R^2	0.9852	0.9846	0.9798	0.9798	0.9779	0.9815
		ISE	100.00	99.02	97.04	98.52	99.01	98.72
dT/dt_{max}	Maximum derivative	R^2	0.9678	0.9593	0.9712	0.9575	0.9407	0.9593
		ISE	98.53	97.06	97.04	97.04	96.55	97.25
dT/dt_{min}	Minimum derivative	R^2	0.9403	0.9334	0.9462	0.9310	0.9271	0.9356
		ISE	98.53	97.55	96.06	97.54	97.54	97.44
T_{dur}	Transient duration	R^2	0.9661	0.9659	0.9600	0.9654	0.9596	0.9634
		ISE	98.53	98.04	98.03	98.52	99.01	98.43
T_{rest}	Rest tension	R^2	0.7633	0.7916	0.7760	0.8097	0.7808	0.7843
		ISE	96.57	96.57	98.52	97.54	97.54	97.35
**Isotonic**								
T_{peak}	Peak in tension	R^2	0.9496	0.9384	0.9492	0.9464	0.9328	0.9433
		ISE	98.64	98.18	96.82	97.73	97.73	97.82
TTP-T	Time to peak	R^2	0.9778	0.9750	0.9736	0.9744	0.9721	0.9746
		ISE	99.55	99.09	98.64	98.64	98.18	98.82
dT/dt_{max}	Maximum derivative	R^2	0.9509	0.9476	0.9525	0.9492	0.9386	0.9478
		ISE	97.73	97.73	97.27	98.18	95.00	97.18
dT/dt_{min}	Minimum derivative	R^2	0.8513	0.8656	0.9035	0.8854	0.8917	0.8795
		ISE	97.73	95.91	96.82	98.18	98.18	97.36
T_{dur}	Transient duration	R^2	0.9513	0.9176	0.9406	0.9434	0.9520	0.9410
		ISE	98.64	95.45	96.82	98.18	98.64	97.55
T_{rest}	Rest tension	R^2	0.8417	0.8387	0.8747	0.8920	0.8483	0.8591
		ISE	98.18	97.27	99.55	100.00	97.73	98.55
$\Delta\lambda$	% Contraction	R^2	0.9545	0.9452	0.9508	0.9393	0.9417	0.9463
		ISE	98.64	99.09	97.27	97.27	98.18	98.09

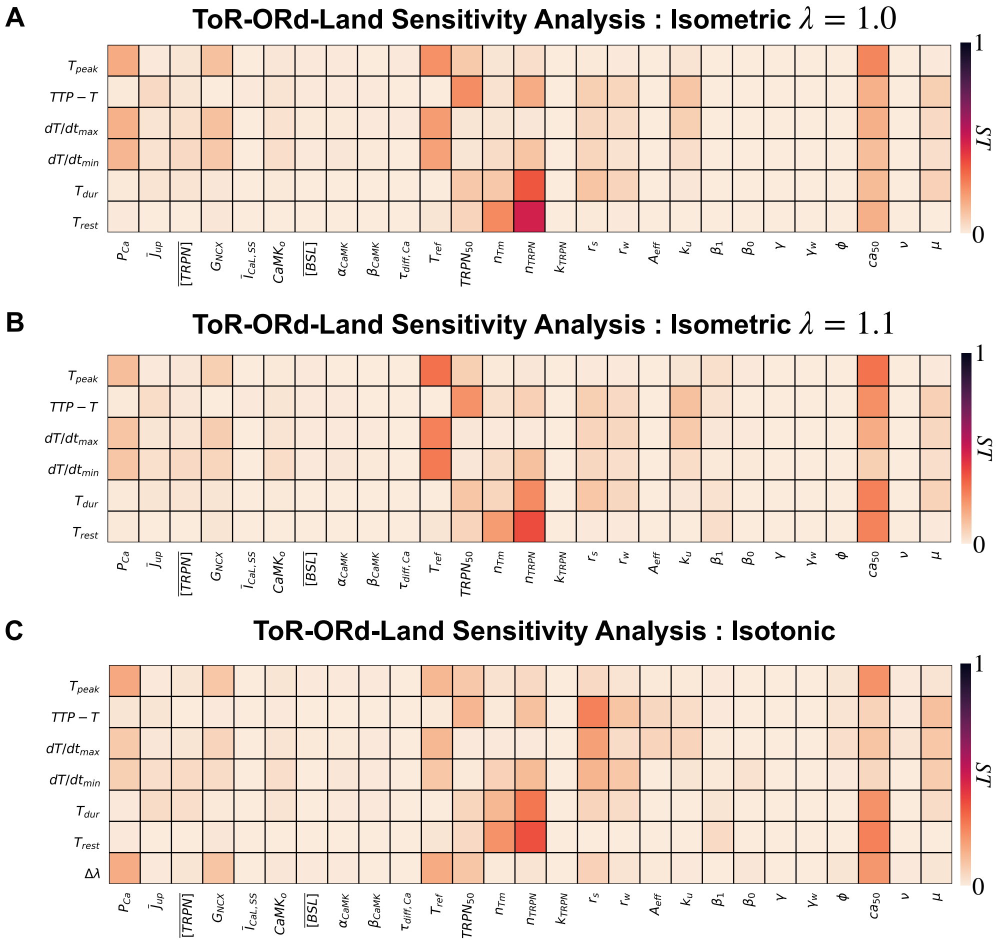

Fig 3. ToR-ORd-Land sensitivity analysis. Heatmaps representing the total effect ST of the input parameters (x-axis) over simulation output features (y-axis) for an isometric twitch with $\lambda = 1.0$ (**A**), an isometric twitch with λ=1.1 (**B**) and an isotonic twitch (**C**).

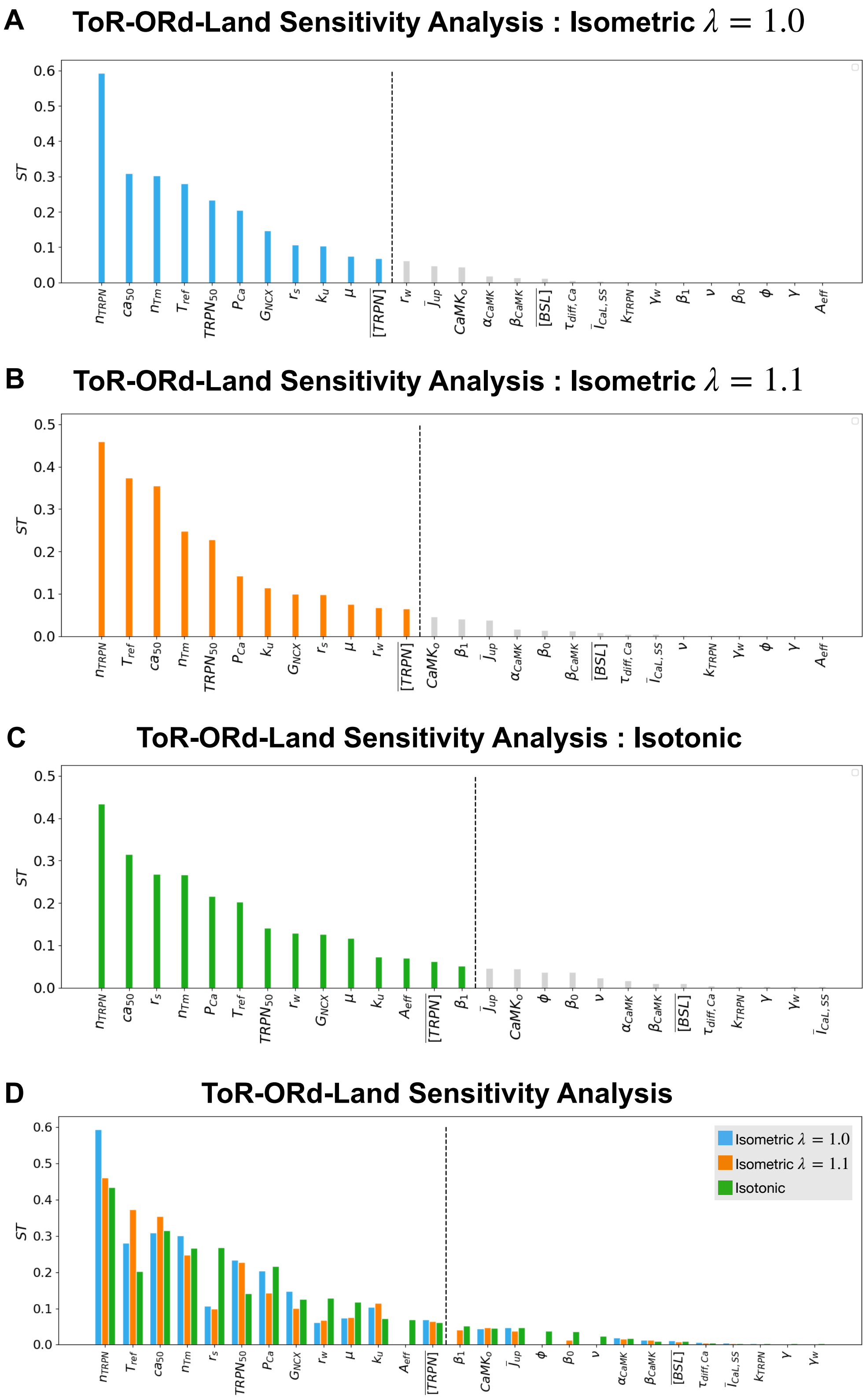

Fig 4. ToR-ORd-Land parameter ranking. Parameters ranked according to the maximum total effect across all outputs for an isometric twitch with $\lambda = 1.0$ (**A**), an isometric twitch with λ=1.1 (**B**) and an isotonic twitch (**C**). Fig **D** shows the parameter ranking of all three simulation types combined.

History matching

In the section above, we identified calcium and active tension parameters that are important for the active tension transient. In this section, we want to find areas in the parameter space where these provide both physiological calcium and active tension for an isometric twitch with $\lambda = 1.0$, and also result in physiological behaviour at the organ level. The top section of Table 6 shows the target mean values μ and standard deviations σ for the active tension features we included in the HM. The target values for the calcium features were set as explained above in the ToR-ORd model HM. The mean target peak in isometric tension was set to 160 kPa, in range with measurements in ventricular myocytes in mammals [11], with a standard deviation of 15 kPa. The duration of the active tension transient was set to 500±20 ms, consistent with pressure transient duration available from the clinical data. Finally, the rest tension target values were set to 0.5±0.1667 kPa which, together with the three-sigma rule, constrains the rest tension to be below 1 kPa, consistently with previous modelling studies [13].

Table 6. History matching. The top section shows the mean μ and standard deviation σ for all model outputs, used as targets for the HM. The bottom section shows the settings and results for the three HM waves. From the left: threshold on the implausibility measure I_{th}, % of non-implausible points, mean and max implausibility measure, mean and max variance ratio between the GPEs and the data. *Waves where the first dataset was excluded from the GPE training. **Waves where only the datasets from the previous three waves are used for the GPE training. ***GPE not used.

Model output	μ	σ	References
Ca_{diast}	0.1462 μM	0.01661 μM	[4, 5]
Ca_{ampl}	0.5671 μM	0.2676 μM	[4, 5]
TTP	116.0 ms	75.3 ms	[4, 5]
RT90	375.0 ms	40.0 ms	[4]
T_{peak}	160.0 kPa	15.0 kPa	[11]
T_{dur}	500.0 ms	20.0 ms	Clinical data
T_{rest}	0.5 kPa	0.1667 kPa	[13]

Results

**Wave**	I_{th}	**% NIMP**	**mean** I	**max** I	**mean** V_{ratio}	**max** V_{ratio}
**wave1**	3.5	2.0%	7.8	45.9	111.56	834.97
**wave2**	3.0	39.7%	3.3	11.5	20.79	288.82
**wave3**	3.0	83.4%	2.6	6.5	6.57	166.03
**wave4***	3.0	93.9%	2.5	6.0	3.71	166.98
**wave5***	3.0	90.6%	2.6	9.6	0.42	37.36
**wave6****	3.0	97.7%	2.5	4.2	0.27	19.32
**wave7****	3.0	96.8%	2.4	5.5	0.11	11.34
**wave8****	3.0	96.8%	2.4	5.5	0.11	11.34
**wave9****	3.0	99.2%	2.4	4.1	0.09	4.95
**wave10*****	3.0	93.1	2.5	18.5	-	-

The default values and parameter space bound for all parameters is provided in Table 4 (white rows). The default values for the calcium parameters was set to the center of the non-implausible region from the last HM wave on the ToR-ORd model, while all other parameters were fixed at their default value listed in Table 4. The reference tension T_{ref} was limited between 100 kPa and 200 kPa, to achieve the target value for the peak in tension of 160 kPa. The bottom section of Table 6 summarises the results for the HM iterations. The initial GPEs were trained on 260 simulations, and the first wave was run with a threshold on the non-implausibility measure of 3.5. The threshold was then decreased to 3.0 for all other waves, and the GPE traning set was enriched with N_{simul}=128 additional simulations. To improve GPEs accuracy, we excluded the initial set of simulations from the GPE training dataset for waves 4 and 5. To improve accuracy even further and avoid training the GPEs with unphysiological samples, waves 6 to 9 were run only keeping the training datasets from the last three waves. During this procedure, the percentage on non-implausible points increased from 2% to 99.2%, and the mean implausibility measure decreased from 7.8 to 2.5. Starting from very uncertain GPEs with a mean variance ratio of 111.56, indicating that the GPEs are more than a 100 times more uncertain than the experimental data, the variance ratio decreased to 0.09. By restricting the parameter space to the non-implausible area (Fig 5B, blue to red), the calcium and active tension transients used to train the GPEs go from unphysiological (Fig 5A, blue) to physiological (red).

Since the samples from the non-implausible area of the last HM wave on the ToR-ORd-Land model will

be used to run whole organ simulations, we need to ensure that the model behaves physiologically for all samples. To ensure this, we ran one last HM wave discarding the GPEs (e.g. using cell model evaluations rather than GPE predictions and setting the uncertainty of the prediction to 0). We therefore ran 200,000 test samples with the cell model extracted from the non-implausible region of wave 9, evaluated the implausibility measure for all samples, and excluded those which did not provide physiological calcium and tension outputs.

Initial tests in the whole heart simulations allowed us to identify two additional constraints that improved numerical stability of the fully coupled simulations. Specifically, we ran the following checks on each sample before running whole organ simulations:

1. isometric twitch $\lambda = 1.1$ and basic cycle length of 1000 ms: T_{rest}<2 kPa to ensure that, even for non-zero strains, the rest tension was not too high

2. isometric twitch $\lambda = 1.2$ (maximum stretch allowed in the Land model) basic cycle length of 854 ms (based on clinical data): ensure that the ODEs system for the cell model did not become too stiff, causing the system to diverge. This is often caused by the equation for CaTRPN, where the calcium sensitivity $ca_{50}(\lambda)$ is computed as a function of λ:

   $$ca_{50}(\lambda) = ca_{50} + \beta_1(\lambda - 1.0).$$

   To prevent this from happening for too many simulations, we scaled β_1 according to the value of ca_{50}:

   $$\beta_1 = \frac{\hat{\beta_1}}{c\hat{a}_{50}} ca_{50},$$

   where $c\hat{a}_{50}$=0.805 μM and $\hat{\beta_1} = -2.4$ are the default values from the original Land model [8].

This ultimately provided us with N=90906 samples for the ToR-ORd-Land model that could be used for simulations at the whole organ level.

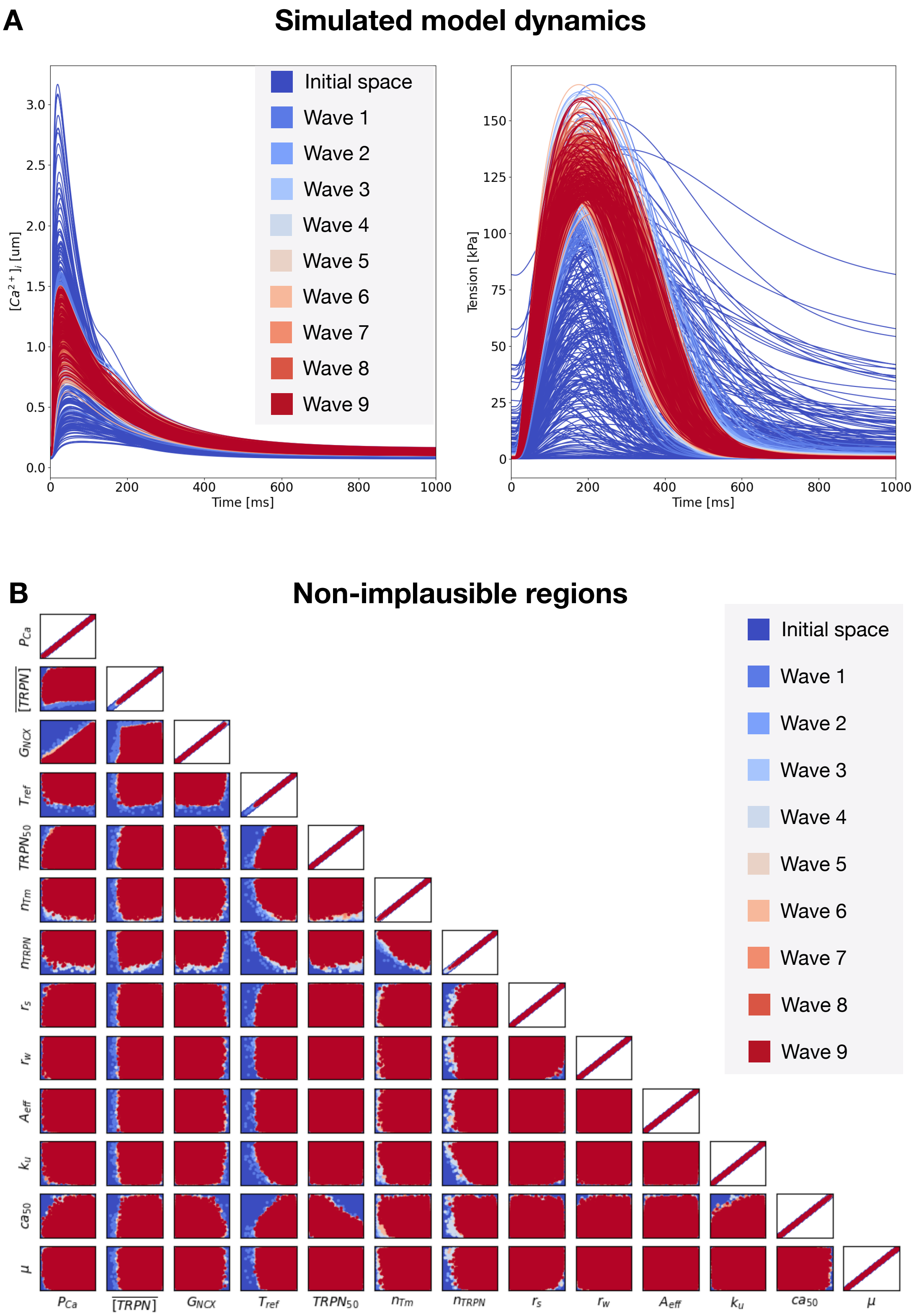

Fig 5. History matching. A Simulated calcium and tension transients with the initial samples (blue) to the samples extracted from the non-implausible region of each wave to refine the GPEs. **B** The non-implausible area is shown, starting from the initial test samples (blue) down to the non-implausible region for the last wave (red).

References

1. Tomek J, Bueno-Orovio A, Passini E, Zhou X, Minchole A, Britton O, et al. Development, calibration, and validation of a novel human ventricular myocyte model in health, disease, and drug block. Elife. 2019;8:e48890.

2. Tomek J, Bueno-Orovio A, Rodriguez B. ToR-ORd-dynCl: an update of the ToR-ORd model of human ventricular cardiomyocyte with dynamic intracellular chloride. BioRxiv. 2020;.

3. O'Hara T, Virág L, Varró A, Rudy Y. Simulation of the undiseased human cardiac ventricular action potential: model formulation and experimental validation. PLoS computational biology. 2011;7(5).

4. Coppini R, Ferrantini C, Yao L, Fan P, Del Lungo M, Stillitano F, et al. Late sodium current inhibition reverses electromechanical dysfunction in human hypertrophic cardiomyopathy. Circulation. 2013;127(5):575–584.

5. Piacentino III V, Weber CR, Chen X, Weisser-Thomas J, Margulies KB, Bers DM, et al. Cellular basis of abnormal calcium transients of failing human ventricular myocytes. Circulation research. 2003;92(6):651–658.

6. Jung A, Gsell MA, Augustin CM, Plank G. An Integrated Workflow for Building Digital Twins of Cardiac Electromechanics—A Multi-Fidelity Approach for Personalising Active Mechanics. Mathematics. 2022;10(5):823.

7. Margara F, Wang ZJ, Levrero-Florencio F, Santiago A, Vázquez M, Bueno-Orovio A, et al. In-silico human electro-mechanical ventricular modelling and simulation for drug-induced pro-arrhythmia and inotropic risk assessment. Progress in biophysics and molecular biology. 2021;159:58–74.

8. Land S, Park-Holohan SJ, Smith NP, dos Remedios CG, Kentish JC, Niederer SA. A model of cardiac contraction based on novel measurements of tension development in human cardiomyocytes. J Mol Cell Cardiol. 2017;106:68–83.

9. Niederer S, Hunter P, Smith N. A quantitative analysis of cardiac myocyte relaxation: a simulation study. Biophysical journal. 2006;90(5):1697–1722.

10. Stuyvers BD, McCulloch AD, Guo J, Duff HJ, ter Keurs HE. Effect of stimulation rate, sarcomere length and Ca2+ on force generation by mouse cardiac muscle. The Journal of physiology. 2002;544(3):817–830.

11. Backx PH, Gao W, Azan-Backx MD, Marban E. The relationship between contractile force and intracellular [Ca2+] in intact rat cardiac trabeculae. The Journal of General Physiology. 1995;105(1):1–19.

12. Land S, Niederer SA. A spatially detailed model of isometric contraction based on competitive binding of troponin I explains cooperative interactions between tropomyosin and crossbridges. PLoS Computational Biology. 2015;11(8):e1004376.

13. Land S, Niederer SA, Aronsen JM, Espe EKS, Zhang L, Louch WE, et al. An analysis of deformation-dependent electromechanical coupling in the mouse heart. The Journal of physiology. 2012;590(18):4553–4569.
