# Supplementary material for: Cell to whole organ global sensitivity analysis on a four-chamber heart electromechanics model using Gaussian processes emulators
Source: PLoS Comput Biol. 2023 Jun 26;19(6):e1011257. doi: 10.1371/journal.pcbi.1011257 (PMC10328347; doi:10.1371/journal.pcbi.1011257)
Supplement: S3 File — We trained GPEs to predict calcium and active tension transient features simulated by the Courtemanche model coupled with the Land contraction model, and used them to run a GSA to identify important parameters for these dynamics. Then, we used HM to isolate areas in the parameter space where the atrial calcium and tension were physiological. (PDF) [file pcbi.1011257.s003.pdf]

Courtemanche and Land model sensitivity analysis

We used Gaussian process emulators (GPEs) and global sensitivity analysis (GSA) to identify the important parameters of the Courtemanche model coupled with the Land model. First, we ran the analysis on the Courtemanche model alone to identify important parameters for the calcium transient features. We then used Bayesian history matching (HM) to isolate the parameter areas where the calcium transient was physiological. The Courtemanche model was then coupled with the Land model to run a GSA and to investigate which ionic model and active tension parameters affected the active tension transient features. Finally, HM was used to find the areas of the parameter space where the active tension was physiological.

Courtemanche model

Global sensitivity analysis

Table 1 shows the parameters we included in the analysis on the Courtemanche model. We considered the conductances for all ionic currents: sodium g_{Na}, transient outward potassium current g_{to}, L-type calcium current g_{CaL}, rapid and slow delayed potassium rectifier current g_{Kr} and g_{Ks}, inward potassium rectifier current g_{K1}, background sodium and calcium currents $g_{b,Na}$ and $g_{b,Ca}$, ultra-rapid delayed rectifier current $\overline{g}_{Kur}$, sodium-calcium exchanger current $I_{NaCa(max)}$, sodium-potassium pump $I_{NaK(max)}$ and sarcolemmal pump calcium current $I_{p,Ca(max)}$; and parameters for calcium handling: calcium uptake into the network sarcoplasmic reticulum (SR) $I_{up(max)}$, rate of calcium release into the junctional SR $\overline{k}_{rel}$, calcium buffers concentrations [Cmdn], [Trpn] and [Csqn], and calcium diffusion rate τ_{tr}. All default values were based on the original publication [1]. For GPE training and GSA, the range for all parameters was set to ±25% from the default value. The parameter space was sampled with a Latin hypercube design with N=1350 points. For each sample, we ran the Courtemanche model for 100 beats and a cycle length of 1000 ms. The last beat was used to compute the following calcium transient features:

1. diastolic calcium level (Ca_{diast})
2. calcium transient amplitude (Ca_{ampl})
3. maximum calcium time derivative ($\text{dCa/dt}_{\text{max}}$)
4. time to reach 90% of calcium transient decay (RT90).

Similarly to the ToR-ORd model, we did not consider transmembrane voltage dynamics in our sensitivity analysis because the activation at the organ level was computed with a reaction-Eikonal model without diffusion. Therefore, the conduction velocities in the Eikonal model are the only determinants of the speed of electrical propagation.

Table 2 provides the R^2 and ISE scores for all outputs for all five splits in a 5-fold cross validation and on average. All mean R^2 and ISE scores were above 0.99 and 98, respectively, showing that the GPEs were able to provide accurate predictions for all outputs. A Saltelli sampling with a Sobol base sequence of N_{base}=1000 samples was generated and the GPEs evaluated to compute the total order effects and to identify important parameters for the calcium features. In Fig 1A we show a heatmap of the total effect of input parameters (x-axis) over the outputs (y-axis). The conductance of the L-type calcium channels g_{CaL} affects the diastolic calcium, the calcium transient amplitude and the maximum time derivative. The diastolic calcium is also affected by the background calcium $g_{b,Ca}$ and by the sodium-calcium exchangers $I_{NaCa(max)}$. Calcium uptake into the SR affects calcium decay (RT90) the most, but also the amplitude and the maximum time derivative.

Troponin ([Trpn]), which is the most important calcium buffer, affects the same outputs. The calmodulin concentration [Cmdn] and the calcium diffusion rate τ_{tr} have small but detectable effects on RT90 and calcium amplitude, and the diastolic calcium, respectively. Fig 1B shows the parameter ranking according to their maximum total effect across all outputs. The maximum total effects were normalised to sum up to 100% and the most important parameters needed to explain >90% of output variance were classified as important (orange bars). All other parameters were excluded from the next analysis, as they did not significantly impact the calcium transient.

Table 1. Model parameters. The four columns show the parameter name, its default value, the GSA and HM range and its meaning. The last column provides the original paper the symbol refers to. The blue and gray rows indicate parameters that were excluded from the analysis because they could be estimated from the clinical data or they were unimportant. Abbreviations and symbols: Na^+=sodium, K^+=potassium, Ca^{2+}=calcium, SR=sarcoplasmic reticulum, and SR=sarcoplasmic reticulum.

Symbol	Default	GSA/HM range	Meaning	Reference
**Ionic Conductances**				
g_{Na}	7.8	±25%/-	Conductance of the fast Na^+ current	[1]
g_{to}	0.1652	±25%/-	Conductance of the transient outward K^+ current	[1]
$g_{\mathrm{Ca,L}}$	0.1238	±25%/±50%	Conductance of the L-type Ca^{2+} current	[1]
g_{Kr}	0.0294	±25%/-	Conductance of the rapid delayed K^+ rectifier current	[1]
g_{Ks}	0.129	±25%/-	Conductance of the slow delayed K^+ rectifier current	[1]
g_{K1}	0.09	±25%/-	Conductance of the inward K^+ rectifier current	[1]
$g_{\mathrm{b,Na}}$	0.000674	±25%/-	Conductance of the background Na^+ current	[1]
$g_{\mathrm{b,Ca}}$	0.00113	±25%/±50%	Conductance of the background Ca^{2+} current	[1]
$\overline{g}_{\mathrm{Kur}}$	1.0	±25%/±50%	Ultra-rapid rectifier K^+ current scaling factor	[1]
$I_{\mathrm{NaCa(max)}}$	1600	±25%/±50%	Na^+-Ca^{2+} exchanger scaling factor	[1]
$I_{\mathrm{NaK(max)}}$	0.60	±25%/-	Maximum Na^+-K^+ pump current	[1]
$I_{\mathrm{p,Ca(max)}}$	0.275	±25%/-	Maximum sarcoplasmic Ca^{2+} pump current	[1]
**Calcium Handling**				
$I_{\mathrm{up(max)}}$	0.005	±25%/±50%	Maximum Ca^{2+} uptake rate into the network SR	[1]
$\overline{k}_{\mathrm{rel}}$	30.0	±25%/-	Maximum Ca^{2+} release rate from junctional SR	[1]
$[\mathrm{Cmdn}]_{\mathrm{max}}$	0.05	±25%/±50%	Total calmodulin concentration in cytoplasm	[1]
$[\mathrm{Trpn}]_{\mathrm{max}}$	0.07	±25%/±50%	Total troponin C concentration in cytoplasm	[1]
$[\mathrm{Csqn}]_{\mathrm{max}}$	10.0	±25%/-	Total calsequestrin concentration in junctional SR	[1]
τ_{tr}	180.0	±25%/±50%	Ca^{2+} transfer time constant	[1]

Table 2. GPEs performance Courtemanche. R^2 score and ISE for every split of a 5-fold cross-validation, reported for each output.

Model output	Meaning	Metric	fold-1	fold-2	fold-3	fold-4	fold-5	Mean
Ca_{diast}	Diastolic calcium concentration	R^2	0.9990	0.9991	0.9992	0.9992	0.9987	0.9990
		ISE	99.63	99.26	100.00	99.63	99.26	99.56
Ca_{ampl}	Transient amplitude	R^2	0.9987	0.9980	0.9980	0.9987	0.9978	0.9982
		ISE	99.63	98.89	99.63	100.00	98.52	99.33
dCa/dt_{max}	Maximum derivative	R^2	0.9985	0.9977	0.9979	0.9985	0.9972	0.9980
		ISE	99.63	99.26	99.26	100.00	98.52	99.33
RT90	Time to reach 90% decay	R^2	0.9965	0.9976	0.9982	0.9960	0.9974	0.9972
		ISE	97.41	98.89	98.52	98.15	98.15	98.22

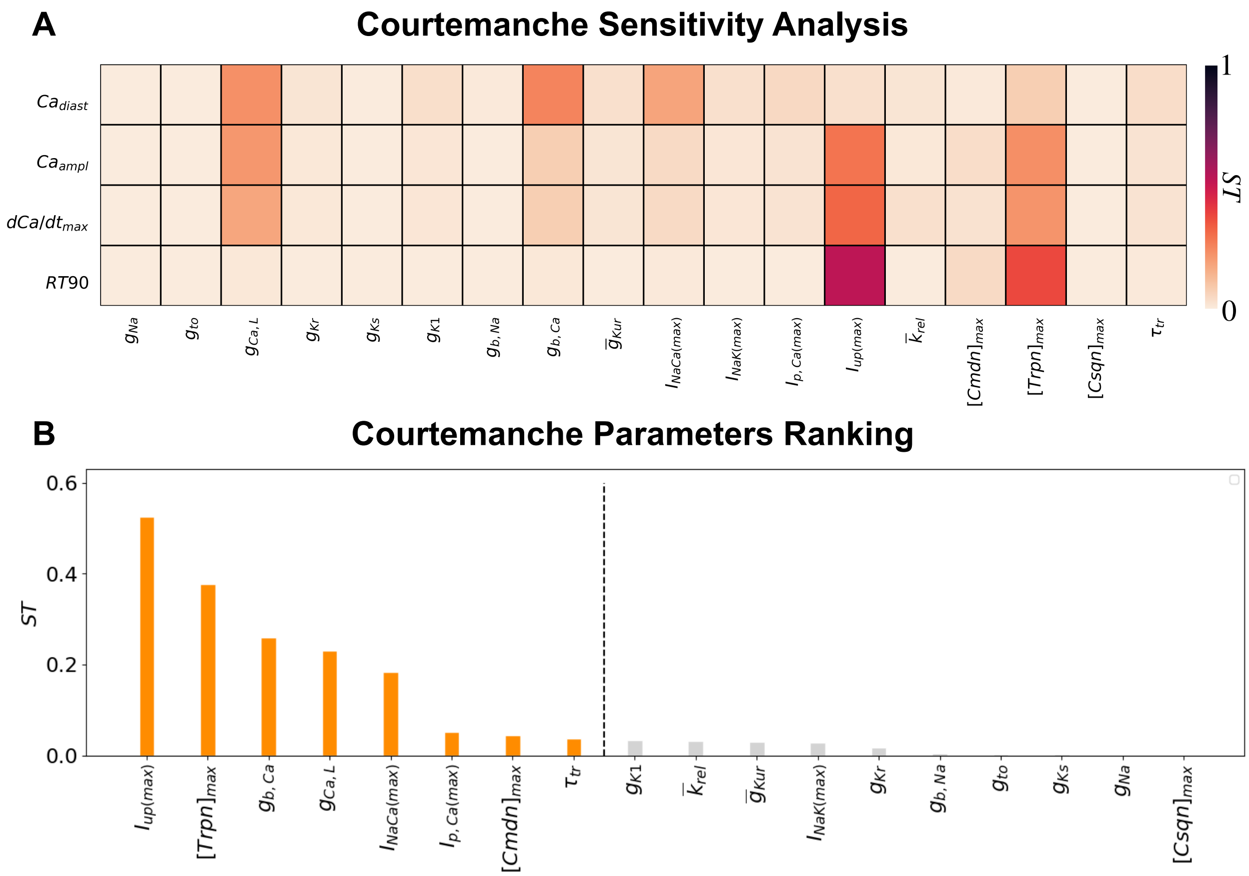

Fig 1. Sensitivity analysis results. A Heatmap of the total effect of the parameters (x-axis) on the outputs (y-axis). **B** Barplot of the maximum total effect of each parameter over all outputs. The parameters are ranked from most to least important. The dashed line separates important (orange) and unimportant (gray) parameters.

History matching

In the previous section, we used a GSA to identify the important parameters for the simulated calcium transient. In this section, HM is used to bound the important parameters to areas where the calcium features are physiological. The top section of Table 3 shows the mean μ and standard deviation σ values used for the HM on the Courtemanche model. We set the target mean and standard deviation for the calcium amplitude and the diastolic calcium to the same values used for the ToR-ORd model (see Supplement ToR-ORd-Land), consistent with literature data reporting similar values for atria and ventricles [2]. In Fig 2B in Land et al [3], the authors compared calcium transients from ventricular vs atrial myocytes experimentally measured by Coppini et al [4] and Brixius et al [5], respectively. The maximum time derivative for the calcium was three times bigger for the atria compared to the ventricles. The mean and standard deviation provided by Piacentino et al [6] for the calcium maximum derivative for ventricular myocytes ($0.0292 \pm 0.0086 \mu M/s$) were therefore multiplied by three to account for faster atrial calcium rising. Similarly, since RT90 was 1.1 times longer for the atria compared to the ventricles due to atrial shorter time to peak, the RT90 mean and standard deviation from Coppini et al [4] provided for the ventricles were scaled accordingly, leading to 413±44 ms.

Table 3. History matching. The top section shows the mean μ and standard deviation σ for all outputs, used as targets for the HM. The bottom section shows the settings and results for the three HM waves. From the left: threshold on the implausibility measure I_{th}, % of non-implausible points, mean and max implausibility measure, mean and max variance ratio between the GPEs and the data.

**Model output**	μ	σ	**References**
Ca_{diast}	0.1462 μM	0.01605 μM	[4, 6]
Ca_{ampl}	0.5671 μM	0.2643 μM	[4, 6]
$\text{dCa/dt}_{\text{max}}$	0.0876 μM/ms	0.00258 μM/ms	[3, 6]
RT90	413.0 ms	44.0 ms	[3, 4]

Results

**Wave**	I_{th}	**% NIMP**	**mean** I	**max** I	**mean** V_{ratio}	**max** V_{ratio}
**wave1**	3.5	55.4	3.4	17.3	0.08	0.59
**wave2**	3.5	97.1	2.4	4.5	0.05	0.37
**wave3**	3.0	76.3	2.4	4.4	0.03	0.26
**wave4***	3.0	98.6	2.1	3.6	0.03	0.16
**wave5***	3.0	98.6	2.1	3.8	0.02	0.3
**wave6***	3.0	99.5	2.1	3.3	0.01	0.31

The range for all important parameters (Table 1, white lines) was set to ±50%, while all other parameters were fixed to the default values based on the original publication. The HM results are summarised in Table 2. The initial GPEs were trained with N=200 Latin hypercube samples, and the first HM wave was run with a 3.5 threshold on the implausibility measure. The subsequent waves were run by enriching the training set for the GPEs with $\text{N}_{\text{simul}}=128$ each time. From the fourth wave onwards, the initial dataset was excluded from the training set to improve GPEs performance. From the first to the last wave, the % of plausible points increased from 55.4% to 99.5% (Fig 2B, blue to red areas) and the mean implausibility measure decreased from 3.4 to 2.1, indicating that the predicted values for the output features are on average closer to the target ranges. In addition, GPE accuracy increased, as the mean variance ratio between uncertainty prediction and uncertainty on the data got smaller. Fig 2A shows the calcium transients simulated with the Courtemanche model, using samples extracted from the non-implausible regions of each HM wave. The red transients, extracted from the non-implausible area of the last wave, are physiological, with features within the target ranges.

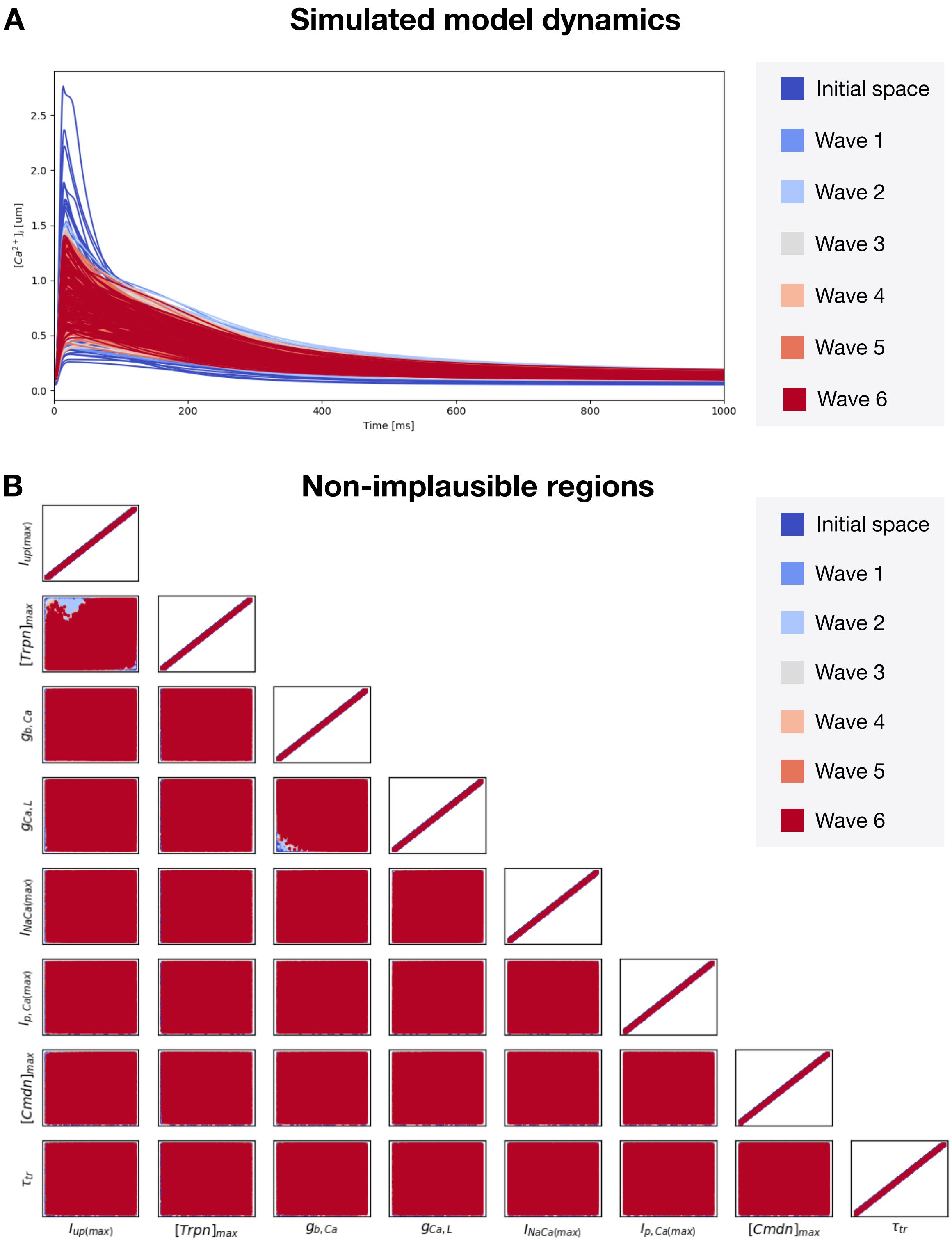

Fig 2. History matching. A Calcium transients simulated for the initial samples (blue) and the samples extracted from the non-implausible area to refine the GPEs in all HM waves. **B** The non-implausible area is shown, starting from the initial test samples (blue) down to the non-implausible region for the last wave (red).

Courtemanche and Land model

In the following sections, we used GPEs and a GSA to identify important parameters for the active tension transient in three different types of mechanical cell simulations: 1) isometric twitch with constant stretch $\lambda = 1.0$ (e.g. no strain); 2) isometric twitch with constant stretch $\lambda = 1.1$; 3) isotonic twitch based on the Land model equation with cellular passive stress as in Jung et al [7]. Then, HM was used to find areas in the parameter space where both calcium and isometric tension are physiological and do not cause instabilities in the whole organ simulations. All simulations were run for 100 beats with a basic cycle length of 1000 ms.

Global sensitivity analysis

For the GSA on the atrial active tension, we considered the important parameters for the Courtemanche model (Table 1, white rows) and all 17 parameters in the Land model. Table 4 summarises the parameters, their meaning, their default values and their range. The default for ca_{50} and μ were taken from [3], where the original Land cell model was adapted to replicate faster cross-bridges kinetics in the atria compared to the ventricles. The default value for all other parameters was based on the original Land model [8]. The Land model parameters were constrained between ±50% from their default value, while the Courtemanche parameters were sampled from the non-implausible region of the last HM wave (Fig 2B, red regions). The last HM wave on the Courtemanche model provided N=99482 samples where the calcium was physiological. We then generated a N=99482 Latin hypercube samples for the Land model parameters, and used `psa_select`, a function from the python library `diversipy`, to extract N=2500 uniformly distributed samples for GPE training. The Courtemanche and the Land model were then coupled together by giving the calcium transient in input to the Land model, and these samples were used to run simulations for isometric twitches with $\lambda = 1.0$ and $\lambda = 1.1$, and an isotonic twitch. The last beat was used to extract values for the following active tension transient features:

1. peak in tension ($\mathrm{T_{peak}}$)
2. time to peak in tension (TTP-T)
3. maximum active tension time derivative ($\mathrm{dT/dt_{max}}$)
4. minimum active tension time derivative ($\mathrm{dT/dt_{min}}$)
5. active tension transient duration ($\mathrm{T_{dur}}$)
6. rest active tension ($\mathrm{T_{rest}}$).

In addition, for isotonic twich simulations, we computed the % of λ shortening ($\Delta\lambda$) to investigate the effect of calcium and tension parameters on the extent of cell contraction.

Similarly to the analysis we did on the ToR-ORd-Land model (see Supplement ToR-ORd), we excluded non-physiological active tension transients by constraining $\mathrm{T_{peak}}$ and $\mathrm{T_{rest}}$ to be above 10 kPa [9] and below 2 kPa [10], respectively, to prevent the GPEs to be trained on non-physiological samples. After filtering, the GPEs were trained using N=1957, N=1666 and N=1690 for an isometric twitches with $\lambda = 1.0$ and $\lambda = 1.1$, and an isotonic twitch, respectively. GPE performance is summarized in Table 5. For all active tension features, the GPEs performed well, with average R^2 and ISE scores above 0.82 and 96.0, indicating that the GPEs provide accurate prediction of model outputs. For the rest tension during an isometric twitch with $\lambda = 1.1$, the R^2 was 0.7904, which is lower than those achieved for the other outputs due to highly non-linear behaviour of the Land model for non-zero strains.

We used the GPEs to run a GSA to identify which parameters affect the active tension transient features the most. We ensured that the GPEs were not evaluated outside their training space by screening the samples for the base sequence prior to the Saltelli sampling. To do this, the GPEs trained to predict the calcium features during the HM in the sections above were used to exclude samples that resulted in unphysiological calcium features. The base sequence sampling was repeated with an increasing number of samples until the base sequence had $\mathrm{N_{sobol}}$>1000 samples. Fig 1A-C shows heatmaps of the total effect of the input parameters (x-axis) over the tension features (y-axis) for the isometric and the isotonic simulations. The calcium parameters ($I_{up(max)}$, $\mathrm{[Trpn]_{max}}$ and g_{CaL}) played a more important role in the isometric tension

Table 4. Courtemanche-Land model parameters. The first and the second columns show the parameter name and its meaning. The last column provides the original paper the symbol refers to. The blue and gray rows indicate parameters that were excluded from the analysis because they could be estimated from the clinical data or they were unimportant.

Symbol	Default	GSA/HM range	Meaning	Reference
**Ionic Conductances**				
$g_{\mathrm{Ca,L}}$	0.1332	HM/±50%	Conductance of the L-type Ca^{2+} current	[1]
$g_{\mathrm{b,Ca}}$	-	HM/-	Conductance of the background Ca^{2+} current	[1]
$\overline{g}_{\mathrm{Kur}}$	-	HM/-	Ultra-rapid rectifier K^+ current scaling factor	[1]
$I_{\mathrm{NaCa(max)}}$	-	HM/-	Na^+-Ca^{2+} exchanger scaling factor	[1]
$I_{\mathrm{up(max)}}$	0.00537	HM/±50%	Maximum Ca^{2+} uptake rate into the network SR	[1]
**Calcium Handling**				
$[\mathrm{Cmdn}]_{\mathrm{max}}$	-	HM/-	Total calmodulin concentration in cytoplasm	[1]
$[\mathrm{Trpn}]_{\mathrm{max}}$	0.069694	HM/±50%	Total troponin C concentration in cytoplasm	[1]
τ_{tr}	-	HM/-	Ca^{2+} transfer time constant	[1]
**Land Model**				
T_{ref}	120/100	±50%/±20%	Reference isometric tension	[8]
n_{Tm}	5.0	±50%/±50%	Hill coefficient for Ca^{2+}-troponin and U	[8]
n_{TRPN}	2.0	±50%/±50%	Ca^{2+}-troponin cooperativity	[8]
k_{TRPN}	0.1	±50%/-	Unbinding rate of Ca^{2+} from troponin	[8]
A_{eff}	25.0	±50%/±50%	Scale for distortion due to velocity of contraction	[8]
k_{u}	1.0	±50%/-	Transition rate from blocked to unblocked binding site	[8, 11]
β_0	2.3	±50%/-	Length-dependence parameter for tension development	[8]
β_1	-2.4	±50%/-	Length-dependence parameter for Ca^{2+} sensitivity	[8]
γ_{s}	0.0085	±50%/-	Distortion rate of strongly bound cross-bridges	[8]
γ_{w}	0.615	±50%/-	Distortion rate of weakly bound cross-bridges	[8]
ϕ	2.23	±50%/±50	Distortion decay	[8]
ca_{50}	0.86	±50%/±50%	Reference Ca^{2+} sensitivity	[8]
ν	7.0	±50%/-	Scaling factor for unbound to weak cross-bridges transition rate	[3]
μ	9.0	±50%/±50%	Scaling factor for weak to strong cross-bridges transition rate	[3]
TRPN_{50}	0.35	±50%/±50%	CaTRPN when 50% of cross-bridges are blocked	[8]
r_{s}	0.25	±50%/±50%	Steady-state duty ratio	[8]
r_{w}	0.5	±50%/±50%	Steady-state ratio between pre-powerstroke and non-strongly bound	[8]

when there is no strain. In particular, the conductance of the L-type calcium channels affected the peak in tension and its derivatives, while the troponin concentration and the calcium uptake into the SR had a significant impact on the time to peak and the minimum time derivative. The peak in tension and its derivatives were also affected by the reference tension T_{ref}, ca_{50}, and $TRPN_{50}$. Outputs representing tension decay (duration of the transient, minimum derivative and rest tension) were stroncly affected by $TRPN_{50}$, n_{TRPN} and $ca50$. Finally, the cross-bridges kinetic parameter (r_s, r_w and μ) affected the timing of contraction. The relative importance of the calcium parameters decreases in favour of T_{ref} and velocity dependence (A_{eff} and ϕ) during an isometric twitch with λ=1.1 and an isotonic twitch, respectively. Fig 4A-C shows the parameter ranking for the three different simulated scenarios, while Fig 4D ranks the parameters according to their maximum total effect across all three simulation types. The maximum total effects were then normalised to sum up to 1 (e.g. 100% of output variance). We considered important those parameters that were needed to explain >90% of output variance in all three simulations (e.g. parameters on the left of the dashed line in Fig 4D). All other parameters were excluded from the following analysis.

Table 5. GPEs performance Courtemanche-Land. R^2 score and ISE for every split of a 5-fold cross-validation, reported for each output for an isometric twitch with $\lambda = 1.0$, $\lambda = 1.1$ and an isotonic twitch.

Model output	Meaning	Metric	fold-1	fold-2	fold-3	fold-4	fold-5	Mean
**Isometric $\lambda = 1.0$**								
T_{peak}	Peak in tension	R^2	0.9699	0.9770	0.9679	0.9712	0.9704	0.9713
		ISE	99.23	99.74	98.72	99.49	97.95	99.03
TTP-T	Time to peak	R^2	0.9691	0.9565	0.9518	0.9593	0.9611	0.9596
		ISE	98.72	98.72	98.72	99.23	99.23	98.93
dT/dt_{max}	Maximum derivative	R^2	98.47	99.23	99.23	98.98	98.21	98.82
		ISE	99.05	98.74	99.37	98.74	98.73	98.93
dT/dt_{min}	Minimum derivative	R^2	0.8846	0.9058	0.9152	0.9119	0.9078	0.9051
		ISE	96.68	97.70	97.19	98.21	97.95	97.55
T_{dur}	Transient duration	R^2	0.9597	0.9711	0.9573	0.9663	0.9732	0.9655
		ISE	99.49	98.98	98.98	99.74	99.23	99.28
T_{rest}	Rest tension	R^2	0.8502	0.7976	0.8671	0.8194	0.8148	0.8298
		ISE	98.47	97.19	97.19	97.19	96.42	97.29
**Isometric $\lambda = 1.1$**								
T_{peak}	Peak in tension	R^2	0.9732	0.9686	0.9675	0.9735	0.9673	0.9700
		ISE	99.10	98.2	99.1	98.8	98.5	98.74
TTP-T	Time to peak	R^2	0.9665	0.9365	0.9589	0.9532	0.9640	0.9558
		ISE	98.80	96.4	97.9	98.5	98.5	98.02
dT/dt_{max}	Maximum derivative	R^2	99.40	99.1	98.5	98.8	98.8	98.92
		ISE	98.53	97.06	97.04	97.04	96.55	97.25
dT/dt_{min}	Minimum derivative	R^2	0.8907	0.9128	0.8983	0.8744	0.8975	0.8947
		ISE	97.01	99.1	97.9	97.6	98.5	98.02
T_{dur}	Transient duration	R^2	0.9488	0.9388	0.9526	0.9521	0.9495	0.9484
		ISE	98.50	98.5	97.3	99.1	99.1	98.50
T_{rest}	Rest tension	R^2	0.8014	0.7594	0.8127	0.7942	0.7844	0.7904
		ISE	96.71	97.9	96.4	96.1	97.0	96.82
**Isotonic**								
T_{peak}	Peak in tension	R^2	0.9504	0.9481	0.9465	0.9441	0.9309	0.9440
		ISE	98.22	98.82	97.93	98.22	97.63	98.17
TTP-T	Time to peak	R^2	99.11	98.22	97.93	98.52	97.93	98.34
		ISE	99.55	99.09	98.64	98.64	98.18	98.82
dT/dt_{max}	Maximum derivative	R^2	0.9678	0.9698	0.9648	0.9637	0.9655	0.9663
		ISE	99.11	99.11	97.63	98.82	99.41	98.82
dT/dt_{min}	Minimum derivative	R^2	0.8513	0.8656	0.9035	0.8854	0.8917	0.8795
		ISE	97.04	96.75	97.04	97.34	97.63	97.16
T_{dur}	Transient duration	R^2	0.9557	0.9382	0.9525	0.9449	0.9584	0.9499
		ISE	98.82	97.34	97.34	97.04	99.11	97.93
T_{rest}	Rest tension	R^2	0.8367	0.7197	0.8151	0.8211	0.8303	0.8046
		ISE	97.34	95.86	97.34	97.04	97.34	96.98
$\Delta\lambda$	% Contraction	R^2	0.9568	0.9562	0.9570	0.9630	0.9569	0.9580
		ISE	98.52	97.93	98.22	98.82	99.11	98.52

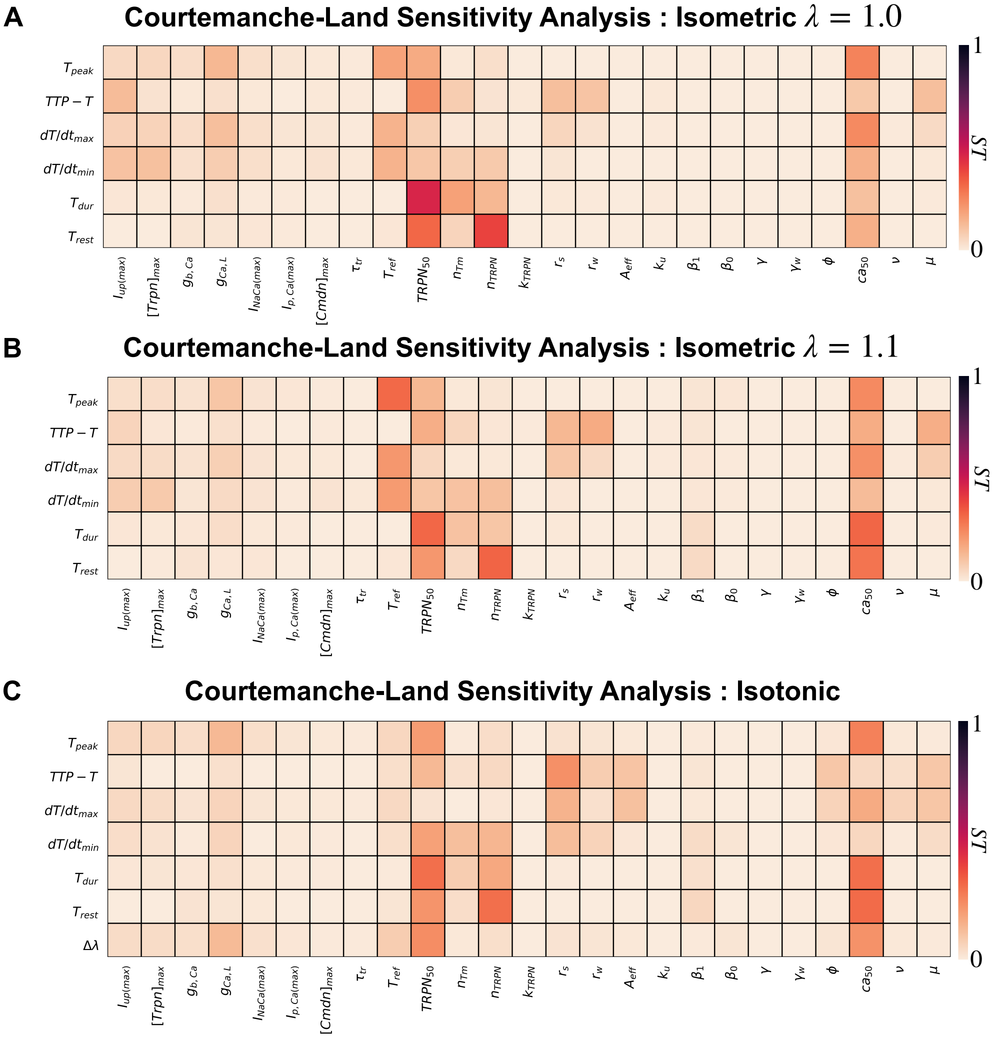

Fig 3. Courtemanche-Land sensitivity analysis. Heatmaps representing the total effect ST of the input parameters (x-axis) over simulation output features (y-axis) for an isometric twitch with $\lambda = 1.0$ (**A**), an isometric twitch with λ=1.1 (**B**) and an isotonic twitch (**C**).

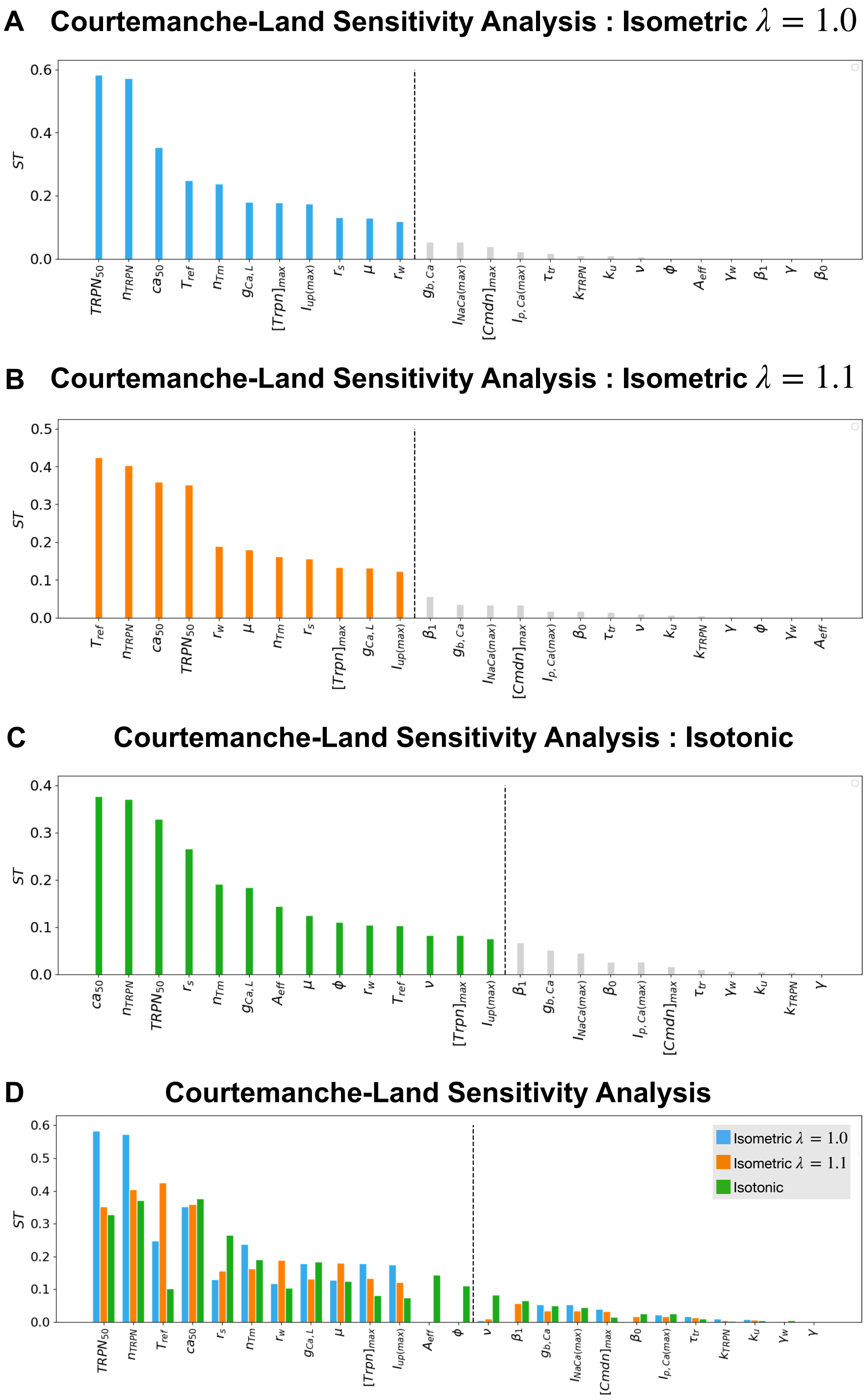

Fig 4. Courtemanche-Land parameter ranking. Parameters ranked according to the maximum total effect across all outputs for an isometric twitch with $\lambda = 1.0$ (**A**), an isometric twitch with λ=1.1 (**B**) and an isotonic twitch (**C**). Fig **D** shows the parameter ranking of all three simulation types combined.

History matching

In the section above, we used GPEs and GSA to identify which ionic and active tension parameters determine the active tension transient. In this section, we used HM to bound the important parameters to areas in the parameter space where the calcium and isometric tension are physiological. This also ensured that atrial tension transients were smaller than ventricular tension transients [12,13], and improved numerical stability in the whole heart simulations. The top section of Table 6 shows the target mean values μ and standard deviations σ for the active tension features we included in the HM, while the calcium features were constrained using the same values as above. There is a lack of experimental measurements of active tension in atrial myocytes. Consistent with the fact that the atria operate at lower pressure than the ventricles, we constrained the peak in isometric tension $\mathrm{T_{peak}}$ for the atria to be between 20 kPa and 40 kPa, as these values were at the lower end of peak tension ranges reported for ventricular myocytes [14–21]. Lower atrial active tension also limited atrial motion in the organ level electromechanics simulations, reducing the chances of instabilities. Similarly to the ToR-ORd-Land model and consistently with previous modelling studies [22], the rest tension was constrained between 0 and 1 kPa. To achieve these constraints in the HM with a miniumum threshold on the implausibility measure of 3, we set the mean and standard deviation μ and σ as in Table 6. Finally, the duration of the tension transient was set to 240±25 ms consistently with Land et al [22]. Constraining the duration of atrial contraction was necessary to ensure that atrial relaxation starts before the ventricles start contracting.

Table 6. History matching. The top section shows the mean μ and standard deviation σ for all model outputs, used as targets for the HM. The bottom section shows the settings and results for the three HM waves. From the left: threshold on the implausibility measure I_{th}, % of non-implausible points, mean and max implausibility measure, mean and max variance ratio between the GPEs and the data. *Waves where the first dataset was excluded from the GPE training. **Waves where only the datasets from the previous three waves are used for the GPE training. ***GPE not used.

**Model output**	μ	σ	**References**
$\mathrm{Ca_{diast}}$	0.1462 μM	0.01661 μM	[4, 6]
$\mathrm{Ca_{ampl}}$	0.5671 μM	0.2676 μM	[4, 6]
$\mathrm{dCa/dt_{max}}$	0.0876 μM/ms	0.00258 μM/ms	[3, 6]
RT90	413.0 ms	44.0 ms	[3, 4]
$\mathrm{T_{peak}}$	30.0 kPa	3.33 kPa	[14–21]
$\mathrm{T_{dur}}$	240.0 ms	25.0 ms	[3]
$\mathrm{T_{rest}}$	0.5 kPa	0.1667 kPa	[22]

Results

**Wave**	$I_{\mathbf{th}}$	**% NIMP**	**mean** I	**max** I	**mean** $V_{\mathbf{ratio}}$	**max** $V_{\mathbf{ratio}}$
**wave1**	3.5	24.2	5.4	59.6	116.57	2746.84
**wave2**	3.0	53.6	2.9	5.9	24.99	1289.11
**wave3**	3.0	71.1	2.7	6.1	8.59	775.99
**wave4***	3.0	86.1	2.5	6.0	4.25	294.35
**wave5***	3.0	63.7	3.1	6.6	1.14	13.09
**wave6****	3.0	86.2	3.0	5.8	0.79	12.51
**wave7****	3.0	76.1	3.0	10.1	0.27	4.35
**wave8****	3.0	91.5	2.9	4.5	0.23	3.32
**wave9****	3.0	94.5	2.9	4.5	0.18	2.66
**wave10*****	3.0	89.7	3.0	9.8	0.0	0.0

The default values and bounds for all parameters is provided in Table 4 (white rows). The default values for the calcium parameters was set to the center of the non-implausible region from the last HM wave on the Courtemanche model, while all other parameters were fixed at their default value listed in Table 4. The reference tension T_{ref} was limited between 80 kPa and 120 kPa. The bottom section of Table 6 summarises the results for the HM iterations. The initial GPEs were trained on 260 simulations, and the first wave was run with a threshold on the non-implausibility measure of 3.5. The threshold was then decreased to 3.0 for all other waves, and the GPE traning set was enriched with $\mathrm{N_{simul}}$=128 additional simulations. For the 4th and the 5th waves, the initial dataset was excluded from the training dataset for the GPEs, to import prediction accuracy within the non-implausible region. To improve accuracy even further and avoid training the GPEs with unphysiological samples, waves 6 to 9 were run only keeping the training datasets from the last three waves. The percentage on non-implausible points increased from 24.2% to 94.5%, and the mean implausibility

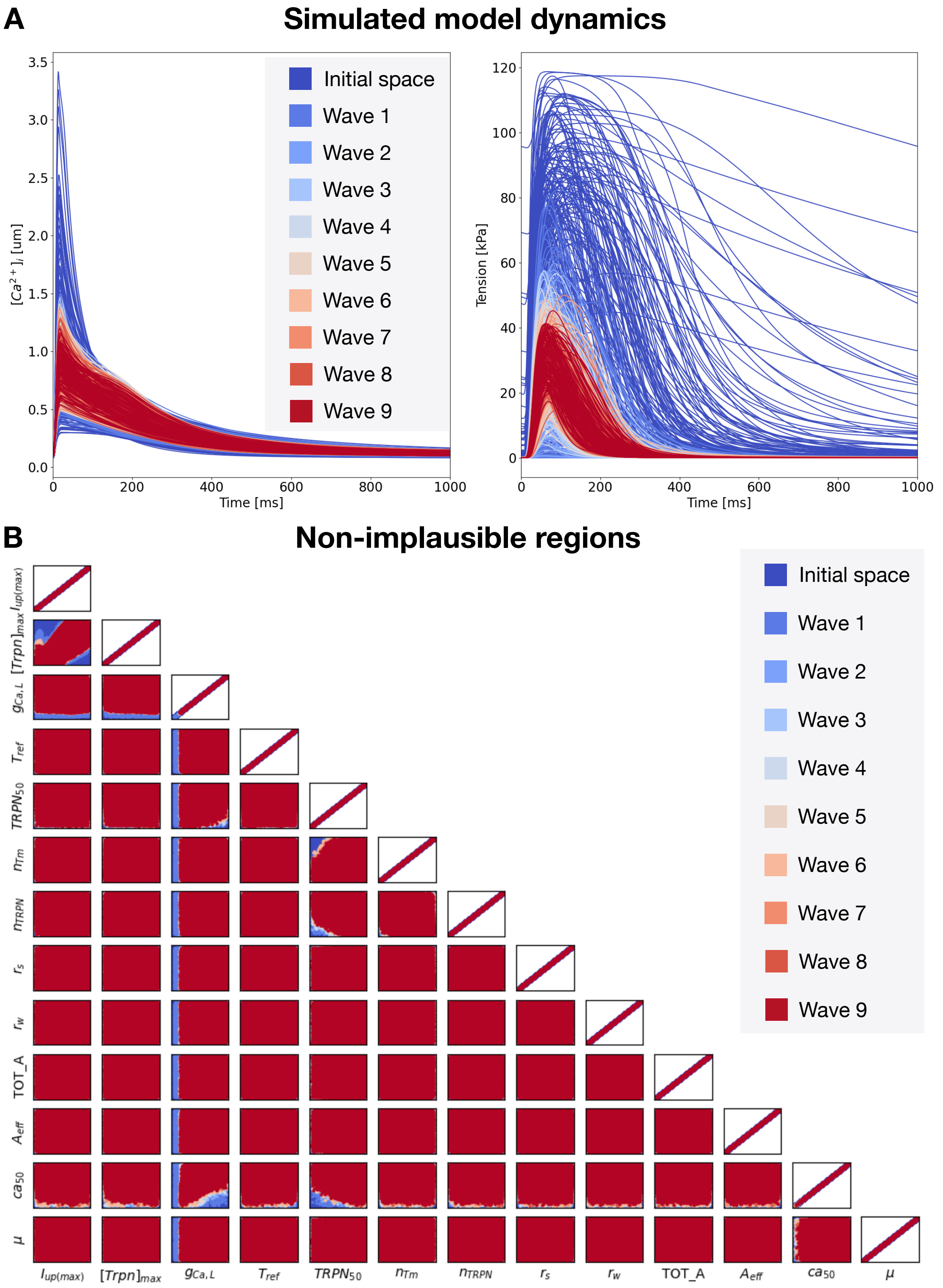

Fig 5. History matching. **A** Simulated calcium and tension transients with the initial samples (blue) to the samples extracted from the non-implausible region of each wave to refine the GPEs. **B** The non-implausible area is shown, starting from the initial test samples (blue) down to the non-implausible region for the last wave (red).

measure decreased from 5.4 to 2.9. Starting from very uncertain GPEs with a mean variance ratio of 116.57, indicating that the GPEs are more than a 100 times more uncertain than the experimental data, the variance ratio decreased to 0.18. By restricting the parameter space to the non-implausible area (Fig 5B, blue to red), the calcium and active tension transients used to train the GPEs go from unphysiological (Fig 5A, blue) to physiological (red).

Since the samples from the non-implausible area of the last HM wave on the Courtemanche-Land model will be used to run whole organ simulations, we need to ensure that the model behaves physiologically for all samples. This was ensured by running one last HM wave discarding the GPEs (e.g. using cell model evaluations rather than GPE predictions and setting the uncertainty of the prediction to 0). We ran 200,000 test samples with the cell model extracted from the non-implausible region of wave 9, evaluated the implausibility measure for all samples, and excluded those which did not provide physiological calcium and tension outputs.

Following initial tests in the whole heart simulations, we identified two further edge cases that led to numerical instabilities. Therefore, we ran the following additional checks on each sample before running whole organ simulations:

1. isometric twitch $\lambda = 1.1$ and basic cycle length of 1000 ms: $T_{\text{rest}}<2$ kPa to ensure that, even for non-zero strains, the rest tension was not too high

2. isometric twitch $\lambda = 1.2$ (maximum stretch allowed in the Land model) basic cycle length of 854 ms (based on clinical data): ensure that the ODEs system for the cell model did not become too stiff, causing the system to diverge. This is normally caused by the equation for CaTRPN, where the calcium sensitivity $ca_{50}(\lambda)$ is computed as a function of λ:

   $$ca_{50}(\lambda) = ca_{50} + \beta_1(\lambda - 1.0).$$

   To prevent this from happening for too many simulations, we scaled β_1 according to the value of ca_{50}:

   $$\beta_1 = \frac{\hat{\beta}_1}{\hat{ca}_{50}} ca_{50},$$

   where $\hat{ca}_{50}$=0.86 μM and $\hat{\beta}_1 = -2.4$ are the default values from the Land model adapted for the atria [3].

This ultimately provided us with N=148527 samples for the Courtemanche-Land model that could be used for simulations at the whole organ level.

References

1. Courtemanche M, Ramirez RJ, Nattel S. Ionic mechanisms underlying human atrial action potential properties: insights from a mathematical model. American Journal of Physiology-Heart and Circulatory Physiology. 1998;275(1):H301–H321.

2. Tanaami T, Ishida H, Seguchi H, Hirota Y, Kadono T, Genka C, et al. Difference in propagation of Ca2+ release in atrial and ventricular myocytes. The Japanese journal of physiology. 2005; p. 0504270003–0504270003.

3. Land S, Niederer SA. Influence of atrial contraction dynamics on cardiac function. Int J Numer Method Biomed Eng. 2018;34(3):e2931–e2931.

4. Coppini R, Ferrantini C, Yao L, Fan P, Del Lungo M, Stillitano F, et al. Late sodium current inhibition reverses electromechanical dysfunction in human hypertrophic cardiomyopathy. Circulation. 2013;127(5):575–584.

5. Brixius K, Pietsch M, Hoischen S, Muller-Ehmsen J, Schwinger RH. Effect of inotropic interventions on contraction and Ca2+ transients in the human heart. Journal of Applied Physiology. 1997;83(2):652–660.

6. Piacentino III V, Weber CR, Chen X, Weisser-Thomas J, Margulies KB, Bers DM, et al. Cellular basis of abnormal calcium transients of failing human ventricular myocytes. Circulation research. 2003;92(6):651–658.

7. Jung A, Gsell MA, Augustin CM, Plank G. An Integrated Workflow for Building Digital Twins of Cardiac Electromechanics—A Multi-Fidelity Approach for Personalising Active Mechanics. Mathematics. 2022;10(5):823.

8. Land S, Park-Holohan SJ, Smith NP, dos Remedios CG, Kentish JC, Niederer SA. A model of cardiac contraction based on novel measurements of tension development in human cardiomyocytes. J Mol Cell Cardiol. 2017;106:68–83.

9. Stuyvers BD, McCulloch AD, Guo J, Duff HJ, ter Keurs HE. Effect of stimulation rate, sarcomere length and Ca2+ on force generation by mouse cardiac muscle. The Journal of physiology. 2002;544(3):817–830.

10. Land S, Niederer SA. A spatially detailed model of isometric contraction based on competitive binding of troponin I explains cooperative interactions between tropomyosin and crossbridges. PLoS Computational Biology. 2015;11(8):e1004376.

11. Margara F, Wang ZJ, Levrero-Florencio F, Santiago A, Vázquez M, Bueno-Orovio A, et al. In-silico human electro-mechanical ventricular modelling and simulation for drug-induced pro-arrhythmia and inotropic risk assessment. Progress in biophysics and molecular biology. 2021;159:58–74.

12. Narolska N, Eiras S, Van Loon R, Boontje N, Zaremba R, Stooker W, et al. Myosin heavy chain composition and the economy of contraction in healthy and diseased human myocardium. Journal of Muscle Research & Cell Motility. 2005;26(1):39–48.

13. Ferrantini C, Coppini R, Scellini B, Ferrara C, Pioner JM, Mazzoni L, et al. R4496C RyR2 mutation impairs atrial and ventricular contractility. Journal of General Physiology. 2016;147(1):39–52.

14. Niederer S, Hunter P, Smith N. A quantitative analysis of cardiac myocyte relaxation: a simulation study. Biophysical journal. 2006;90(5):1697–1722.

15. Allen D, Kurihara S. The effects of muscle length on intracellular calcium transients in mammalian cardiac muscle. The Journal of physiology. 1982;327(1):79–94.

16. Kentish JC. Combined inhibitory actions of acidosis and phosphate on maximum force production in rat skinned cardiac muscle. Pflügers Archiv. 1991;419(3):310–318.

17. Hinken AC, McDonald KS. Inorganic phosphate speeds loaded shortening in rat skinned cardiac myocytes. American Journal of Physiology-Cell Physiology. 2004;287(2):C500–C507.

18. Wolska BM, Vijayan K, Arteaga GM, Konhilas JP, Phillips RM, Kim R, et al. Expression of slow skeletal troponin I in adult transgenic mouse heart muscle reduces the force decline observed during acidic conditions. The Journal of physiology. 2001;536(3):863–870.

19. Hibberd M, Jewell B. Calcium-and length-dependent force production in rat ventricular muscle. The Journal of physiology. 1982;329(1):527–540.

20. Ebus J, Papp Z, Zaremba R, Stienen G. Effects of MgATP on ATP utilization and force under normal and simulated ischaemic conditions in rat cardiac trabeculae. Pflügers Archiv. 2001;443(1):102–111.

21. Papp Z, Szabó Á, Barends JP, Stienen G. The mechanism of the force enhancement by MgADP under simulated ischaemic conditions in rat cardiac myocytes. The Journal of physiology. 2002;543(1):177–189.

22. Land S, Niederer SA, Aronsen JM, Espe EKS, Zhang L, Louch WE, et al. An analysis of deformation-dependent electromechanical coupling in the mouse heart. The Journal of physiology. 2012;590(18):4553–4569.
