# Supplementary material for: Cell to whole organ global sensitivity analysis on a four-chamber heart electromechanics model using Gaussian processes emulators
Source: PLoS Comput Biol. 2023 Jun 26;19(6):e1011257. doi: 10.1371/journal.pcbi.1011257 (PMC10328347; doi:10.1371/journal.pcbi.1011257)
Supplement: S4 File — We trained GPEs to predict the total atrial and ventricular activation times, and used them to run a GSA to identify important conduction parameters for simulated activation times. Then, we used HM to isolate areas in the parameter space where the total ventricular and atrial activation times were physiological. (PDF) [file pcbi.1011257.s004.pdf]

Electrophysiology global sensitivity analysis

We used Gaussian processes emulators (GPEs) to run a global sensitivity analysis (GSA) on the electrophysiology model at the tissue level. This aimed at excluding the unimportant parameters, in order to reduce the number of simulations needed for the GSA on the fully coupled framework. We then used Bayesian history matching (HM) to constrain the parameters to areas where the total atrial and ventricular activation times were within physiological values.

Gaussian processes emulators and sensitivity analysis

We sampled the parameter space with a Latin hypercybe design with N=180 samples. Table 1 shows the parameters we considered and the ranges used to bound each parameter, together with references for these values. For every sample, the Eikonal model was run to compute activation times on the four-chamber heart model. We trained GPEs to predict the following outputs:

1. total atrial activation time ($\mathrm{TAT_A}$)
2. total ventricular activation time ($\mathrm{TAT_V}$).

Table 2 reports the R^2 score and the ISE for each cross-validation split to show that the GPEs provide an accurate prediction for both features.

Table 1. Electrophysiology simulator parameters. The first two columns show the parameter symbol and its meaning. The third and the fourth contain the ranges used to sample the parameter space and the references for these values.

Symbol	Meaning	Range	Reference
$\mathrm{CV_{f,v}}$	CV in the fibre direction of the ventricles	[0.38,0.8]	[1–3]
$\mathrm{k_{ft,v}}$	Anisotropy ratio of the ventricles	[0.2,0.5]	[1, 3–6]
$\mathrm{k_{FEC}}$	Scaling factor for the CV of the fast endocardial conduction layer	[1.2,8.4]	[1, 7, 8]
$\mathrm{CV_{f,a}}$	CV in the fibre direction of the atria	[0.3,1.03]	[2, 9–12]
$\mathrm{k_{ft,a}}$	Anisotropy ratio of the atria	[0.2,0.5]	[1, 3–6]
$\mathrm{k_{BB}}$	Scaling factor for the CV of the Bachmann bundle	[1.0,5.7]	[10, 12, 13]

Table 2. GPEs performance. R^2 score and ISE for every split of a 5-fold cross-validation, reported for each output.

Model output	Meaning	Metric	fold-1	fold-2	fold-3	fold-4	fold-5	Mean
$\mathrm{TAT_A}$	Total atrial activation time	R^2	0.9998	0.9995	0.9992	0.9995	0.9989	0.9994
		ISE	97.22	88.89	88.89	100.00	91.67	93.33
$\mathrm{TAT_V}$	Total ventricular activation time	R^2	0.9996	0.9996	0.9997	0.9998	0.9993	0.9996
		ISE	88.89	97.22	97.22	94.44	94.44	94.44

We used the GPEs to run a GSA and identify important parameters. A Saltelli sampling was generated using a base Sobol sequence with $\mathrm{N_{base}}$=1000 samples and the GPEs were evaluated to predict model outputs and compute the total order effects. Fig 1A shows the heatmap of the total effect of the parameters (x-axis) over the outputs (y-axis). The fibre conduction velocity (CV) of the ventricles ($CV_{\mathrm{f,a}}$) and the ratio of the CV in the fast endocardial conduction (k_{FEC}) layer had the highest impact on the total activation time of the ventricles, while the anisotropy ratio of the ventricles ($k_{\mathrm{ft,v}}$) had negligible effect on the outputs. Similarly, the total activation time of the atria was affected by the fibre CV of the atria ($CV_{\mathrm{f,a}}$) and the ratio of CV in the Bachmann bundle area (k_{BB}), but not by the anisotropy ratio of the atria ($k_{\mathrm{ft,a}}$). The parameter ranking

in Fig 1B, performed according to the maximum total effect across all output, shows that the CV in the fibre direction for the atria and the ventricles, and the CV ratio in the FEC and in the Bachmann bundle are the most important parameters. Normalising the maximum total effects to sum up to 1, e.g. 100% of output variance, shows that these four parameters are enough to explain >90% of outputs variance (orange bars). Therefore, the anisotropy ratios can be excluded from the analysis.

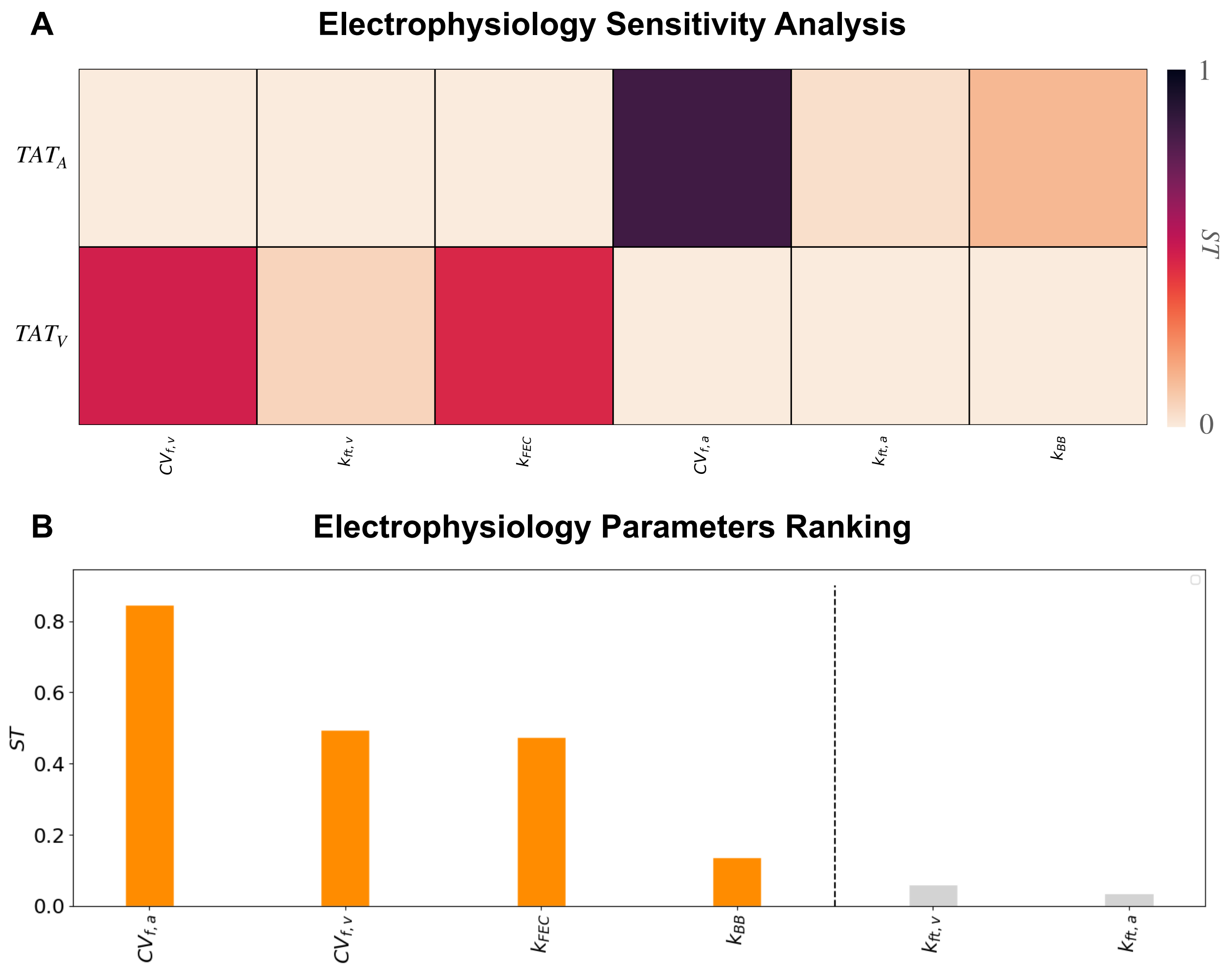

Fig 1. Sensitivity analysis results. A Heatmap of the total effect of the parameters (x-axis) on the outputs (y-axis). **B** Barplot of the maximum total effect of each parameter over all outputs. The parameters are ranked from most to least important. The dashed line separates important (orange) and unimportant (gray) parameters.

History matching

In the previous section, we used GPEs and GSA to identify important parameters in the electrophysiology model, and exclude the unimportant ones. In this section, we aim to use GPEs and HM to identify areas in the parameter space where the important parameters provide physiological model outputs. Details about the HM procedure are provided in the manuscript.

The ranges for the important parameters were set as in Table 1, while the anisotropy ratio for atria and ventricles were fixed to 0.4 [1]. The mean target value μ for the total ventricular activation time $\mathrm{TAT_V}$ was set to the QRS duration of the patient from a 12-lead ECG, measured at 200 ms. However, we did not have information about total atrial activation time $\mathrm{TAT_A}$ or the P-wave duration from the patient data. Literature data for P-wave duration in atrial fibrillation patients in sinus rhythm range from 110 ms to 160 ms [14–19].

The mean target value for TAT_A was therefore set to 135 ms, with a standard deviation σ of 8 ms. These values together with the 'three sigma rule' [20] as the minimum threshold we impose on the implausibility measure I mean that we are constraining TAT_A to be between 111 ms and 159 ms, consistent with literature values. The standard deviation σ for the TAT_V, which was not available from the clinical data, was set to 10 ms, equivalent to a $\sim$5% uncertainty, similarly to literature uncertainty for TAT_A.

Table 3. History matching. The top section shows the mean μ and standard deviation σ for the total activation time for the atria and the ventricles, used as targets for the HM. The bottom section shows the settings and results for the three HM waves. From the left: threshold on the implausibility measure I_{th}, % of non-implausible points, mean and max implausibility measure, mean and max variance ratio between the GPEs and the data.

**Model output**	μ		σ		**References**	
TAT_A	135 ms		8 ms		[14–19]	
TAT_V	200 ms		10 ms		Clinical data	
**Results**						
**Wave**	I_{th}	**% NIMP**	**mean** I	**max** I	**mean** V_{ratio}	**max** V_{ratio}
**wave 1**	3.5	11.0%	12.8	49.2	0.05	0.72
**wave 2**	3.0	84.5%	2.0	3.6	0.03	0.21
**wave 3**	3.0	99.7%	1.8	3.0	0.02	0.18

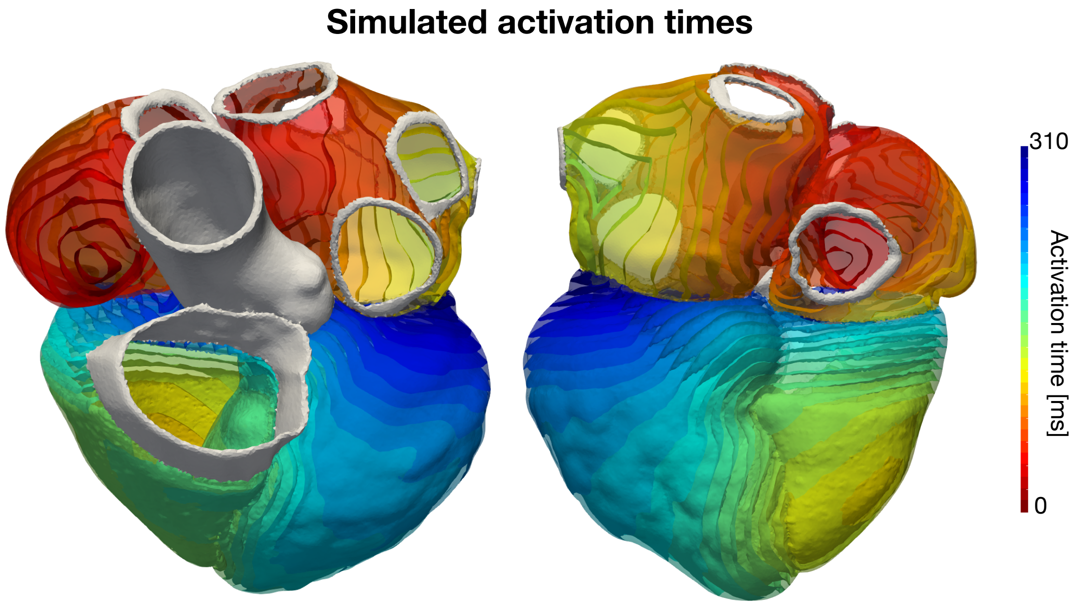

Fig 2. Simulated activation. Activation times simulated for one of the samples extracted from the non-implausible area from the last history matching wave.

Table 3 summarises the results of the HM. We ran three waves, starting with a 3.5 threshold on the implausibility measure, and then decreased it to 3.0 for the next two waves. The initial parameter space was sampled with N=80 points using a Latin hypercube design, and these samples were used to train the initial GPEs. Then, at each wave, the GPEs were re-trained with N_{simul}=32 additional samples extracted from the non-implausible region. At every wave, the percentage of non-implausible points increases (from 11% to 99.7%), and the mean and maximum implausibility measure decrease, indicating that the parameter space is restricted to areas where the outputs are within the target ranges (Fig 3). Furthermore, the variance ratio between the GPEs and the data decreases (Table 3, last column), indicating that the GPE predictions become less uncertain compared to the uncertainty on the data. Fig 2 shows simulated activation times for one of the samples extracted from the non-implausible area of the last wave. This simulation resulted in a total atrial and ventricular activation times of 145.6 ms and 211.8 ms, which are within the target ranges for the HM.

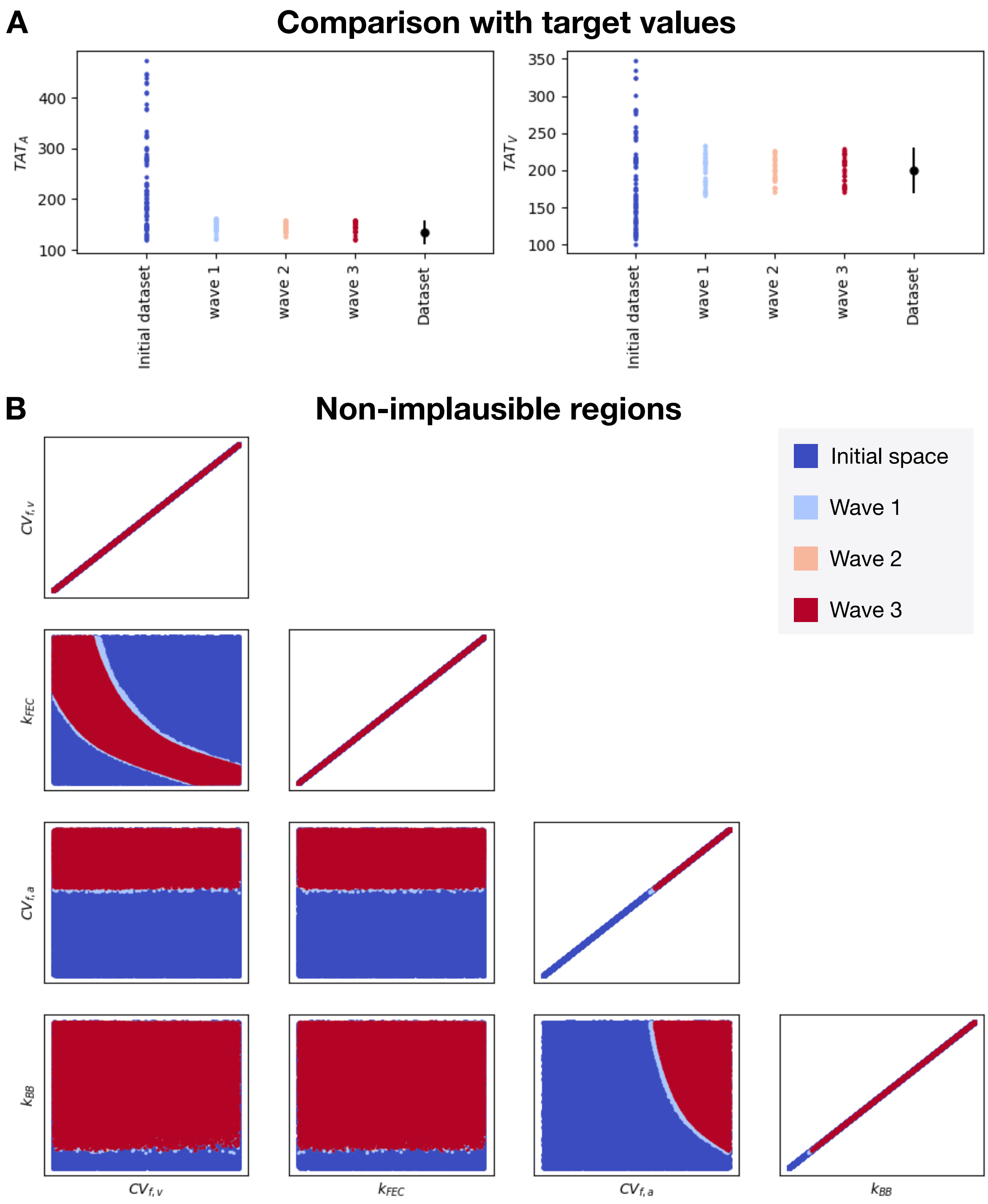

Fig 3. History matching. A The target output values ($\mu \pm 3\sigma$, black) are compared to the initial simulation outputs (blue) and to the simulation outputs used to re-train the GPEs at every wave. **B** The non-implausible area is shown, starting from the initial test samples (blue) down to the non-implausible region for the last wave (red).

References

1. Lee AW, Nguyen UC, Razeghi O, Gould J, Sidhu BS, Sieniewicz B, et al. A rule-based method for predicting the electrical activation of the heart with cardiac resynchronization therapy from non-invasive clinical data. Medical Image Analysis. 2019;57:197–213.

2. Draper MH, Mya-Tu M. A comparison of the conduction velocity in cardiac tissues of various mammals. Quarterly journal of experimental physiology and cognate medical sciences. 1959;44(1):91–109.

3. Roberts DE, Hersh LT, Scher AM. Influence of cardiac fiber orientation on wavefront voltage, conduction velocity, and tissue resistivity in the dog. Circulation Research. 1979;44(5):701–712.

4. Saffitz JE, Kanter HL, Green KG, Tolley TK, Beyer EC. Tissue-specific determinants of anisotropic conduction velocity in canine atrial and ventricular myocardium. Circulation research. 1994;74(6):1065–1070.

5. Clerc L. Directional differences of impulse spread in trabecular muscle from mammalian heart. The Journal of physiology. 1976;255(2):335–346.

6. Roth BJ. Electrical conductivity values used with the bidomain model of cardiac tissue. IEEE Transactions on Biomedical Engineering. 1997;44(4):326–328.

7. Myerburg RJ, Gelband H, Nilsson K, Castellanos A, Morales AR, Bassett AL. The role of canine superficial ventricular muscle fibers in endocardial impulse distribution. Circulation research. 1978;42(1):27–35.

8. Ono N, Yamaguchi T, Ishikawa H, Arakawa M, Takahashi N, Saikawa T, et al. Morphological varieties of the Purkinje fiber network in mammalian hearts, as revealed by light and electron microscopy. Archives of histology and cytology. 2009;72(3):139–149.

9. van Campenhout MJ, Yaksh A, Kik C, de Jaegere PP, Ho SY, Allessie MA, et al. Bachmann's bundle: a key player in the development of atrial fibrillation? Circulation: Arrhythmia and Electrophysiology. 2013;6(5):1041–1046.

10. Wagner ML, Lazzara R, Weiss RM, Hoffman BF. Specialized conducting fibers in the interatrial band. Circulation Research. 1966;18(5):502–518.

11. Hansson A, Holm M, Blomström P, Johansson R, Lührs C, Brandt J, et al. Right atrial free wall conduction velocity and degree of anisotropy in patients with stable sinus rhythm studied during open heart surgery. European heart journal. 1998;19(2):293–300.

12. Hayashi H, Lux RL, Wyatt RF, Burgess M, Abildskov J. Relation of canine atrial activation sequence to anatomic landmarks. American Journal of Physiology-Heart and Circulatory Physiology. 1982;242(3):H421–H428.

13. Spach MS, Dolber PC, Heidlage JF. Interaction of inhomogeneities of repolarization with anisotropic propagation in dog atria. A mechanism for both preventing and initiating reentry. Circulation research. 1989;65(6):1612–1631.

14. Karacop E, Enhos A, Bakhshaliyev N, Ozdemir R. P Wave Duration/P Wave Voltage Ratio Plays a Promising Role in the Prediction of Atrial Fibrillation: A New Player in the Game. Cardiology research and practice. 2021;2021.

15. Nielsen PB, Larsen TB, Skjøth F, Gorst-Rasmussen A, Rasmussen LH, Lip GY. Restarting anticoagulant treatment after intracranial hemorrhage in patients with atrial fibrillation and the impact on recurrent stroke, mortality, and bleeding: a nationwide cohort study. Circulation. 2015;132(6):517–525.

16. Pérez FJ, Schubert CM, Parvez B, Pathak V, Ellenbogen KA, Wood MA. Long-term outcomes after catheter ablation of cavo-tricuspid isthmus dependent atrial flutter: a meta-analysis. Circulation: Arrhythmia and Electrophysiology. 2009;2(4):393–401.

17. Kreimer F, Aweimer A, Pflaumbaum A, Mügge A, Gotzmann M. Impact of P-wave indices in prediction of atrial fibrillation—Insight from loop recorder analysis. Annals of Noninvasive Electrocardiology. 2021;26(5):e12854.

18. Dilaveris PE, Gialafos EJ, Sideris SK, Theopistou AM, Andrikopoulos GK, Kyriakidis M, et al. Simple electrocardiographic markers for the prediction of paroxysmal idiopathic atrial fibrillation. American heart journal. 1998;135(5):733–738.

19. Aytemir K, ÖZER N, Atalar E, Sade E, AKSÖYEK S, ÖVÜNÇ K, et al. P wave dispersion on 12-lead electrocardiography in patients with paroxysmal atrial fibrillation. Pacing and Clinical Electrophysiology. 2000;23(7):1109–1112.

20. Pukelsheim F. The three sigma rule. The American Statistician. 1994;48(2):88–91.
