# Supplementary material for: Cell to whole organ global sensitivity analysis on a four-chamber heart electromechanics model using Gaussian processes emulators
Source: PLoS Comput Biol. 2023 Jun 26;19(6):e1011257. doi: 10.1371/journal.pcbi.1011257 (PMC10328347; doi:10.1371/journal.pcbi.1011257)
Supplement: S5 File — We trained GPEs to predict the maximum volumes for the four chambers and ventricular and atrial fibres strains during a passive inflation, and used them to run a GSA to identify important stiffness parameters. (PDF) [file pcbi.1011257.s005.pdf]

Passive mechanics sensitivity analysis

We used Gaussian processes emulators (GPEs) to run a global sensitivity analysis (GSA) on the passive mechanics parameters, aiming to exclude the unimportant stiffness parameters from the fully-coupled simulator. We ran a passive inflation of all four chambers constrained by the pericardium (Fig 1). The left and the right chambers were inflated up to a pressure of 9 mmHg and 5.6 mmHg, respectively, consistent with left ventricular (LV) and right ventricular (RV) end-diastolic pressure measurements in atrial fibrillation patients in sinus rhythm [1]. Table 1 summarises the parameters we considered in the analysis. We included all passive stiffness parameters for the atria (a_{A}, $b_{\mathrm{f,A}}$, $b_{\mathrm{ft,A}}$, $b_{\mathrm{t,A}}$) and the ventricles (a_{V}, $b_{\mathrm{f,V}}$, $b_{\mathrm{ft,V}}$, $b_{\mathrm{t,V}}$), and the stiffness of the normal springs for the effect of the pericardium pericardium ($\mathrm{k_{peri}}$). The default stiffness values for atria and ventricles were based on Nasopoulou et al [2], while the range was set between 1/4 to 4 times the default values to account for the high uncertainty in myocardium stiffness reported in the literature. For each passive inflation, we computed the following outputs:

1. inflated LV volume ($V_{\mathrm{LV}}^{\max}$)
2. inflated RV volume ($V_{\mathrm{RV}}^{\max}$)
3. inflated left atrium (LA) volume ($V_{\mathrm{LA}}^{\max}$)
4. inflated right atrium (RA) volume ($V_{\mathrm{RA}}^{\max}$)
5. mean inflated ventricular fibre strain ($\overline{E_{\mathrm{ff,V}}}$)
6. mean inflated atrial fibre strain ($\overline{E_{\mathrm{ff,A}}}$).

Table 1. Simulator parameters (continued).

Symbol	Default	GSA	Meaning	Reference
a_{V}	1.7	[*1/4,*4]	Bulk myocardium stiffness	[2]
$b_{\mathrm{f,V}}$	8.0	[*1/4,*4]	Stiffness in the fibre direction	[2]
$b_{\mathrm{ft,V}}$	4.0	[*1/4,*4]	Stiffness in the fibre-transverse shear planes	[2]
$b_{\mathrm{t,V}}$	3.0	[*1/4,*4]	Stiffness in the transverse plane	[2]
a_{A}	1.7	[*1/4,*4]	Bulk myocardium stiffness	[2]
$b_{\mathrm{f,A}}$	8.0	[*1/4,*4]	Stiffness in the fibre direction	[2]
$b_{\mathrm{ft,A}}$	4.0	[*1/4,*4]	Stiffness in the fibre-transverse shear planes	[2]
$b_{\mathrm{t,A}}$	3.0	[*1/4,*4]	Stiffness in the transverse plane	[2]
$\mathrm{k_{peri}}$	0.001	[*1/4,*4]	Pericardium spring stiffness	-

The parameter space was sampled with 180 points using a Latin hypercube design. Of these 180 simulations, 176 ran successfully. These samples were used to train GPEs (one per output). GPEs performance scores are provided in Table 2. For all outputs, the mean R^2 and ISE are above 0.95 and 89.0, respectively, showing that the GPEs provide an accurate prediction of the model outputs. We used the GPEs to run a GSA and identify important parameters. A Saltelli sampling was generated using a base Sobol sequence with $\mathrm{N_{base}}$=1000 samples and the GPEs were evaluated to predict model outputs and compute the total order effects, in order to identify important stiffness parameters. Fig 2A shows the heatmap of the total effect of the parameters (x-axis) over the outputs (y-axis). The inflated volume of both ventricles ($V_{\mathrm{LV}}^{\max}$and $\mathrm{V^{max}}_{\mathrm{RV}}$) were affected the most by the bulk stiffness of the ventricles a_{V}, followed by the transverse stiffness ($b_{\mathrm{t,V}}$) and fibre stiffness ($b_{\mathrm{f,V}}$) of ventricular myocardium. Similarly, atrial bulk stiffness (a_{A}), fibre stiffness ($b_{\mathrm{f,A}}$) and transverse stiffness ($b_{\mathrm{t,A}}$) determined the inflated LA and RA volumes. The stiffness of the pericardium ($\mathrm{k_{peri}}$) affected to a different extent the inflated volumes of all four chambers, with higher effects on the RA

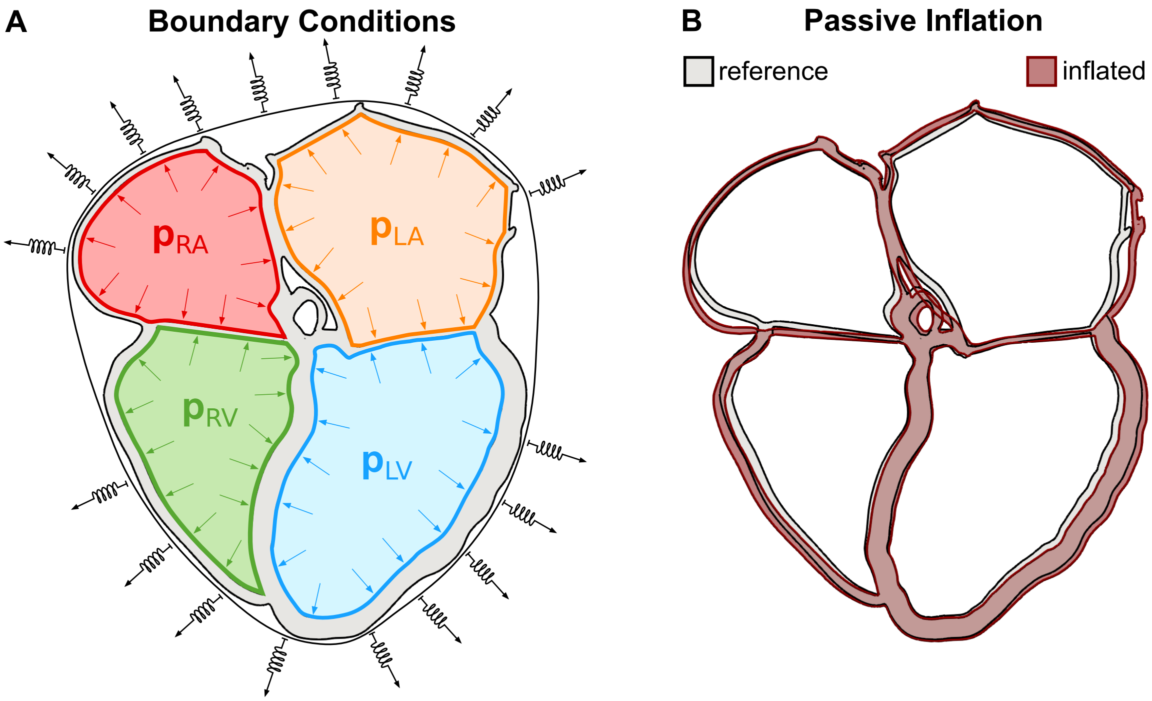

Fig 1. A Boundary conditions. The passive inflation was by inflating the atria and the ventricles simultaneously, while applying spring boundary conditions for the pericardium, as described in the main manuscript. **B Passive inflation simulation.** Example inflated geometry (red) compared to the reference geometry (grey).

and the RV. The effects on the mean fibre strains of ventricles and atria ($\overline{E_{\mathrm{ff,V}}}$ and $\overline{E_{\mathrm{ff,A}}}$) followed a similar trend to the inflated volumes, although the fibre stiffness parameters ($b_{\mathrm{f,V}}$ and $b_{\mathrm{f,A}}$) played a more important role in the strains compared to the volumes. The parameter ranking in Fig 2B , performed according to the maximum total effect across all outputs, shows that the bulk and the transverse stiffness parameters for both atria and ventricles, the stiffness of the pericardium and the atrial fibre stiffness are the most important parameters. When normalising the maximum total effects to sum up to 1 (e.g. 100% of explained variance), the figure shows that these parameters are enough to explain >90% of the outputs variance. Therefore, the fibre stiffness of the ventricles and the shear stiffness parameters ($b_{\mathrm{ft,V}}$ and $b_{\mathrm{ft,A}}$) can be excluded from the analysis.

Table 2. GPEs performance. R^2 score and ISE for every split of a 5-fold cross-validation, reported for each output.

Model output	Meaning	Metric	fold-1	fold-2	fold-3	fold-4	fold-5	Mean
$V_{\mathrm{LV}}^{\mathrm{max}}$	Max LV volume	R^2	0.9826	0.9872	0.9895	0.9492	0.9960	0.9809
		ISE	86.11	91.43	88.57	82.86	100.00	89.79
$V_{\mathrm{RV}}^{\mathrm{max}}$	Max RV volume	R^2	0.9541	0.9676	0.9766	0.9480	0.9880	0.9669
		ISE	86.11	91.43	85.71	88.57	100.00	90.37
$V_{\mathrm{LA}}^{\mathrm{max}}$	Max LA volume	R^2	0.9897	0.9899	0.9872	0.9872	0.9814	0.9871
		ISE	94.44	94.29	94.29	88.57	82.86	90.89
$V_{\mathrm{RA}}^{\mathrm{max}}$	Max RA volume	R^2	0.9784	0.9642	0.9906	0.9792	0.9491	0.9723
		ISE	91.67	91.43	97.14	94.29	85.71	92.05
$\overline{E_{\mathrm{ff,V}}}$	Mean ventricular fibre strain	R^2	0.9861	0.9873	0.9907	0.9510	0.9952	0.9821
		ISE	86.11	88.57	88.57	80.00	100.00	88.65
$\overline{E_{\mathrm{ff,A}}}$	Mean atrial fibre strain	R^2	0.9911	0.9930	0.9947	0.9920	0.9824	0.9906
		ISE	94.44	97.14	97.14	80.00	80.00	89.75

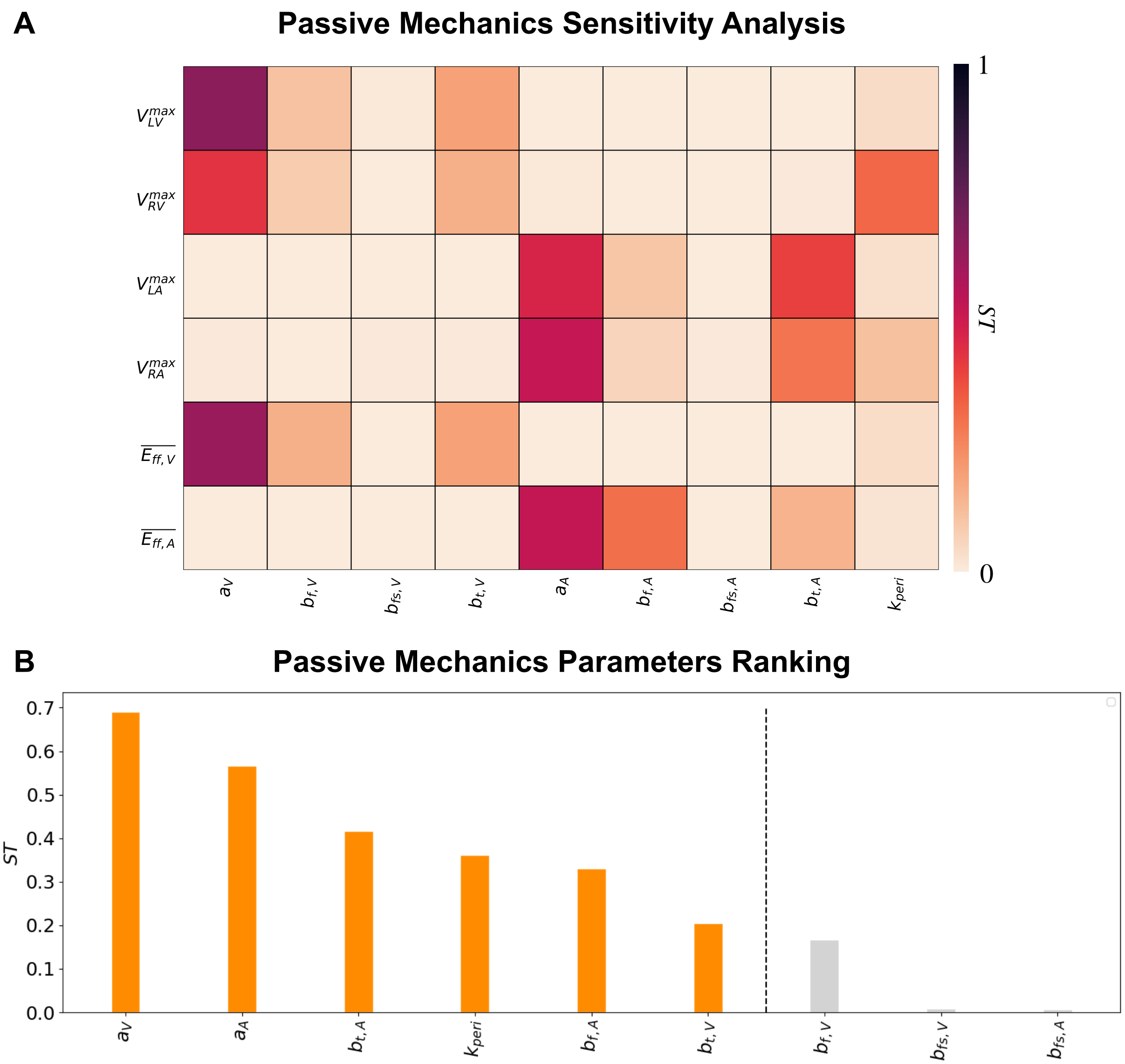

Fig 2. Sensitivity analysis results. A Heatmap of the total effect of the parameters (x-axis) on the outputs (y-axis). **B** Barplot of the maximum total effect of each parameter over all outputs. The parameters are ranked from most to least important. The dashed line separates important (orange) and unimportant (gray) parameters.

References

1. Alboni P, SCARFŒ S, Fuca G, Paparella N, Yannacopulu P. Hemodynamics of idiopathic paroxysmal atrial fibrillation. Pacing and Clinical Electrophysiology. 1995;18(5):980–985.

2. Nasopoulou A, Shetty A, Lee J, Nordsletten D, Rinaldi CA, Lamata P, et al. Improved identifiability of myocardial material parameters by an energy-based cost function. Biomech Model Mechanobiol. 2017;16(3):971–988.
