# Supplementary material for: Cell to whole organ global sensitivity analysis on a four-chamber heart electromechanics model using Gaussian processes emulators
Source: PLoS Comput Biol. 2023 Jun 26;19(6):e1011257. doi: 10.1371/journal.pcbi.1011257 (PMC10328347; doi:10.1371/journal.pcbi.1011257)
Supplement: S6 File — We trained GPEs to predict a wide range of pressure and volume output features for all four chambers simulated with CircAdapt, and used them to run a GSA to identify important circulatory parameters for pressure and volume dynamics. (PDF) [file pcbi.1011257.s006.pdf]

CircAdapt sensitivity analysis

To identify the most important parameters of the circulatory system, we ran a global sensitivity analysis (GSA) on the CircAdapt standalone model, defined by a set of ordinary differential equations (ODEs) [1]. In addition to the parameters determining the circulatory system (Table 1), we considered the CircAdapt parameters for the atrioventricular delay ($\mathrm{AV_{delay}}$), ventricular and atrial active ($\sigma_V^{\mathrm{act,max}}$ and $\sigma_A^{\mathrm{act,max}}$) and passive ($\sigma_V^{\mathrm{pas,max}}$ and $\sigma_A^{\mathrm{pas,max}}$) stresses in CircAdapt. Fig 1 shows a schematic of the parameters for passive and active atrial and ventricular mechanics in the fully-coupled model vs standalone CircAdapt. With this analysis, we aimed to identify which parameters of the circulatory system are important for the pressure and volume dynamics of all four chambers, relative to the active and passive stress parameters included in the three-dimensional electromechanics simulator.

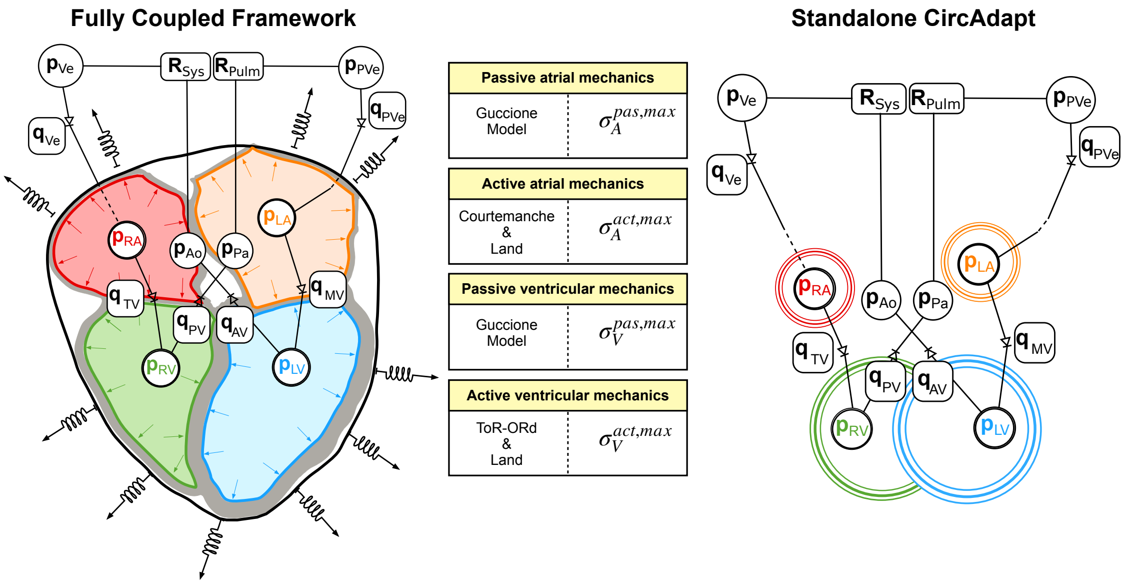

Fig 1. Schematic of the fully coupled simulator vs standalone CircAdapt.

Table 1 summarises all CircAdapt parameters considered in this analysis, together with their default value from [1]. For GPE training and the GSA, we considered a parameter range of ±25% from the default value. The parameter space was sampled with a latin hypercube design with N=630 points. For each parameter combination, we ran the standalone CircAdapt code for 100 beats with a basic cycle length of 850 ms to reach a steady state, and we computed the following model outputs for the left and the right ventricles (LV and RV), and for the left and the right atrium (LA and RA) from the pressure and volume dynamics during the last beat (see Fig 2):

1. LV/RV end-diastolic volume ($\mathrm{EDV_{LV}}$ and $\mathrm{EDV_{RV}}$)
2. LV/RV end-diastolic pressure ($\mathrm{EDP_{LV}}$ and $\mathrm{EDP_{RV}}$)
3. LV/RV pressure at the end of the iso-volumic contraction phase ($p_{\mathrm{LV}}^{\mathrm{IVC}}$ and $p_{\mathrm{RV}}^{\mathrm{IVC}}$)
4. LV/RV systolic peak in pressure ($p_{\mathrm{LV}}^{\mathrm{max}}$ and $p_{\mathrm{RV}}^{\mathrm{max}}$)
5. LV/RV end-systolic volume ($\mathrm{ESV_{LV}}$ and $\mathrm{ESV_{RV}}$)

Table 1. Circadapt model parameters.

Symbol	Default	GSA range	Meaning	Reference
		**Chambers**		
$\sigma_V^{\text{act,max}}$	120000.0	±25%	Ventricular maximum isometric active stress	[1, 2]
$\sigma_A^{\text{act,max}}$	84000.0	±25%	Atrial maximum isometric active stress	[1, 2]
$\sigma_V^{\text{pas,max}}$	22000.0	±25%	Ventricular maximum passive stress	[1, 2]
$\sigma_A^{\text{pas,max}}$	50000.0	±25%	Atrial maximum passive stress	[1, 2]
		**Electrophysiology**		
AV_{delay}	160.0	±25%	atrioventricular delay	[1, 2]
		**Tubes**		
p^0_{Ao}	1.0	±25%	Scaling factor for reference aortic pressure	[1, 2]
p^0_{Pa}	1.0	±25%	Scaling factor for reference pulmonary arterial pressure	[1, 2]
p^0_{Ve}	1.0	±25%	Scaling factor for reference systemic veins pressure	[1, 2]
p^0_{Pve}	1.0	±25%	Scaling factor for reference pulmonary veins pressure	[1, 2]
l_{Ao}	400.1115	±25%	Length of the aorta	[1, 2]
l_{Pa}	200.05575	±25%	Length of the pulmonary artery	[1, 2]
l_{Ve}	400.1115	±25%	Length of the systemic veins	[1, 2]
l_{Pve}	200.05575	±25%	Length of the pulmonary veins	[1, 2]
k_{Ao}	8.0	±25%	Stiffness of the aorta	[1, 2]
k_{Pa}	8.0	±25%	Stiffness of the pulmonary artery	[1, 2]
k_{Ve}	10.0	±25%	Stiffness of the veins	[1, 2]
k_{Pve}	10.0	±25%	Stiffness of the pulmonary veins	[1, 2]
		**Systemic Circulation**		
$\Delta p^{\text{ref}}_{\text{sys}}$	90.007389	±25%	Reference pressure drop across the systemic circulation	[1, 2]
$\Delta p^{\text{ref}}_{\text{pulm}}$	11.250924	±25%	Reference pressure drop across the pulmonary circulation	[1, 2]
R_{pulm}	1.0	±25%	Systemic resistance scaling factor	[1, 2]
R_{pulm}	1.0	±25%	Pulmonary resistance scaling factor	[1, 2]

6. LV/RV pressure at the end of ejection ($p^{\text{ej}}_{\text{LV}}$ and $p^{\text{ej}}_{\text{RV}}$)
7. LA/RA end-diastolic volume during the a-wave (EDV^a_{LA} and EDV^a_{RA})
8. LA/RA peak pressure during the a-wave ($p^{\text{max,a}}_{\text{LA}}$ and $p^{\text{max,a}}_{\text{RA}}$)
9. LA/RA end-systolic volume during the a-wave (ESV^a_{LA} and ESV^a_{RA})
10. LA/RA end-diastolic volume during the v-wave (EDV^v_{LA} and EDV^v_{RA})
11. LA/RA peak pressure during the v-wave ($p^{\text{max,v}}_{\text{LA}}$ and $p^{\text{max,v}}_{\text{RA}}$)
12. LA/RA end-systolic volume during the v-wave (ESV^v_{LA} and ESV^v_{RA})
13. LA/RA minimum, pressure during the v-wave ($p^{\text{min,v}}_{\text{LA}}$ and $p^{\text{min,v}}_{\text{RA}}$)

For every output listed above, we trained a GPE. GPE performance is summarized in Table 2, where the average R^2 and the ISE scores are provided, together with the metrics for every split in a 5-fold cross-validation. For all outputs, the average R^2 and ISE scores are above 0.93 and 92.0, meaning the the GPEs provide an accurate prediction for all the outputs. A Saltelli sampling was constructed with a Sobol base sequence with N_{base}=1000 samples, and the GPEs were evaluated to compute the total order effects on the outputs and identify the most important parameters for the pressure and volume features of all four chambers. Fig 3A shows the heatmap of the total effect of the parameters (x-axis) over the outputs (y-axis). The parameters for the systemic and the pulmonary peripheral circulations affected most of the outputs. The systemic resistance (R_{sys}) and pressure drop (Δp^{ref}_{sys}) influenced the end-diastolic volume and pressure of both ventricles, as well as their systolic peak in pressure. They also significantly affected all outputs for the left and the right atrium, although the end-systolic volume and the minimum pressure during the v-wave were affected to a lesser extent. The pulmonary peripheral resistance (R_{pulm}) and pressure drop (Δp^{ref}_{pulm}) determined the end-systolic volume and the pressure at the end of ejection of the right ventricle. The active stress of the ventricles affected the end-systolic volume of both ventricles, and the pressure and volume dynamics of the atria during the v-wave due to atrioventricular interaction. Similarly, atrial active stress impacted atrial dynamics during the a-wave. Atrioventricular interaction was also determined by the AV_{delay}, which affected

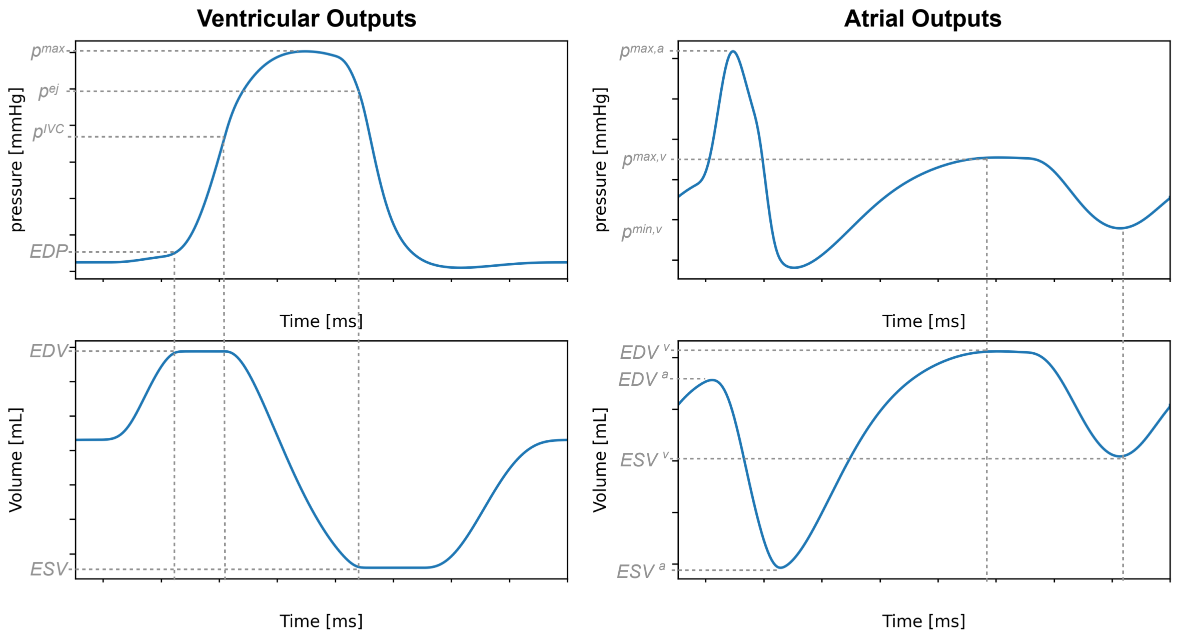

Fig 2. CircAdapt model outputs. Pressure (top) and volume (bottom) time traces for the left ventricle (left) and the left atrium (right), annotated with the outputs used to train the GPEs.

the end-diastolic pressure and the pressure at the end of the iso-volumic contraction of both ventricles, as well as the end-systolic and the end-diastolic volumes of the right atrium during the a-wave. Finally, the aortic stiffness (k_{Ao}) and length (l_{Ao}) affected the left ventricular pressure at the end of ejection.

The parameter ranking in Figure 3B, where the parameters were divided in important and non-important as explained in the main manuscript, shows that the systemic and pulmonary peripheral resistance parameters are the most important for the circulatory system, together with the aortic stiffness and length. The active stress of the atria and ventricles, and the atrioventricular delay are are needed to explain >90% of outputs variance, but these parameters are replaced by more detailed models in the fully coupled framework (Fig 1). Since the systemic resistance (R_{sys}) and pressure drop (Δp_{sys}^{ref}) affect the same outputs to the same extents, we fixed the pressure drop and varied only the resistance. For the same reason, in the analysis on the fully coupled framework, we fixed the pulmonary pressure drop and varied the pulmonary resistance.

Table 2. GPEs performance ToR-ORd (ventricular outputs). R^2 score and ISE for every split of a 5-fold cross-validation, reported for each output.

Model output	Meaning	Metric	fold-1	fold-2	fold-3	fold-4	fold-5	Mean
**Left ventricle outputs**								
$\mathrm{EDV}_{\mathrm{LV}}$	LV end-diastolic volume	R^2	0.9996	0.9995	0.9993	0.9994	0.9994	0.9994
		ISE	96.83	93.65	94.44	92.06	93.65	94.13
$\mathrm{EDP}_{\mathrm{LV}}$	LV end-diastolic pressure	R^2	0.9865	0.9912	0.9888	0.9885	0.9790	0.9868
		ISE	93.65	96.83	92.06	91.27	87.30	92.22
p_{LV}^{IVC}	LV pressure at the end of IVC	R^2	0.9904	0.9906	0.9931	0.9937	0.9899	0.9916
		ISE	92.86	94.44	94.44	98.41	88.89	93.81
p_{LV}^{max}	LV peak pressure	R^2	0.9991	0.9996	0.9996	0.9996	0.9997	0.9995
		ISE	92.06	96.03	96.03	96.03	95.24	95.08
$\mathrm{ESV}_{\mathrm{LV}}$	LV end-systolic volume	R^2	0.9998	0.9998	0.9998	0.9999	0.9998	0.9998
		ISE	97.62	96.83	98.41	99.21	99.21	98.25
p_{LV}^{ej}	LV pressure at the end of ejection	R^2	0.9848	0.9817	0.9827	0.9867	0.9785	0.9829
		ISE	97.62	92.06	99.21	99.21	97.62	97.14
**Right ventricle outputs**								
$\mathrm{EDV}_{\mathrm{RV}}$	RV end-diastolic volume	R^2	0.9998	0.9998	0.9998	0.9997	0.9997	0.9998
		ISE	98.41	98.41	99.21	96.03	97.62	97.94
$\mathrm{EDP}_{\mathrm{RV}}$	RV end-diastolic pressure	R^2	0.9906	0.9951	0.9957	0.9959	0.9931	0.9941
		ISE	94.44	95.24	96.03	95.24	92.06	94.60
p_{RV}^{IVC}	RV pressure at the end of IVC	R^2	0.9746	0.9797	0.9785	0.9799	0.9733	0.9772
		ISE	94.44	94.44	93.65	97.62	95.24	95.08
p_{RV}^{max}	RV peak pressure	R^2	0.9992	0.9994	0.9995	0.9994	0.9995	0.9994
		ISE	92.06	92.86	96.83	96.83	94.44	94.60
$\mathrm{ESV}_{\mathrm{RV}}$	RV end-systolic volume	R^2	0.9983	0.9978	0.9975	0.9983	0.9981	0.9980
		ISE	92.06	92.86	96.83	93.65	94.44	93.97
p_{RV}^{ej}	RV pressure at the end of ejection	R^2	0.9946	0.9944	0.9945	0.9941	0.9931	0.9941
		ISE	97.62	96.03	94.44	96.83	94.44	95.87
$\mathrm{EDV}_{\mathrm{LA}}^{\mathrm{a}}$	LA max volume during the a-wave	R^2	0.9984	0.9988	0.9983	0.9989	0.9979	0.9985
		ISE	95.24	96.83	92.06	96.03	94.44	94.92
$p_{LA}^{\mathrm{max,a}}$	LA peak pressure during the a-wave	R^2	0.9982	0.9977	0.9948	0.9981	0.9985	0.9975
		ISE	97.62	96.83	91.27	96.83	98.41	96.1
$\mathrm{ESV}_{\mathrm{LA}}^{\mathrm{a}}$	LA min volume during the a-wave	R^2	0.9960	0.9974	0.9982	0.9980	0.9972	0.9974
		ISE	94.44	93.65	96.83	94.44	93.65	94.60
$\mathrm{EDV}_{\mathrm{LA}}^{\mathrm{v}}$	LA max volume during the v-wave	R^2	0.9981	0.9982	0.9981	0.9986	0.9982	0.9982
		ISE	97.62	97.62	96.03	98.41	95.24	96.98
$p_{LA}^{\mathrm{max,v}}$	LA peak pressure during the v-wave	R^2	0.9977	0.9978	0.9978	0.9982	0.9972	0.9977
		ISE	99.21	92.06	96.03	96.83	93.65	95.56
$\mathrm{ESV}_{\mathrm{LA}}^{\mathrm{v}}$	LA min volume during the v-wave	R^2	0.9984	0.9983	0.9986	0.9987	0.9975	0.9983
		ISE	94.44	93.65	94.44	97.62	95.24	95.08
$p_{LA}^{\mathrm{min,v}}$	LA min pressure during the v-wave	R^2	0.9982	0.9980	0.9978	0.9982	0.9974	0.9979
		ISE	96.03	95.24	95.24	95.24	94.44	95.24
**Right atrial outputs**								
$\mathrm{EDV}_{\mathrm{RA}}^{\mathrm{a}}$	RA max volume during the a-wave	R^2	0.9878	0.9919	0.9896	0.9918	0.9898	0.9902
		ISE	96.03	96.03	92.86	96.03	96.03	95.40
$p_{RA}^{\mathrm{max,a}}$	RA peak pressure during the a-wave	R^2	0.9923	0.9951	0.9930	0.9954	0.9950	0.9942
		ISE	94.44	94.44	90.48	96.83	92.06	93.65
$\mathrm{ESV}_{\mathrm{RA}}^{\mathrm{a}}$	RA min volume during the a-wave	R^2	0.9270	0.9489	0.9005	0.9699	0.9497	0.9392
		ISE	90.48	94.44	92.86	96.83	95.24	93.97
$\mathrm{EDV}_{\mathrm{RA}}^{\mathrm{v}}$	RA max volume during the v-wave	R^2	0.9953	0.9975	0.9965	0.9976	0.9976	0.9969
		ISE	95.24	95.24	93.65	96.83	96.83	95.56
$p_{RA}^{\mathrm{max,v}}$	RA peak pressure during the v-wave	R^2	0.9953	0.9981	0.9952	0.9982	0.9978	0.9969
		ISE	95.24	95.24	94.44	99.21	96.83	96.19
$\mathrm{ESV}_{\mathrm{RA}}^{\mathrm{v}}$	RA min volume during the v-wave	R^2	0.9851	0.9918	0.9826	0.9920	0.9858	0.9875
		ISE	92.86	96.03	90.48	95.24	92.86	93.49
$p_{RA}^{\mathrm{min,v}}$	RA min pressure during the v-wave	R^2	0.9792	0.9900	0.9798	0.9907	0.9842	0.9848
		ISE	91.27	96.03	92.86	95.24	92.06	93.49

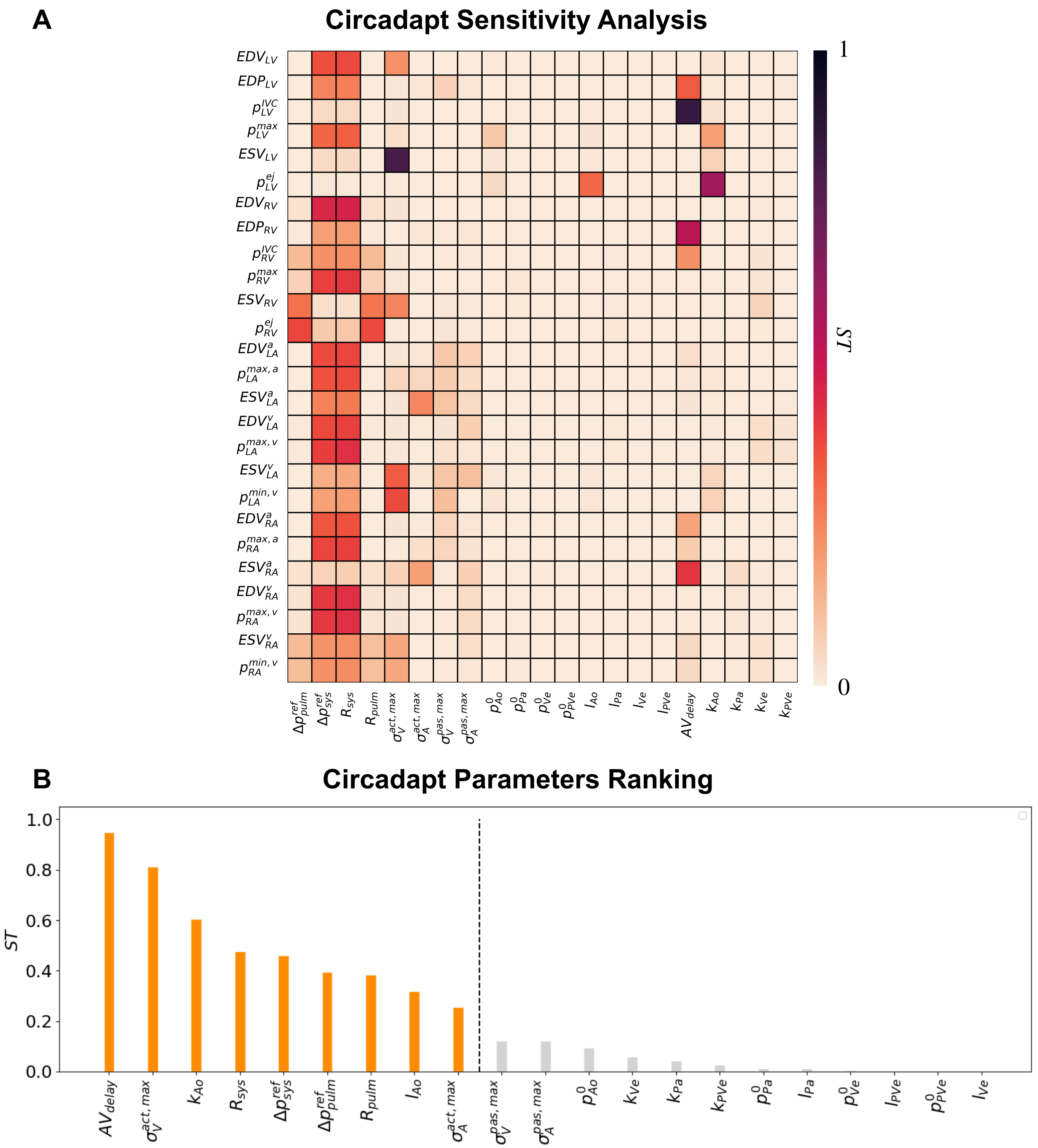

Fig 3. Sensitivity analysis results. A Heatmap of the total effect of the parameters (x-axis) on the outputs (y-axis). **B** Barplot of the maximum total effect of each parameter over all outputs. The parameters are ranked from most to least important. The dashed line separates important (orange) and unimportant (gray) parameters.

References

1. Walmsley J, Arts T, Derval N, Bordachar P, Cochet H, Ploux S, et al. Fast simulation of mechanical heterogeneity in the electrically asynchronous heart using the multipatch module. PLoS computational biology. 2015;11(7):e1004284–e1004284.

2. Augustin CM, Gsell MA, Karabelas E, Willemen E, Prinzen FW, Lumens J, et al. A computationally efficient physiologically comprehensive 3D–0D closed-loop model of the heart and circulation. Computer methods in applied mechanics and engineering. 2021;386:114092.
