# Supplementary material for: Cell to whole organ global sensitivity analysis on a four-chamber heart electromechanics model using Gaussian processes emulators
Source: PLoS Comput Biol. 2023 Jun 26;19(6):e1011257. doi: 10.1371/journal.pcbi.1011257 (PMC10328347; doi:10.1371/journal.pcbi.1011257)
Supplement: S7 File — Detailed explanation of how we constructed the Saltelli samples for the GSA. (PDF) [file pcbi.1011257.s007.pdf]

Saltelli sampling construction

In order to compute the total effects S_T using the Saltelli method, a Saltelli sampling needs to be constructed. Starting from a base sequence made of two independent matrices $\mathbf{A} = (a_{ij})$ and $\mathbf{B} = (b_{ij})$ of size $\mathrm{N}_{\mathrm{base}}\times\mathrm{D}$ each, where D is the number of parameters, the samples are re-organized to generate a radial sampling as explained in [1], where the $\mathrm{N}_{\mathrm{base}}(\mathrm{D}+2)$ resulting samples have the following structure:

$$\begin{bmatrix} a_{1,1} & a_{1,2} & a_{1,3} & \cdots & a_{1,D} \\ b_{1,1} & a_{1,2} & a_{1,3} & \cdots & a_{1,D} \\ a_{1,1} & b_{1,2} & a_{1,3} & \cdots & a_{1,D} \\ & & \vdots & & \\ a_{1,1} & a_{1,2} & a_{1,3} & \cdots & b_{1,D} \\ b_{1,1} & b_{1,2} & b_{1,3} & \cdots & b_{1,D} \\ a_{2,1} & a_{2,2} & a_{2,3} & \cdots & a_{2,D} \\ b_{2,1} & a_{2,2} & a_{2,3} & \cdots & a_{2,D} \\ a_{2,1} & b_{2,2} & a_{2,3} & \cdots & a_{2,D} \\ & & \vdots & & \\ a_{2,1} & a_{2,2} & a_{2,3} & \cdots & b_{2,D} \\ b_{2,1} & b_{2,2} & b_{2,3} & \cdots & b_{2,D} \\ & & \vdots & & \end{bmatrix}$$

The base sequence for the Saltelli sampling was constructed using either a Sobol sequence or a Latin hypercube design with $\mathrm{N}_{\mathrm{base}}\times 2\mathrm{D}$ samples, where D is the number of parameters and $\mathrm{N}_{\mathrm{base}}$ is set to 1000 unless otherwise specified.

References

1. Campolongo F, Saltelli A, Cariboni J. From screening to quantitative sensitivity analysis. A unified approach. Computer Physics Communications. 2011;182(4):978–988.
