# Supplementary material for: Cell to whole organ global sensitivity analysis on a four-chamber heart electromechanics model using Gaussian processes emulators
Source: PLoS Comput Biol. 2023 Jun 26;19(6):e1011257. doi: 10.1371/journal.pcbi.1011257 (PMC10328347; doi:10.1371/journal.pcbi.1011257)
Supplement: S8 File — We investigated how the choice of kernel affected GPE accuracy and GSA. The GPE training and GSA performed for the ToR-ORd model (S2 File) were repeated with a Matérn rather than an exponentiated quadratic kernel and the results between the two cases were compared. (PDF) [file pcbi.1011257.s008.pdf]

Gaussian processes emulators kernel comparison

To test the effect of using a different kernel for the emulators, we repeated Gaussian processes emulator (GPE) training for the ToR-ORd ventricular ionic model outputs with a Matérn rather than a radial basis function (RBF), or an exponentiated quadratic, kernel. For the meaning of model parameters and outputs, and parameter ranges, refer to Supplement S2.

As presented in the main manuscript, the GPEs used in this study had the following prior mean and covariance:

$$\mathbb{E}[f(\mathbf{x})] = \beta_0 + \sum_{i=1}^{D} \beta_i x_i \tag{1}$$

$$\mathbb{C}[f(\mathbf{x}), f(\mathbf{x}')] = k(\mathbf{x}, \mathbf{x}') \ , \tag{2}$$

where D is the number of input parameters $\mathbf{x} = (x_1, ..., x_D)$ and $f(\mathbf{x})$ is a scalar simulator output. In the main manuscript, $k(\mathbf{x}, \mathbf{x}')$ is a the RBF kernel [1]:

$$k_{\mathrm{RBF}}(\mathbf{x}, \mathbf{x}') = \exp\left(- \frac{||\mathbf{x} - \mathbf{x}'||^2}{2\sigma^2} \right) . \tag{3}$$

where σ is the length scale parameter.

In the results below, we retrained the GPEs with a Matérn kernel:

$$k_{\mathrm{M}}(\mathbf{x}, \mathbf{x}') = \frac{2^{1-\nu}}{\Gamma(\nu)} \left(\frac{\sqrt{2\nu}||\mathbf{x} - \mathbf{x}'||}{\sigma} \right)^{\nu} K_{\nu} \left(\frac{\sqrt{2\nu}||\mathbf{x} - \mathbf{x}'||}{\sigma} \right) , \tag{4}$$

where σ and ν are the length scale parameter and the smoothness coefficient, respectively, and $\Gamma(\nu)$ and K_v are the gamma function and a modified Bessel function [1]. It is worth noting that when $\nu \longrightarrow \infty$, we obtain the RBF kernel.

Table 1 shows the coefficient of determination R^2 and independent standard error (ISE) obtained for a 5-fold cross-validation. We show values for an RBF (as in the original manuscript) and a Matérn kernel. As shown by the values in the table, the effect of the kernel on GPE accuracy is very small, with mean R^2 and the ISE score changing by less than 10^{-4} and 0.55%, respectively.

To ensure the kernel did not affect the global sensitivity analysis (GSA), we repeated the GSA using the trained GPEs with different kernels. Fig 1 shows the total effects obtained for all model parameters on all four model outputs, obtained with the an RBF (orange) and a Matérn (blue) kernel. These results show that the GSA is unaffected by the choice of the kernel.

Table 1. GPE performance with different kernels. Coefficient of determination and independent standard error obtained for a 5-fold cross-validation during GPE training with a radial basis function (RBF) and a Matérn kernel.

Coefficient of determination R^2													
**Output**	**Meaning**	**fold-1**		**fold-2**		**fold-3**		**fold-4**		**fold-5**		**Mean**	
		**RBF**	**Matérn**	**RBF**	**Matérn**	**RBF**	**Matérn**	**RBF**	**Matérn**	**RBF**	**Matérn**	**RBF**	**Matérn**
Ca_{diast}	Diastolic calcium concentration	0.9944	0.9956	0.9925	0.9929	0.9870	0.9886	0.9926	0.9918	0.9877	0.9882	0.9908	0.9914
Ca_{ampl}	Transient amplitude	0.9907	0.9901	0.9720	0.9715	0.9829	0.9826	0.9882	0.9885	0.9906	0.9909	0.9849	0.9848
TTP	Time to peak	0.9902	0.9901	0.9873	0.9853	0.9896	0.9907	0.9880	0.9893	0.9906	0.9908	0.9891	0.9892
RT90	Time to reach 90% decay	0.9972	0.9968	0.9964	0.9963	0.9956	0.9964	0.9974	0.9973	0.9974	0.9973	0.9968	0.9968
**Independent standard error (ISE)**													
**Output**	**Meaning**	**fold-1**		**fold-2**		**fold-3**		**fold-4**		**fold-5**		**Mean**	
		**RBF**	**Matérn**	**RBF**	**Matérn**	**RBF**	**Matérn**	**RBF**	**Matérn**	**RBF**	**Matérn**	**RBF**	**Matérn**
Ca_{diast}	Diastolic calcium concentration	99.77	99.77	98.62	98.39	98.62	98.39	98.85	97.70	98.16	98.39	98.80	98.53
Ca_{ampl}	Transient amplitude	99.54	99.31	98.16	97.47	98.62	97.93	98.85	98.39	99.77	99.08	98.99	98.44
TTP	Time to peak	98.85	98.39	98.39	97.01	98.85	98.62	98.39	98.16	98.85	99.08	98.67	98.25
RT90	Time to reach 90% decay	99.54	99.31	98.62	97.93	98.85	99.08	99.54	98.85	99.31	99.31	99.17	98.90

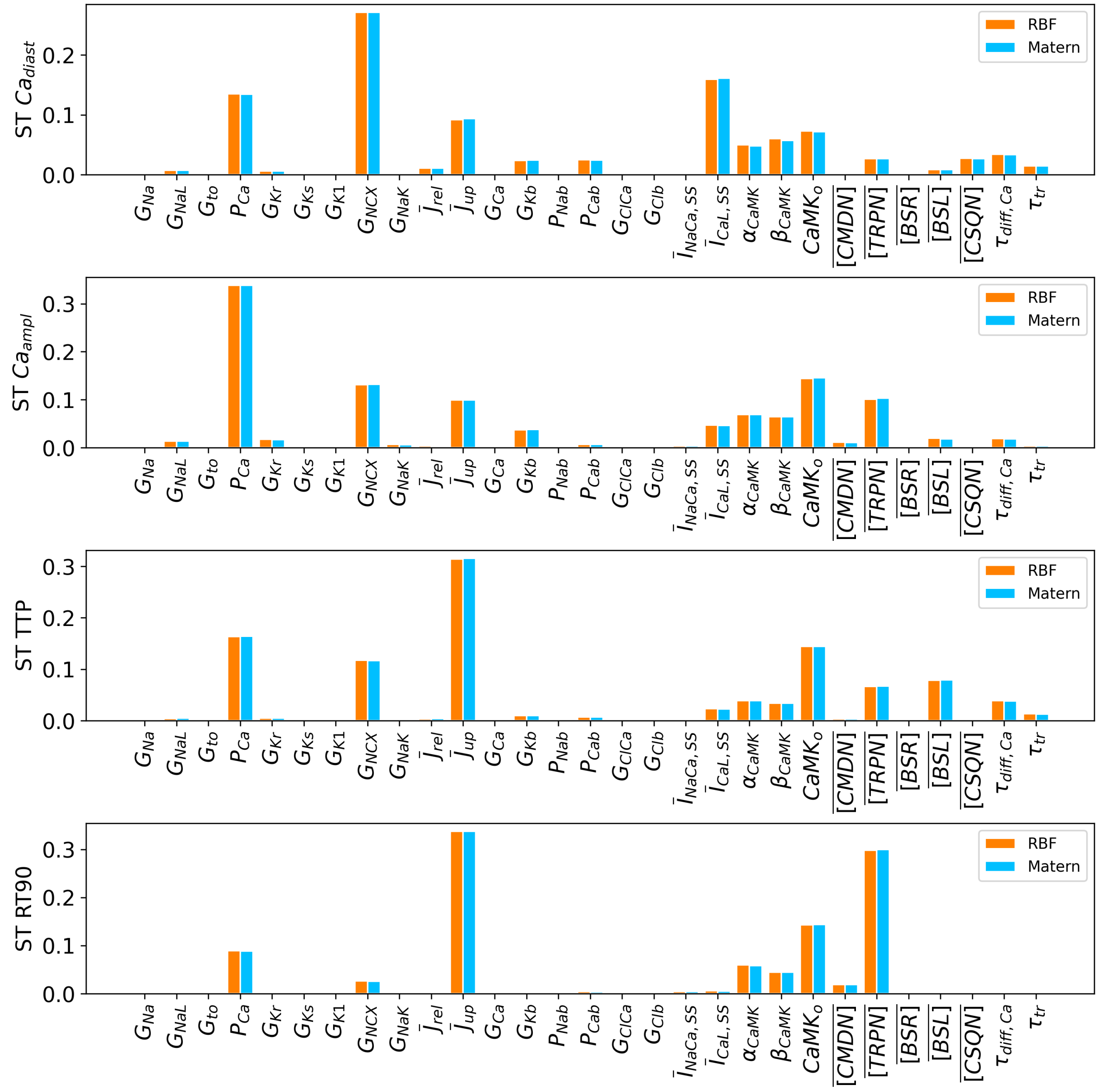

Fig 1. Total effect comparison with different kernels. The total effects of all 29 input parameters on all four model outputs is shown for a GSA performed using GPEs trained with an RBF (orange) and a Matérn (blue) kernel.

References

1. Rasmussen CE, Williams CK, et al. Gaussian processes for machine learning. vol. 1. Springer; 2006.
