# Supplementary material for: Cell to whole organ global sensitivity analysis on a four-chamber heart electromechanics model using Gaussian processes emulators
Source: PLoS Comput Biol. 2023 Jun 26;19(6):e1011257. doi: 10.1371/journal.pcbi.1011257 (PMC10328347; doi:10.1371/journal.pcbi.1011257)
Supplement: S9 File — We performed the GSA on the electrophysiology tissue model with two different ventricular fibre orientation to investigate the effect of myofibre arrengement on electrophysiology simulations and GSA. We also repeated a passive inflation and a four-chamber electromechanics simulation to quantify the effect of ventricular fibres on mechanics model outputs. (PDF) [file pcbi.1011257.s009.pdf]

The effect of ventricular fibre orientation

We wanted to investigate the effect of ventricular fibre orientation on the simulations and on the global sensitivity analysis (GSA) results. The fibre orientation in the ventricles was changed from $+60°/-60°$ to $+50°/-50°$ and $+70°/-70°$ from endocardium to epicardium. We repeated all Eikonal simulations performed for the tissue electrophysiology GSA (see Supplement S4), re-trained the emulators and performed the GSA again with the two new fibre orientations. The results were then compared to the GSA performed with the $+60°/-60°$ fibre orientation we originally used (see Section 1).

Since it would be too computationally expensive to re-run all mechanics simulations with different fibre directions, we considered one of the inflation simulations and one of the cycle simulations used to train the emulators for the passive mechanics GSA (Supplement S5å) and for the four-chamber whole organ GSA (main manuscript), respectively. These simulations were repeated with the new fibre orientations ($+50°/-50°$ and $+70°/-70°$) and the results compared to the original simulations with $+60°/-60°$ fibre direction (Section 2).

1 Tissue electrophysiology GSA

We compared the total ventricular activation times (TAT_V) obtained with $+50°/-50°$ and $+70°/-70°$ ventricular fibre orientation to those obtained with baseline fibres ($+60°/-60°$). Fig 1 shows the absolute (left) and percentage (right) differences for both cases. Shallow fibres ($+50°/-50°$) led to longer activation times (10±6 ms), corresponding to an absolute percentage difference of 5.9±2.2%. On the other hand, steeper fibres ($+70°/-70°$) caused a decrease in total ventricular activation (-7.7±4.1 ms, absolute percentage difference: 4.4±0.8%). This shows that altering ventricular fibre orientation leads to small changes in the total ventricular activation.

Fig 2 shows a comparison of the total effects of all electrophysiology parameters on TAT_V and on the total atrial activation time (TAT_A). Blue, orange and green bars represent total effects computed with $+50°/-50°$, $+60°/-60°$ and $+70°/-70°$, respectively. As expected, the sensitivity indices on atrial activation times do not change, because we only altered the ventricular fibres. The contribution of myocardial conduction velocity of the ventricles ($CV_{f,V}$) and the scaling factor of fast endocardial conduction (k_{FEC}) changed depending on the fibres. When the fibres are shallower ($+50°/-50°$), myocardial conduction velocity plays a more important role than the fast endocardial layer in total activation of the ventricles. On the other hand, when the fibres are steeper ($+70°/-70°$), fast endocardial conduction is more important than the myocardial conduction velocity.

2 Mechanics simulations

Table 1 shows the results obtained for the mechanics simulations with different ventricular fibre orientations. In the passive inflation, the differences in inflated volumes were below 0.2%, while differences in mean ventricular fibre strains were bigger, 10.1% and 7.3% for shallow and steep fibres, respectively. Ventricular fibres caused small differences in left and right ventricular outputs, with the biggest differences found in the end-diastolic pressures (Table 1). Due to atrio-ventricular coupling, ventricular fibres cause changes in peak in atrial pressure. The left atrial peak pressure changed by 5.1% and 3.4% with shallow and steep fibres, respectively.

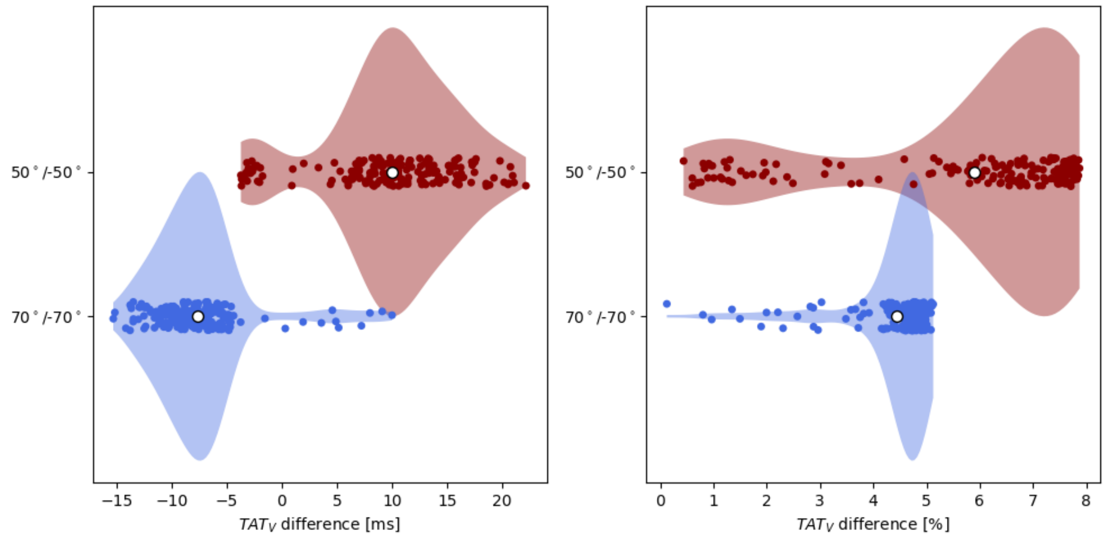

Fig 1. The effect of ventricular fibres on activation metrics. The difference between total ventricular activation times (TAT_v) obtained with $+60°/-60°$ fibre orientation and with shallow ($+50°/-50°$, red) and steep ($+70°/-70°$, blue) fibres. Left and right plots show the absolute and the percentage differences, respectively, and the white dots indicate the mean differences.

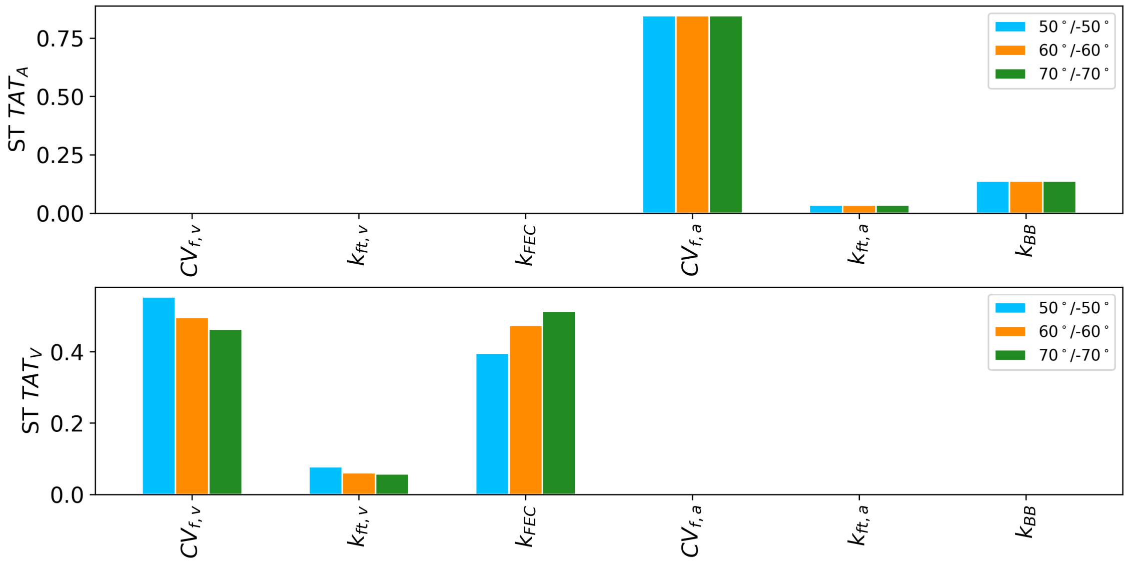

Fig 2. The effect of ventricular fibres on electrophysiology sensitivity indices. Total effects on total atrial (top) and ventricular (bottom) activation obtained with different fibre orientations.

Table 1. The effect of fibre orientation on the mechanics simulations. Model outputs obtained with shallow ($+50°/-50°$) and steep fibres ($+70°/-70°$) compared to the baseline fibre orientation ($+60°/-60°$) used in the main manuscript. The top and the bottom sections show outputs for passive inflation and for a four-chamber electromechanics simulations.

	**Baseline**	**50°/-50°**	**70°/-70°**
**Passive inflation**			
**Model output**		**Output value (% error vs baseline)**	**Output value (% error vs baseline)**
V_{LV}^{max} [mL]	192.987	193.278 (0.2%)	192.792 (0.1%)
V_{RV}^{max} [mL]	156.392	156.488 (0.1%)	156.270 (0.1%)
V_{LA}^{max} [mL]	165.782	165.814 (0.0%)	165.754 (0.0%)
V_{RA}^{max} [mL]	145.850	145.889 (0.0%)	145.810 (0.0%)
$\overline{E_{ff,V}}$ [%]	2.044	2.251 (10.1%)	1.895 (7.3%)
$\overline{E_{ff,A}}$ [%]	5.279	5.282 (0.1%)	5.278 (0.0%)
**Four-chamber electromechanics**			
**Left ventricle**			
EDV [mL]	150.883	153.168 (1.5%)	153.126 (1.5%)
EDP [mmHg]	5.63	5.931 (5.3%)	5.63 (4.1%)
ESV [mL]	108.275	110.774 (2.3%)	110.727 (2.3%)
p_{max} [mmHg]	113.794	113.024 (0.7%)	112.741 (0.9%)
dp/dt_{max} [mmHg/s]	1502.0	1488.0 (0.6%)	1461.0 (2.4%)
dp/dt_{min} [mmHg/s]	-671.0	-662.0 (1.0%)	-653.0 (2.4%)
**Right ventricle**			
EDV [mL]	118.307	119.532 (1.0%)	119.269 (0.8%)
EDP [mmHg]	3.07	2.944 (4.1%)	3.04 (1.0%)
ESV [mL]	79.445	80.245 (1.0%)	80.586 (1.4%)
p_{max} [mmHg]	25.476	25.48 (1.2%)	25.40 (0.3%)
dp/dt_{max} [mmHg/s]	352.0	356.0 (1.1%)	341.4 (3.0%)
dp/dt_{min} [mmHg/s]	-136.0	-140.0 (2.9%)	-136.0 (0.0%)
**Left atrium**			
EDV [mL]	178.729	181.026 (1.3%)	180.795 (1.2%)
ESV [mL]	144.842	146.078 (0.9%)	145.998 (0.8%)
V_{max} v-wave [mL]	182.703	184.219 (0.8%)	184.281 (0.9%)
p_{max} [mmHg]	5.311	5.585 (5.1%)	5.494 (3.4%)
**Right atrium**			
EDV [mL]	151.703	150.731 (0.6%)	150.75 (0.6%)
ESV [mL]	123.093	121.719 (1.1%)	123.857 (0.3%)
V_{max} v-wave [mL]	157.861	155.757 (1.3%)	158.238 (0.2%)
p_{max} [mmHg]	3.316	3.251 (2.0%)	3.285. (0.9%)

3 Conclusion

Despite the differences found on the electrophysiology simulations and GSA due to ventricular fibre direction, the conduction anisotropy ratio of both ventricles ($k_{ft,v}$) and atria ($k_{ft,a}$) remained the least important parameters. Therefore, these would have been excluded from further analysis regardless of the fibre direction. While we could not investigate the effect of fibre orientation on the mechanics GSA due to computational costs, we found that ventricular myofibres only caused small changes in model outputs. Given the uncertainty due to numerical errors (Supplement S1) and the emulators uncertainty, we can conclude that the results presented in the manuscript would not change if we used different ventricular fibre angles.
