# Supplementary material for: Cell to whole organ global sensitivity analysis on a four-chamber heart electromechanics model using Gaussian processes emulators
Source: PLoS Comput Biol. 2023 Jun 26;19(6):e1011257. doi: 10.1371/journal.pcbi.1011257 (PMC10328347; doi:10.1371/journal.pcbi.1011257)
Supplement: S10 File — We performed the GSA on the electrophysiology tissue model altering the size of the fast endocardial conducting layer of the ventricles and the Bachmann bundle. (PDF) [file pcbi.1011257.s010.pdf]

The effect fast conducting regions size on the electrophysiology model

We wanted to investigate the effect of the size of the fast endocardial conduction (FEC) layer and the Bachmann bundle (BB) area on the electrophysiology simulations and on the global sensitivity analysis (GSA) on the tissue electrophysiology model. The FEC and BB areas were increased by 2.5 mm and 5 mm (Fig 1. For both cases, we repeated all Eikonal simulations we ran for the tissue electrophysiology GSA (Suppelement S4), re-trained the emulators and performed the GSA to investigate the impact of regions size on model outputs and on the GSA.

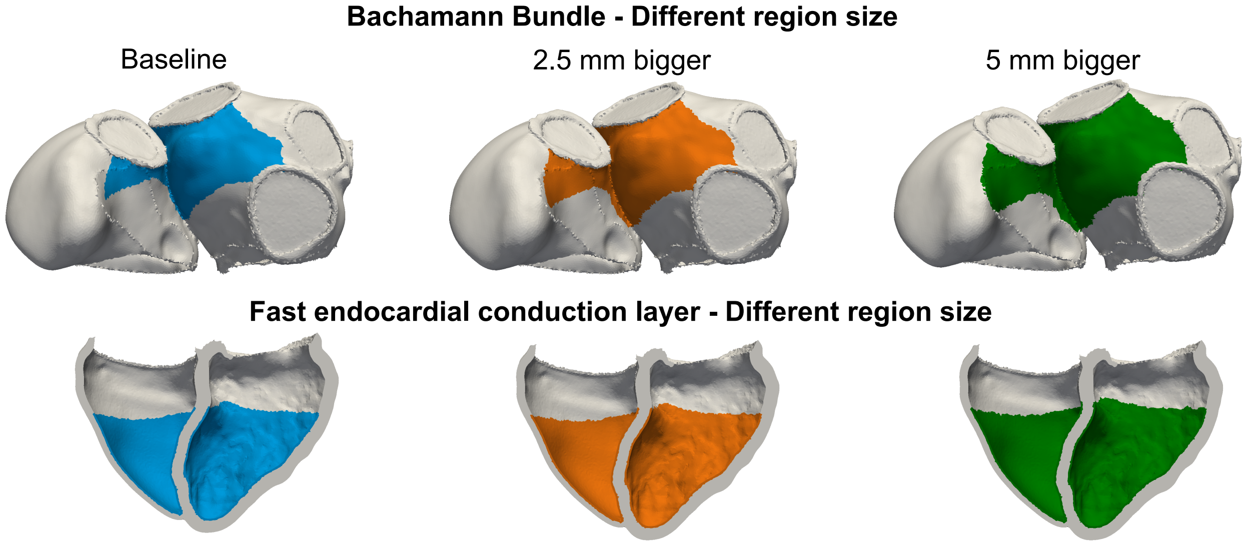

Fig 1. Different sizes for the fast conducting regions. The original regions used in the main manuscript are shown on the left in blue, while the middle and the right figures show 2.5 mm and 5 mm bigger regions.

Fig 2 shows the absolute and percentage difference on the total atrial (TAT_A) and ventricular (TAT_V) activation times obtained with the baseline fast conducting regions and with 2.5 mm and 5 mm bigger regions. As expected, having bigger FEC and BB leads to shorter activation times (2.5 mm: BLA±BLA ms, BLA±BLA %; 5 mm: BLA±BLA ms, BLA±BLA %). These changes in activation times led to small differences in the total effects in the GSA. Fig 3 shows the total effects of all electrophysiology parameters on on TAT_A and TAT_V, with blue, orange and green bars indicating the results obtained with baseline regions, and 2.5 mm and 5 mm bigger regions, respectively. When the FEC was 2.5 mm and 5 mm bigger, the contribution of the bulk myocardial conduction velocity in the ventricles ($CV_{f,V}$) to changes in TAT_V decreased, in favour of an increased contribution of the conduction velocity ratio in the FEC (k_{FEC}).

Conclusion

The size of the fast conducting regions leads to small changes in simulated activation times. The results of the tissue electrophsyiology GSA remain almost unaltered, and the changes we found in the resulting total effects would not have changed the parameters we selected for further analysis. The conclusions of our study were therefore not dependent on the size of the fast conducting regions.

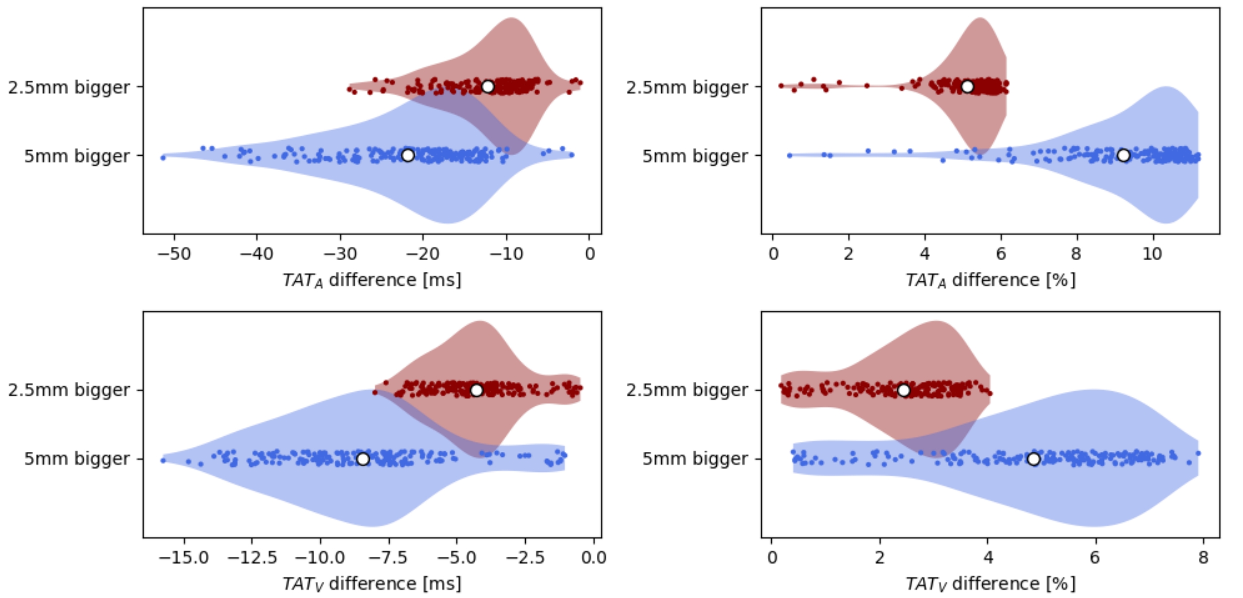

Fig 2. The effect of the size of fast conducting regions on activation times. The images show plots of the absolute (left) and the percentage (right) differences in total atrial (TAT_A, top) and ventricular (TAT_V, bottom) activation times caused by an increase of the fast conducting regions by 2.5 mm (red) and 5 mm (blue).

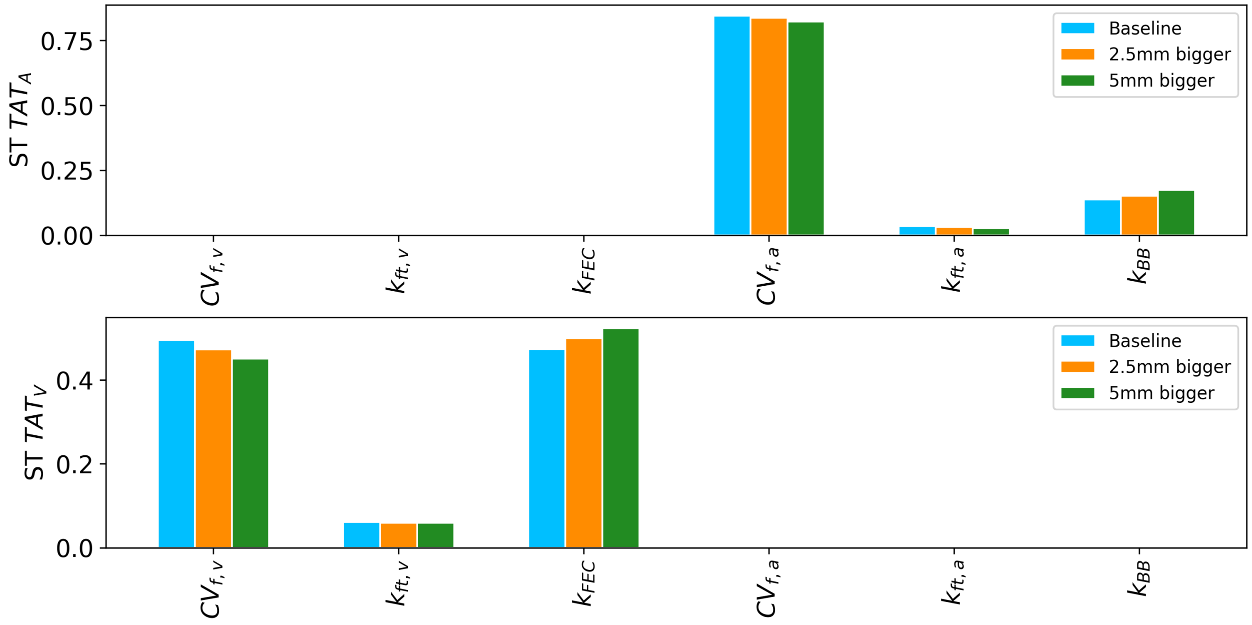

Fig 3. The effect of the size of fast conducting regions on sensitivity indices. Comparison between the total effects computed with different sizes of the fast conducting regions: baseline (blue), 2.5 mm bigger (orange) and 5 mm bigger (green).
