# Supplementary material for: Cell to whole organ global sensitivity analysis on a four-chamber heart electromechanics model using Gaussian processes emulators
Source: PLoS Comput Biol. 2023 Jun 26;19(6):e1011257. doi: 10.1371/journal.pcbi.1011257 (PMC10328347; doi:10.1371/journal.pcbi.1011257)
Supplement: S11 File — Schematic of how the non-implausible areas from the HM of the sub-models were used to construct the training samples for the GPEs at the whole organ scale. (PDF) [file pcbi.1011257.s011.pdf]

Non-implausible region extraction

The last history matching (HM) wave on the ToR-ORd-Land model (Supplement S2) resulted in N=90906 plausible samples that provide us with a physiological ventricular calcium and active tension transient at the cellular level. Similarly, the last HM wave on the Courtemanche-Land (Supplement S3) and on the tissue electrophysiology (Supplement S4) models provided us with N=148527 and N=99723, respectively, that led to physiological atrial calcium and active tension transient and total activation times (Fig 1, left, black samples). The remaining 15 parameters were sampled within their range (see main manuscript) with a latin hypercube sampling.

In order to build the training set for the emulators, we need to combine these samples into one dataset. We used `psa_select` from the python library `diversipy` [1] to select a sub-set of the input samples that best represent them, while approximating a uniform distribution and therefore ensuring optimal coverage of the parameter space. This function was first used to select the representatives for the Courtemanche-Land and the electrophysiology sub-models to have the same number of samples as the ToR-ORd-Land model (Fig 1, centre). The extracted samples from all sub-models and the latin hypercube samples from the remaining 15 parameters were combined in a unique dataset and `psa_select` was applied again to select N=500 best representatives. This provided us with N=500 samples (Fig 1, right) that we used for emulator training.

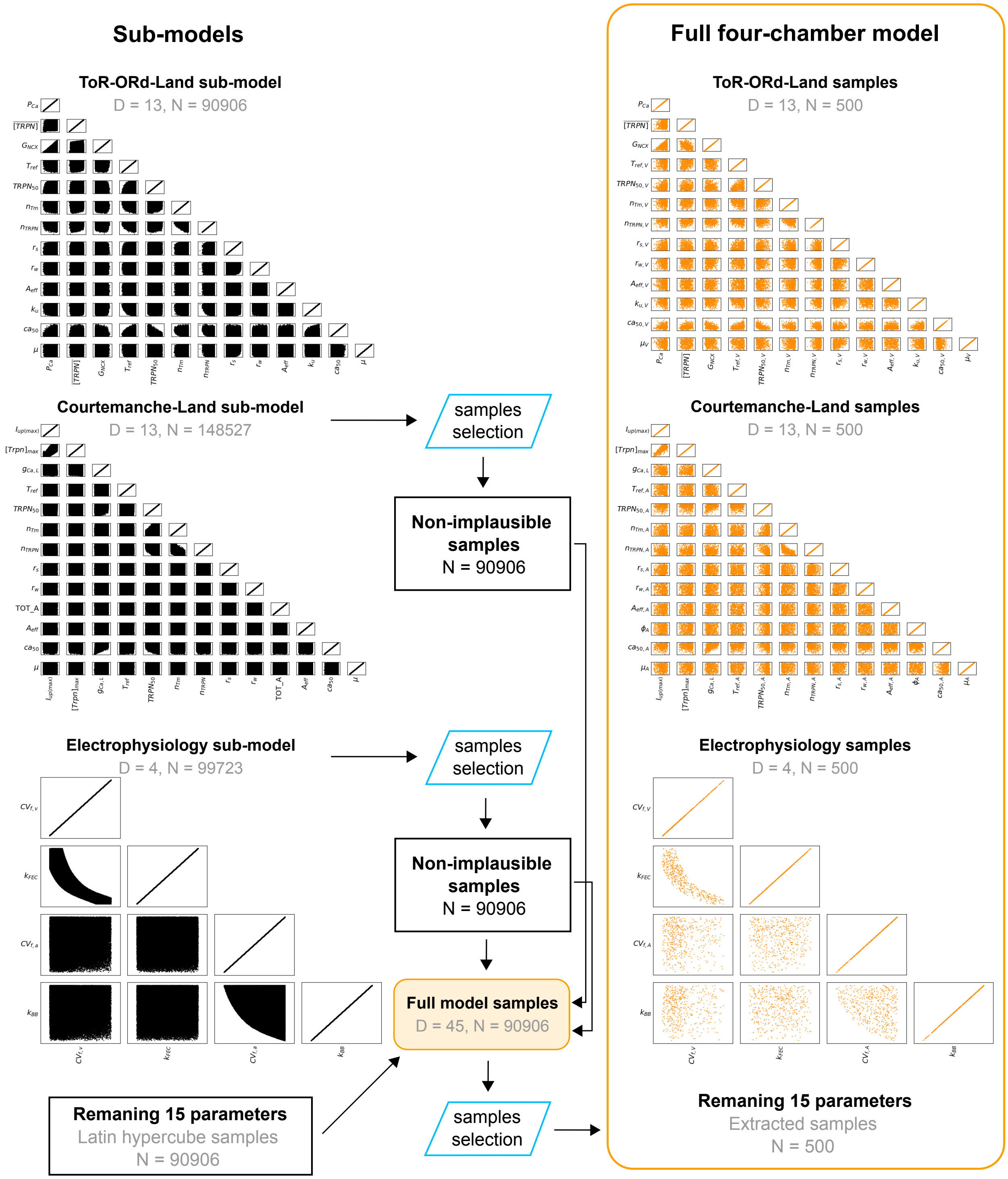

Fig 1. Schematic for emulator training samples from sub-models. In the figure above, D and N always indicate the number of parameters and the number of samples. Black samples and boxes indicate analysis done on the sub-models, while blue boxes indicate when the function `psa_select` was used to extract a sub-set of the original samples that are as uniformly distributed as possible. Finally, orange boxes indicate analysis done for the four-chamber model.

References

1. Wessing S. Two-stage methods for multimodal optimization. Dissertation, Dortmund, Technische Universität, 2015; 2015.
