# Supplementary material for: Cell to whole organ global sensitivity analysis on a four-chamber heart electromechanics model using Gaussian processes emulators
Source: PLoS Comput Biol. 2023 Jun 26;19(6):e1011257. doi: 10.1371/journal.pcbi.1011257 (PMC10328347; doi:10.1371/journal.pcbi.1011257)
Supplement: S12 File — We show the mean and standard deviation of the total effects computed for the whole organ sensitivity analysis obtained when sampling the posterior distribution of the emulators. (PDF) [file pcbi.1011257.s012.pdf]

The effect of emulators uncertainty on sensitivity indices

Fig 1-4 show the total effects computed for each whole organ biomarkers for each chamber. The error bars represent the standard deviation on the total effects computed from 1000 samples from the posterior distribution of the emulators. Since the standard deviation of the total effects is small, we can conclude that the emulator uncertainty has negligible effects on the results we presented in the manuscript.

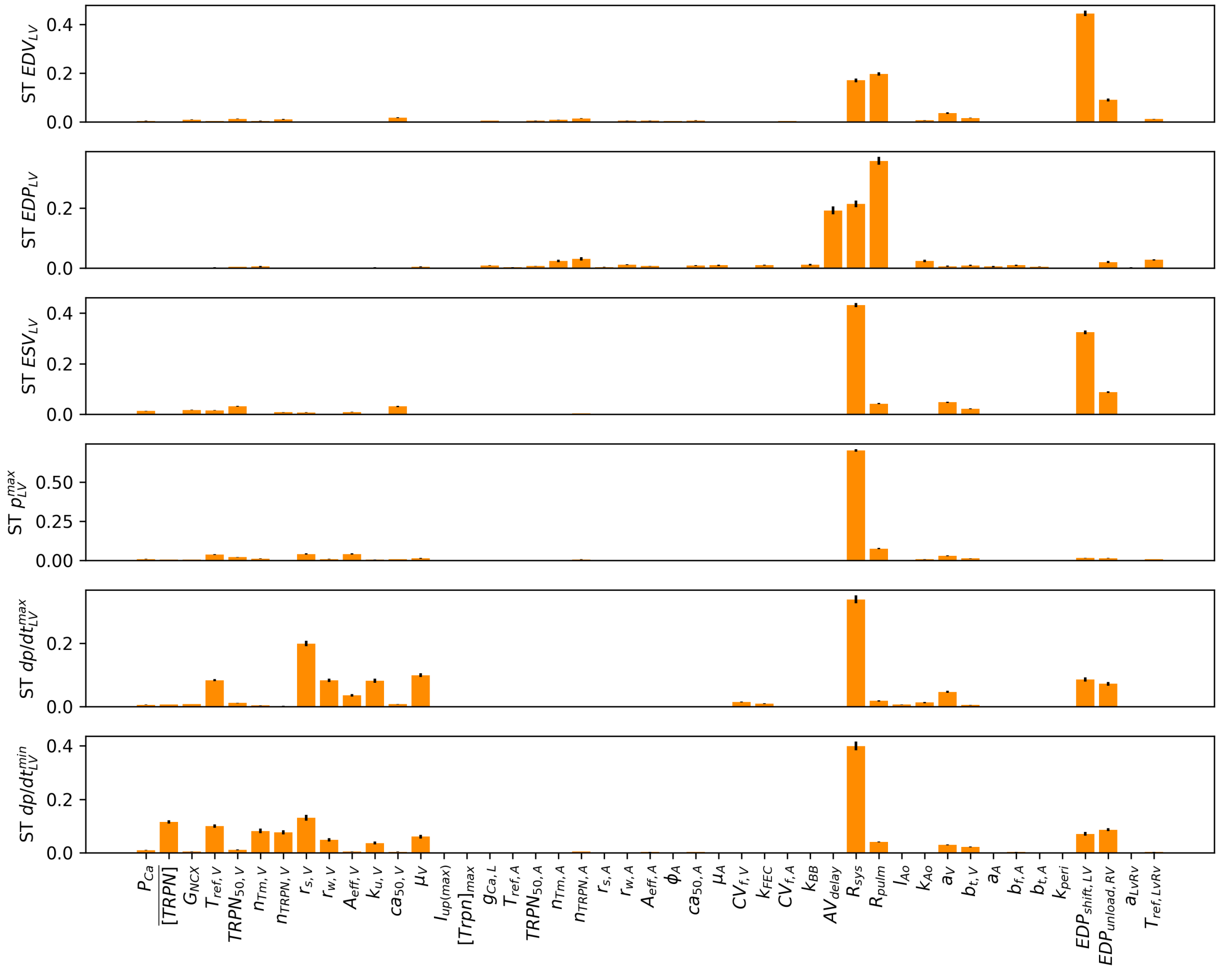

Fig 1. Total effects on left ventricle biomarkers. Barplot of the total effects on the whole-organ biomarkers computed for the left ventricle. The error bars represent the standard deviation of the total effects obtained from 1000 samples of the emulators posterior distribution.

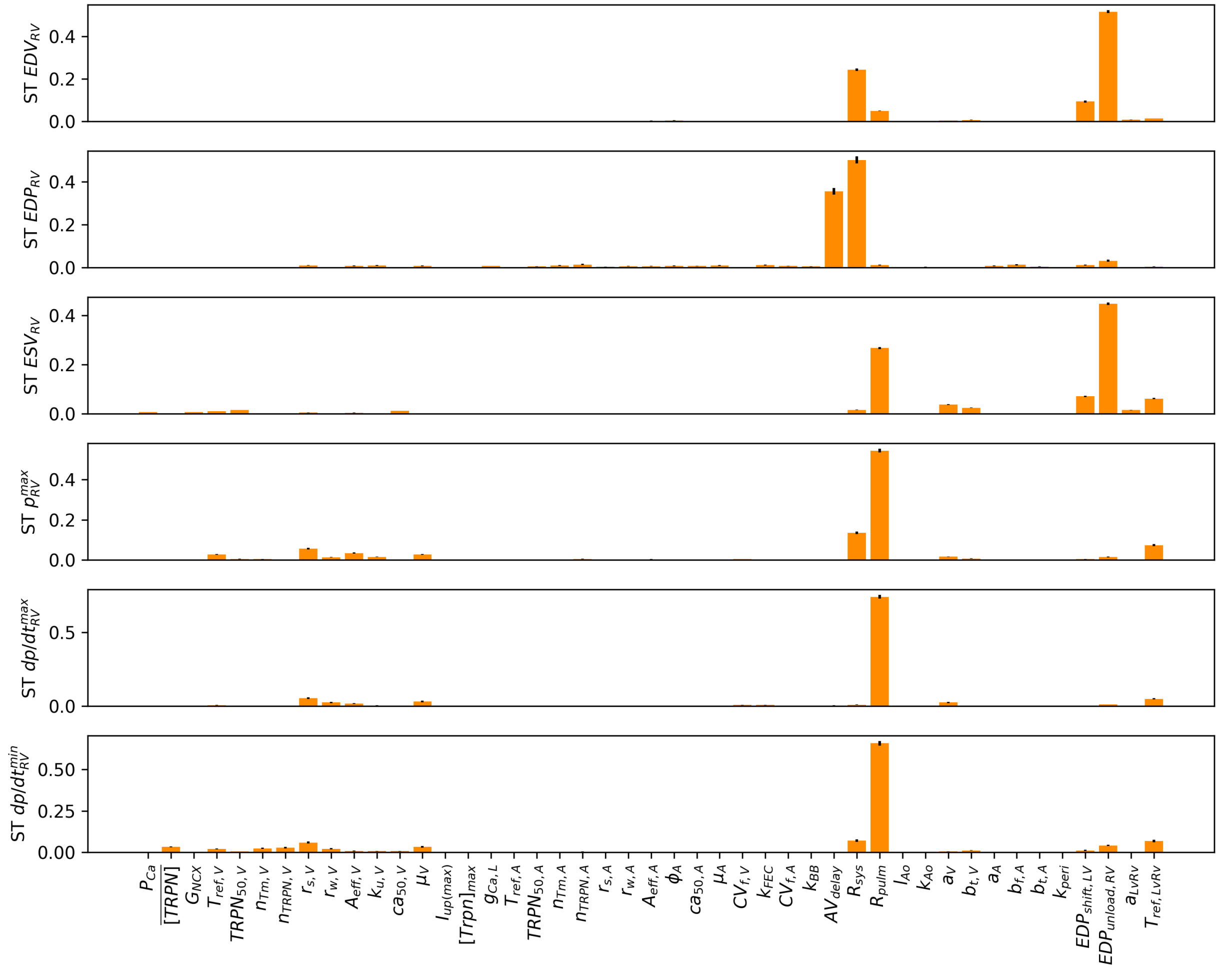

Fig 2. Total effects on right ventricle biomarkers. Barplot of the total effects on the whole-organ biomarkers computed for the right ventricle. The error bars represent the standard deviation of the total effects obtained from 1000 samples of the emulators posterior distribution.

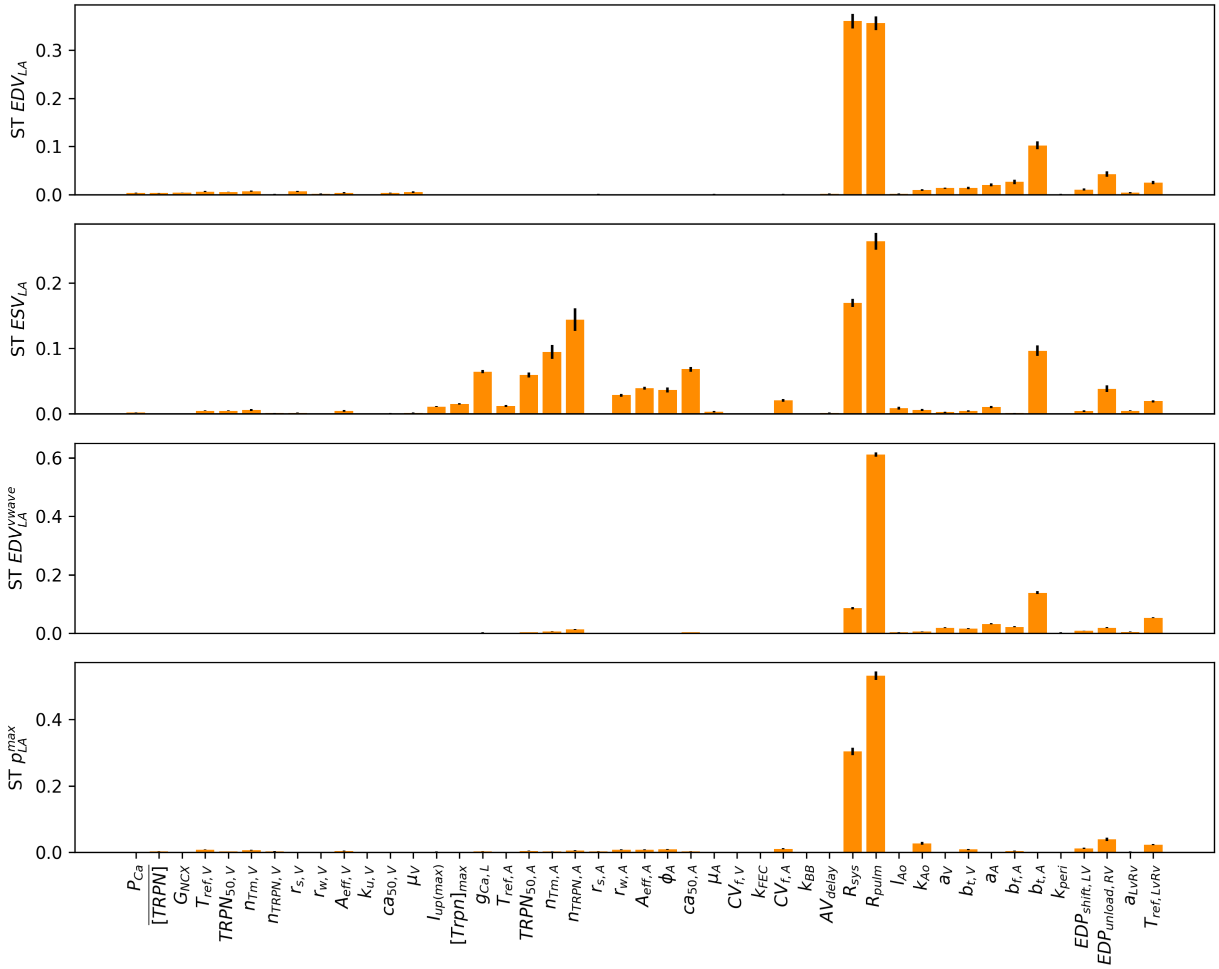

Fig 3. Total effects on left atrium biomarkers. Barplot of the total effects on the whole-organ biomarkers computed for the left atrium. The error bars represent the standard deviation of the total effects obtained from 1000 samples of the emulators posterior distribution.

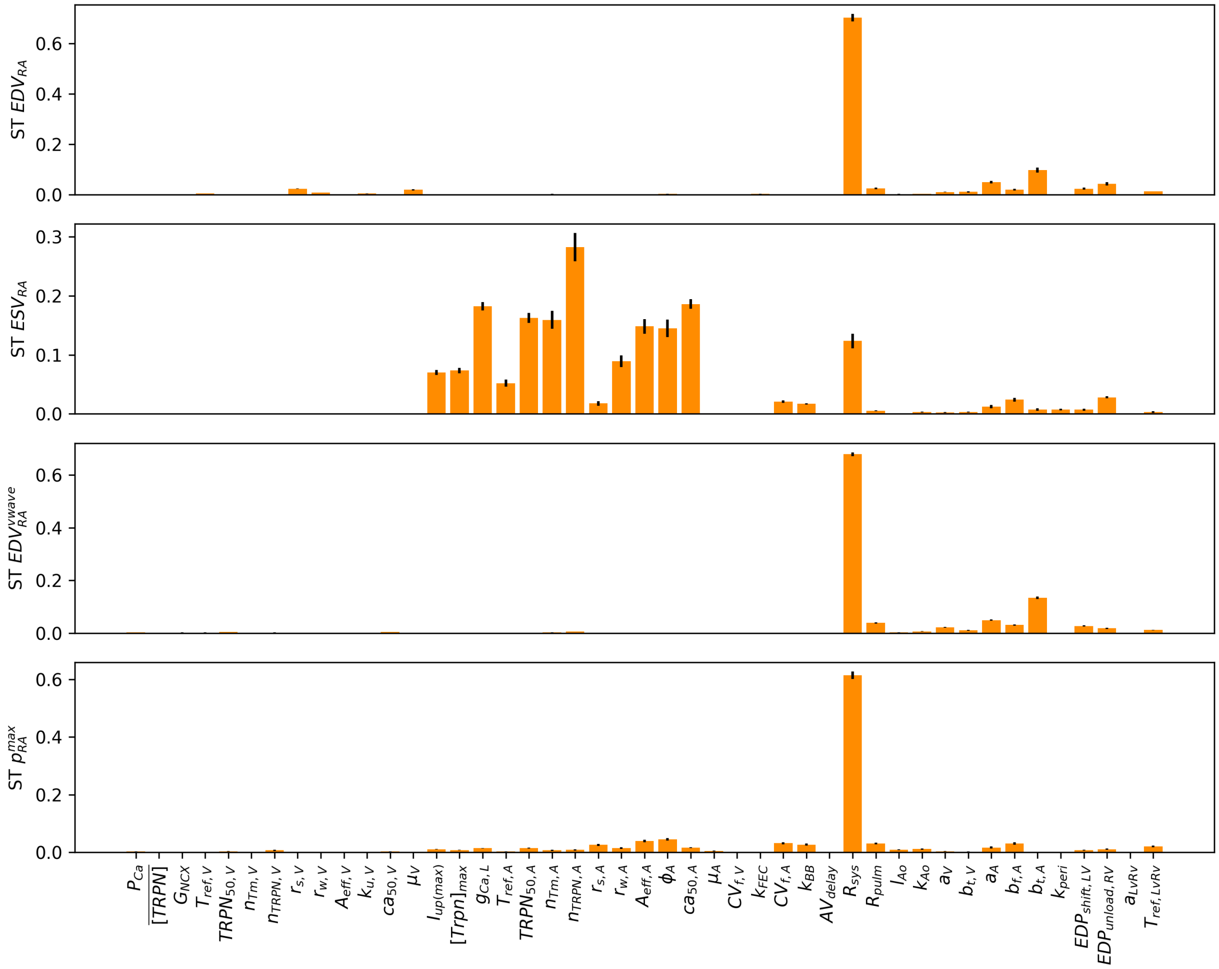

Fig 4. Total effects on right ventricle biomarkers. Barplot of the total effects on the whole-organ biomarkers computed for the right ventricle. The error bars represent the standard deviation of the total effects obtained from 1000 samples of the emulators posterior distribution.
